# New Cyclohexenols and Benzopyran Derivatives from Fungus *Aspergillus fumigatus* F15ZA56

**DOI:** 10.3390/jof12070504

**Published:** 2026-07-09

**Authors:** Ningning Shi, Junling Guo, Zhen Zhang, Feng Jing, Shuoyu Zhao, Yan Fu, Xinhua Lu, Yucheng Gu, Binliang Tong, Manli Zhang

**Affiliations:** 1 Hebei Key Laboratory of Innovative Drug Development and Evaluation, School of Pharmaceutical Sciences, Hebei Medical University, Shijiazhuang 050017, China; 18800970@hebmu.edu.cn (N.S.); 24033100172@stu.hebmu.edu.cn (J.G.); 13503225760@163.com (Z.Z.); jing4389@126.com (F.J.); 25034100681@stu.hebmu.edu.cn (S.Z.); 2 Core Facilities and Centers, Hebei Medical University, Shijiazhuang 050017, China; fuyan0228@hebmu.edu.cn; 3 New Drug Research & Development Center of North China Pharmaceutical Group Corporation, Shijiazhuang 052165, China; luxinhua89@yeah.net; 4 Syngenta Jealott’s Hill International Reasearch Centre, Bracknell RG12 6EY, Berkshire, UK; yucheng.gu@syngenta.com; 5 Department of Pharmacy, The First Affiliated Hospital of Hebei Medical University, Shijiazhuang 050000, China

**Keywords:** *Aspergillus fumigatus*, cyclohexenols, benzopyran derivatives, inhibitory activity of protein tyrosine phosphatases (PTPs)

## Abstract

A chemical study of the fungus *Aspergillus fumigatus* F15ZA56 resulted in the elucidation of eight previously undescribed cyclohexenols, aspergienynes R-Y (**1**, **5**–**11**), and three new benzopyran derivatives (**2**–**4**), together with two known analogues (**12**–**13**). The structures were determined based on HRESIMS and extensive NMR data. The absolute configurations of the chiral carbons in the new compounds were ultimately confirmed by ECD analysis. Bioactivity assays showed that compounds **4**, **12** and **13** had significant inhibitory activities against TCPTP, PTP1B, and MEG2 (IC_50_ 12.20–62.19 nM). Notably, compound **12** exhibited notable selectivity towards PTP1B (IC_50_ 12.20 nM) over the tested phosphatases, comparable to the reference inhibitor AC484 (9.12 nM).

## 1. Introduction

*Aspergillus fumigatus*, one of the most ubiquitous filamentous fungi, is known for producing a vast array of structurally novel secondary metabolites, including alkaloids [1,2,3,4,5,6], terpenoids [7,8,9,10,11], pyranones [12,13], peptides [14], polyketides [15], sulfur-containing phenolic compounds [16], and tryprostatins [17]. These compounds demonstrate a diverse array of pharmacological properties, encompassing antibacterial [1,18], anti-inflammatory [3], antitumor [5], and antioxidant [16] activities. Consequently, they represent promising candidates for the development of novel therapeutic agents.

Protein tyrosine phosphatases (PTPs) function as pivotal “molecular switches” within cellular signal transduction networks. By maintaining a dynamic equilibrium with protein tyrosine kinases, they intricately regulate cell proliferation, differentiation, and metabolic homeostasis. Among the diverse members of this family, specific subtypes such as T-cell protein tyrosine phosphatase (TCPTP), PTP1B, and MEG2 (PTPN9) have been identified as critical regulators implicated in the pathogenesis of major diseases, including diabetes, obesity, and malignancies. In particular, TCPTP has emerged as a compelling therapeutic target for antitumor therapy and immunotherapy due to its unique role in immune homeostasis and oncogenic signaling. However, high structural homology shared among the catalytic domains of the PTP family poses a formidable challenge in developing inhibitors with high selectivity, making this a focal point of current medicinal chemistry research.

Despite the extensive phytochemical exploration of *A. fumigatus*, systematic investigations into the inhibitory effects of its metabolites against the PTP family, particularly regarding selectivity for TCPTP, PTP1B, and MEG2, remain scarce. Therefore, this study aimed to discover structurally unique and highly selective PTP inhibitors by investigating the metabolites of this fungus. We conducted a comprehensive screening campaign targeting a panel of key PTP family members, including TCPTP, PTP1B, MEG2, CD45, SHP1, PTPσ, and LAR. Notably, our screening results revealed that the isolated metabolites exhibited significant inhibitory activity against TCPTP, PTP1B, and MEG2, while showing no significant inhibition on other tested phosphatases (CD45 and SHP1). This finding highlights a unique selectivity profile of *A. fumigatus* metabolites towards these specific phosphatases, offering valuable chemical probes and candidate molecules for the development of targeted therapeutics.

## 2. Materials and Methods

### 2.1. General Experimental Procedure

NMR data and CD spectrum were acquired using a Bruker 600 spectrometer (Bruker, Billerica, MA, USA) and a JASCO J-815 CD spectrometer (JASCO, Tokyo, Japan)). HR-ESIMS data were obtained by Orbitrap Exploris 120 (Thermo Fischer Scientific Inc., Waltham, MA, USA). Reversed-phase HPLC (RP-HPLC) was conducted using an Orienda Brix 1802 LC system equipped with a GRACE Allsphere ODS-2 column (22 × 250 mm, 5 μm). The eluent was monitored by a UV detector at 254 nm. Optical rotation values were determined using an SGW-533 automatic polarimeter (Shanghai Instrument & Electrical, Waltham, MA, USA). Silica gel of CC (Qingdao Haiyang Chemical Group Co. Ltd., Qingdao, China), optical microscope (OLYMPUS CX31), 5180E carbon dioxide incubator (NuAire, Plymouth, MN, USA), full-wavelength microplate reader (TECAN, Männedorf, Switzerland). T-cell protein tyrosine phosphatase (TCPTP), protein tyrosine phosphatase 1B (PTP1B), protein tyrosine phosphatase C receptor (CD45), protein tyrosine phosphatase (SHP1), receptor-type tyrosine phosphatase S (PTP sigma), non-receptor protein tyrosine phosphatase (MEG2), leukocyte common antigen-related phosphatase (LAR), were provided by North China Pharmaceutical Co., Ltd. (Shijiazhuang, China).

### 2.2. Fungal Material

The fungal strain F15ZA56, obtained from the North China Pharmaceutical Group, was grown on PDA at 25 °C. Genomic DNA was extracted, and three loci, including the ITS region, partial *BenA*, and *CaM* genes, were amplified and sequenced. The ITS region was amplified using the primer pair ITS4/ITS5, while *BenA* and *CaM* were amplified using the primer pairs Bt2a/Bt2b and CMD5/CMD6, respectively. The resulting sequences were submitted to NCBI for BLAST(2.15.0) searches. To determine the phylogenetic position, a concatenated dataset of ITS, *BenA*, and *CaM* sequences was constructed. Multiple sequence alignment was performed using MAFFT, and a Maximum Likelihood (ML) phylogenetic tree was inferred using IQ-TREE with 1000 bootstrap replicates. The analysis revealed that F15ZA56 formed a distinct clade with the type strain *Aspergillus fumigatus* NRRL 163. Therefore, combining morphological characteristics with multi-locus sequence analysis, strain F15ZA56 was identified as *Aspergillus fumigatus*. The strain is deposited at the North China Pharmaceutical Group (Shijiazhuang, China) under the accession number F15ZA56.

### 2.3. Fermentation, Extraction and Isolation

*A. fumigatus* F15ZA56 spores were retrieved from a −80 °C freezer and thawed at 45 °C. It was subsequently inoculated into 150 mL conical flasks containing 40 mL of liquid seed medium. A total of 26 flasks were incubated at 200 rpm for 3 days. Subsequently, 10 mL (5% *v*/*v*) of each seed culture was aseptically inoculated into a 1000 mL Erlenmeyer flask containing 100 g of solid rice medium. This resulting in 80 fermentation flasks with a total substrate weight of 8.0 kg. Solid-state fermentation was conducted under static conditions at 26 °C for a duration of 14 days.

The fermented substrate underwent triple extraction with 16 L of 95% ethanol at ambient temperature. Subsequent removal of the solvent from the pooled extracts via rotary evaporation under reduced pressure afforded 120 g of crude extract. This residue was then suspended in 200 mL of distilled water and subjected to sequential liquid–liquid partitioning using petroleum ether, CH_2_Cl_2_, and EtOAc.

The CH_2_Cl_2_ extract (14.2 g) was subjected to medium-pressure silica gel column chromatography. The sample was adsorbed onto 14.2 g of silica gel (100–200 mesh), and the column was packed with 150.0 g of silica gel (300–400 mesh). Gradient elution was performed using a CH_2_Cl_2_-MeOH solvent system (100:0 to 0:100) over 6 h (45 mL/min, 254 nm), yielding 13 fractions (Fr.1–Fr.13). Fr.4 (0.16 g) was purified by HPLC (10%→47% MeCN/H_2_O, 20 mL/min, 254 nm) to afford compound **1** (4.0 mg, t_R_ = 8.5 min). Fr.5 (0.3 g) was subjected to HPLC separation with the following gradient: isocratic elution with 50% MeCN/H_2_O for 10 min, followed by a linear gradient 50%→100% MeCN/H_2_O over 5 min, giving compound **2** (3.9 mg, t_R_ = 10.1 min). The fractions eluted with 100% MeCN were further subjected to HPLC (90% MeCN/H_2_O, 10 min), yielding compound **3** (5.1 mg, t_R_ = 3.8 min) and compound **4** (3.6 mg, t_R_ = 4.6 min).

The EtOAc extract (14.6 g) was subjected to medium-pressure silica gel CC. The sample was adsorbed onto 14.6 g of silica gel (100–200 mesh), and the column was packed with 150.0 g of silica gel (300–400 mesh). Gradient elution was performed using a CH_2_Cl_2_-MeOH solvent system (80:20→0:100, 45 mL/min) over 6 h, yielding 11 fractions (Fr.1–Fr.11). Fr.3 (122 mg) was purified by HPLC (25% MeCN/H_2_O for 15 min, followed by 25%→100% MeCN/H_2_O over 10 min, 4.0 mL/min, 210 nm) to afford compound **5** (3.9 mg, t_R_ = 19.2 min). Fr.6 (0.1 g) was subjected to HPLC separation using isocratic elution with 35% MeCN/H_2_O for 15 min (20 mL/min, 254 nm), yielding compound **6** (12.1 mg, t_R_ = 6.5 min). Fr.9 (0.6 g) was purified by HPLC using isocratic elution with 10% MeCN/H_2_O for 35 min (20 mL/min, 254 nm), affording compound **7** (3.2 mg, t_R_ = 14.3 min), compound **8** (2.9 mg, t_R_ = 14.8 min), compound **9** (8.6 mg, t_R_ = 21.0 min), compound **10** (3.6 mg, t_R_ = 25.2 min), and compound **11** (2.8 mg, t_R_ = 28.9 min). The preparative HPLC chromatograms of compounds **7**–**11** are shown in Figure S1.

Aspergienyne R (**1**): light yellow powder; UV (MeOH, λmax, nm): 266; ${\left[\alpha \right]}_{D}^{20}$ –2.25 (c 0.04, MeOH); NMR data (Table 1); HRESIMS *m*/*z*: 213.0527 [M-Ac-H+Na]^−^ (calcd. C_11_H_10_O_3_Na^−^, 213.0533).

(*R*)-(6-(hydroxymethyl)-2-methyl-2*H*-chromen-2-yl)methyl acetate (**2**): yellow oil; UV (MeOH, λmax, nm): 224, 266, 311; ${\left[\alpha \right]}_{D}^{20}$ −6.8 (c 0.02, MeOH); NMR data (Table 2); HRESIMS *m*/*z*: 249.1110 [M+H]^+^ (calcd. C_14_H_17_O_4_^+^, 249.1121).

(*S*)-(2-(hydroxymethyl)-2-methyl-2*H*-chromen-6-yl)methyl acetate (**3**): yellow oil; UV (MeOH, λmax, nm): 226, 268, 312; ${\left[\alpha \right]}_{D}^{20}$ +2.94 (c 0.03, MeOH); NMR data (Table 2); HRESIMS *m*/*z*: 247.0972 [M-H]^−^ (calcd. C_14_H_15_O_4_^−^, 247.0974).

2,2-dimethyl-2*H*-chromene-6-carboxamide (**4**): yellow oil; UV (MeOH, λmax, nm): 293; NMR data (Table 2); HRESIMS *m*/*z*: 204.1021 [M+H]^+^ (calcd. C_12_H_14_NO_2_^+^, 204.1024).

Aspergienyne S (**5**): yellow oil; UV (MeOH, λmax, nm): 261; NMR data (Table 3); HRESIMS *m*/*z*: 279.1233 [M+H]^+^ (calcd. C_15_H_19_NO_5_^+^, 279.1232).

Aspergienyne T (**6**): yellow powder; UV (MeOH, λmax, nm): 261; NMR data (Table 3); HRESIMS *m*/*z*: 235.0969 [M-H]^−^ (calcd. C_13_H_15_O_4_^−^, 235.0970).

Aspergienyne U (**7**): yellow powder; UV (MeOH, λmax, nm): 259; ${\left[\alpha \right]}_{D}^{20}$ +10.50 (c 0.04, MeOH); NMR data (Table 4); HRESIMS *m*/*z*: 211.0968 [M+H]^+^ (calcd. 211.0965).

Aspergienyne V (**8**): yellow powder; ${\left[\alpha \right]}_{D}^{20}$ +10.0 (c 0.04, MeOH); UV (MeOH, λmax, nm): 256; ^1^H NMR and ^13^C NMR data (Table 4); HRESIMS *m*/*z*: 248.0459 [M-H+K]^−^ (calcd for, 248.0456).

Aspergienyne W (**9**): yellow powder; ${\left[\alpha \right]}_{D}^{20}$ −12.8 (c 0.04, MeOH);UV (MeOH, λmax, nm): 259; ^1^H NMR and ^13^C NMR data (Table 4); HRESIMS *m*/*z*: 248.0463 [M-H+K]^−^ (calcd for, 248.0456).

Aspergienyne X (**10**): yellow powder; ${\left[\alpha \right]}_{D}^{20}$ −11.8 (c 0.04, MeOH);UV (MeOH, λmax, nm): 259; ^1^H NMR and ^13^C NMR data (Table 4); HRESIMS *m*/*z*: 211.0969 [M+H]^+^ (calcd for, 211.0965).

Aspergienyne Y (**11**): brown powder; ${\left[\alpha \right]}_{D}^{20}$ −10.6 (c 0.5, MeOH); UV (MeOH, λmax, nm): 260; ^1^H NMR and ^13^C NMR data (Table 1); HRESIMS *m*/*z*: 193.0860 [M-H]^−^ (calcd for C_11_H_13_O_3_^−^, 193.0865).

### 2.4. ECD and NMR Calculations

The absolute configurations of the new compounds **1**–**2** and **5**–**11** were elucidated by comparing experimental and calculated ECD spectra and performing NMR calculations. The ECD spectra were calculated using the TD-DFT method at the B3LYP/6-311G(d) level in methanol. Detailed procedures follow our prior work [19], and full computational data are provided in the Supporting Information.

### 2.5. PTP Inhibition Assay

The inhibitory activities of **1**–**13** against PTPs were evaluated using pNPP as a substrate. In this assay, pNPP is hydrolyzed by PTPs to yield inorganic phosphate and p-nitrophenol (pNP), which exhibits a strong absorbance peak at 405 nm. Therefore, the inhibitory potency of the compounds was determined by monitoring the decrease in absorbance caused by the inhibition of pNP formation. The assay was performed in 96-well plates, with 1 μL of test compound per well, 50 μL of enzyme solution in buffer (50 mM Tris, 2 mM DTT, and 2 mM EDTA, pH 6.0), and 50 μL of pNPP solution (final concentration 2.5 mM). After incubating the reaction mixture at 37 °C for 30 min, the reaction was terminated, and the absorbance at 405 nm was measured with a microplate reader. The IC_50_ values were determined by plotting the logarithm of the compound concentration against the enzyme activity. The above-mentioned experiments were conducted in triplicate.

### 2.6. Molecular Docking

The protein structure (PDB ID: 1AAX) was retrieved from the RCSB Protein Data Bank (https://www.rcsb.org/). The SMILES string of the small-molecule ligand is O=C(O)C1=CC=C2C(C=CC(C)(C)O2)=C1 (Comp **12**). In this study, a semi-flexible docking protocol was employed to generate stable protein-ligand complexes. Both the protein and the ligand were prepared using AutoDock Tools (ADT) version 1.5.6. During the preparation process, hydrogen atoms were added to the ligand. The torsion tree menu in ADT was utilized to inspect and define the rotatable bonds and torsion angles of the ligand. For the docking simulation, the grid box was centered at coordinates (44.264, 16.730, 15.247) with dimensions of 68 × 54 × 58 Å. All other parameters were kept at their default values.

## 3. Results and Discussion

### 3.1. Phylogenetic Analysis

**Figure 1:**
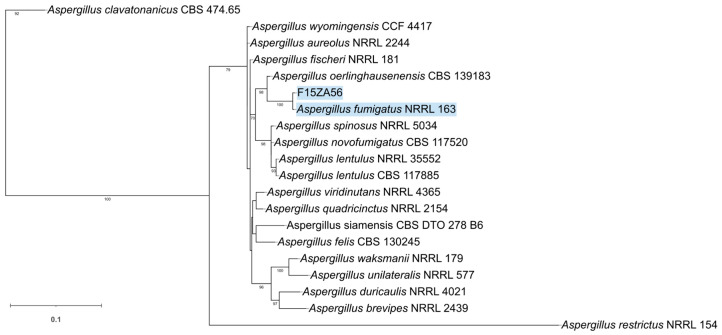
Neighbour-joining tree based on ITS, *BenA*, and *CaM* sequences. Numbers above branches are bootstrap values. Only values above 70% are indicated.

Phylogenetic analysis based on the concatenated ITS, *BenA*, and *CaM* sequences revealed that strain F15ZA56 formed a distinct clade with the reference strain *Aspergillus fumigatus* NRRL 163 with strong statistical support (Bootstrap value = 100%), confirming its identity as *A. fumigatus * (Figure 1).

### 3.2. Structure Determination

**Figure 2:**
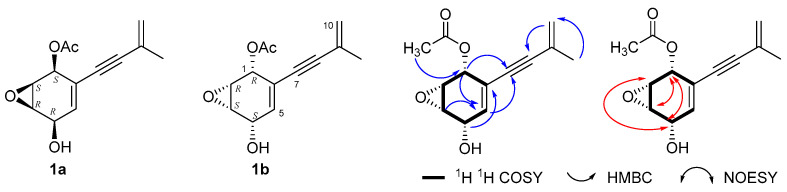
Structures, Key COSY, HMBC, and NOESY correlations in compound **1**.

Compound **1** was purified as a light yellow powder. Its molecular formula was established as C_13_H_14_O_4_ based on HRESIMS (*m*/*z* [M-Ac-H+Na]^−^ 213.0527, calcd. for C_11_H_10_O_3_Na^−^, 213.0533, Figure S2). Compound **1** exhibited 13 carbon resonances in its ^13^C NMR spectrum, including two methyl groups at *δ*_C_ 23.4 (11-CH_3_) and *δ*_C_ 21.1 (1-OCOCH_3_), four oxymethines at *δ*_C_ 67.7, 52.1, 53.6, and 65.6 (C-1 to C-4), and one olefinic carbon at *δ*_C_ 123.4 (C-10). Six quaternary carbons, including two acetylenic carbons (*δ*_C_ 84.4, C-7; *δ*_C_ 93.4, C-8), three sp^2^ carbons (*δ*_C_ 128.8, C-5; *δ*_C_ 123.7, C-6; *δ*_C_ 126.2, C-9), and one carbon of the carbonyl group *δ*_C_ 170.7 (1-OCOCH_3_) were also observed (Table 1). Compound **1** was supposed to be a cyclohexene derivative with a 3-methyl-3-buten-1-yn-1-yl group. The ^1^H NMR spectrum showed four oxymethine protons (*δ*_H_ 5.64, H-1; *δ*_H_ 3.63, H-2; *δ*_H_ 3.64, H-3; *δ*_H_ 4.41, H-4), two olefinic protons at *δ*_H_ 5.30/5.35 (CH_2_-10), one olefinic proton at *δ*_H_ 5.76 (H-5), and a terminal methyl at *δ*_H_ 1.91 (CH_3_-11), which confirmed the above speculation on the structural framework. The ^1^H-^1^H COSY spectrum established the spin connectivity sequence from H-1 through H-5, along with a correlation between H-1 and H-6. The connection of the enyne moiety at C-6 was confirmed by key HMBC correlations from H-5 to C-7 and C-1, as well as from the CH_2_-10/CH_3_-11 protons to C-8 and C-9 (Figures S4–S6). According to the literature [20,21,22,23,24,25], the relative configuration of cyclohexenol derivatives is primarily deduced from coupling constants and confirmed by NOESY correlations. Protons in a *cis*-orientation typically exhibit coupling constants of less than 5.8 Hz, whereas those in a *trans*-orientation display values between 7.0 and 10.5 Hz. Therefore, the relative configuration was first assigned based on these coupling constants, followed by the determination of the absolute configuration using ECD spectroscopy. The coupling constants from H-1 to H-4 are all less than 5.4 Hz, which indicates that their protons are on the same face. The relative configuration was further supported by NOESY interactions connecting H-1 with H-3 and H-4, and H-2 with H-4 (**1a** or **1b**, Figure 2). Based on the calculated ECD data (Figure S3), the absolute configuration was assigned as **1b**, named aspergienyne R.

**Figure 3:**
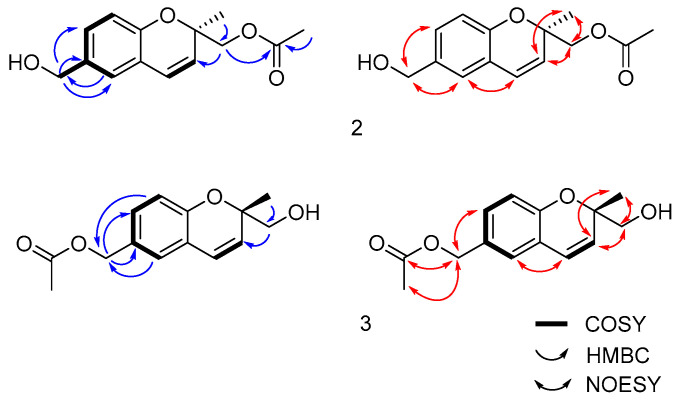
Key COSY, HMBC, and NOESY correlations in **2**–**3**.

Compound **2** is a yellow powder, and its molecular formula was determined to be C_14_H_16_O_4_ based on HRESIMS (*m*/*z* [M+H]^+^ 249.1110, calcd. for C_14_H_17_O_4_^+^, 249.1121, Figure S7). The proton NMR spectrum displayed a doublet of doublets at *δ*_H_ 7.68 (*J* = 8.3, 2.1 Hz), along with two doublets at *δ*_H_ 7.56 (*J* = 2.1 Hz) and *δ*_H_ 6.89 (*J* = 8.3 Hz), characteristic of a 1, 2, 4-substituted benzene ring. The signals, *δ*_H_ 6.57 (d, *J* = 10.0 Hz) and 5.76 (d, *J* = 10.0 Hz), were assigned to a pair of *cis*-substituted alkenyl protons. All above signals indicated that compound **2** possessed a 2, 6-disubstituted benzopyran skeleton (also known as chromene). The presence of an acetyl group was confirmed by NMR signals at *δ*_C_ 172.5/20.6 and *δ*_H_ 1.95 (3H, s). HMBC data located the hydroxymethyl group at C-6 (correlations from *δ*_H_ 4.48 to C-5/C-6/C-7). The methyl (*δ*_H_ 1.41) and acetoxy-methyl (*δ*_H_ 4.18, 4.10) groups were assigned to C-2 of the pyran ring based on correlations to C-2/C-3, with the latter further correlating to the carbonyl C-12 (Figure 3, Figures S9–S11).

**Figure 4:**
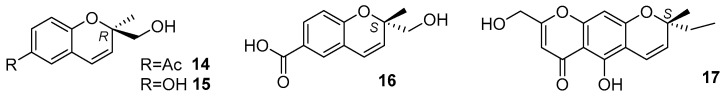
Structures of similar compounds **14**–**17**.

A literature survey revealed that compounds **14** [26] and **15** [27], possessing the 2*R* configuration, exhibited optical rotation values of ${\left[\alpha \right]}_{D}^{20}$20D −4.62 (c 1.6, CHCl_3_) and ${\left[\alpha \right]}_{D}^{20}$ −2.6 (c 0.2, EtOH), respectively (Figure 4). In contrast, compounds **16** [28] and **17** [29], which feature the 2*S* configuration, displayed positive optical rotations of ${\left[\alpha \right]}_{D}^{20}$ +30 (c 0.03, MeOH) and ${\left[\alpha \right]}_{D}^{20}$ +42.3 (c 0.12, MeOH), respectively. Optical rotation data provides a reliable basis for assigning the absolute configuration of C-2. The optical rotation of compound **2** was ${\left[\alpha \right]}_{D}^{20}$ −6.8 (c 0.02, MeOH), suggesting an *R* configuration at C-2. Furthermore, the results of ECD calculations were consistent with this assignment (Figure S8). Compound **2** was named 2*R*-6-hydroxymethyl-2-methyl-2-acetonylchromene.

Compound **3** is a yellow powder, and its molecular formula was established as C_14_H_16_O_4_ based on HRESIMS (*m*/*z* [M-H]^−^ 247.0972, calcd. for C_14_H_15_O_4_^−^, 247.0974, Figure S12). NMR data (Figure S13) indicated that compounds **3** possessed the same benzopyranone framework and substituents as compound **2**. The difference lay in the positions of the hydroxymethyl and acetoxy groups, which were reversed. Correlations of *δ*_H_ 4.98 with *δ*_C_ 124.9, 128.9, 130.7, and 172.8 indicated that this group was attached to the acetyl group and the benzene ring in HMBC spectrum (Figures S14 and S15). The *R* or *S* configuration of C-2 is confirmed by its optical rotation, ${\left[\alpha \right]}_{D}^{20}$ +2.94 (in MeOH; c 0.03), as *S* configuration. Compound **3** was named (*S*)-(2-(hydroxymethyl)-2-methyl-2*H*-chromen-6-yl)methyl acetate.

Compound **4** is a yellow oil, and the molecular formula was determined to be C_12_H_13_NO_2_ based on its HRESIMS (*m*/*z* [M+H]^+^ 204.1021, calcd. for C_12_H_14_NO_2_^+^, 204.1024, Figure S16). The NMR data of this compound resembled those of compounds **2** and **3**, indicating a benzopyranone-type skeleton. At C-2, two methyl groups were observed (*δ*_H_ 1.43, 6H, s), and a substituent was located at C-6, suggested to be a carboxyl or an amide group (Table 2, Figure S17–S19). By comparing the NMR data with those of a compound [30] possessing a carboxyl group at C-6, and considering the HR-ESI-MS data (*m*/*z*: [M+H]^+^ 204.1021), the substituent at C-6 was identified as an amide group. Thus, **4** was elucidated as 2, 2-dimethyl-6-acylaminochromene, a newly discovered natural product.

**Figure 5:**
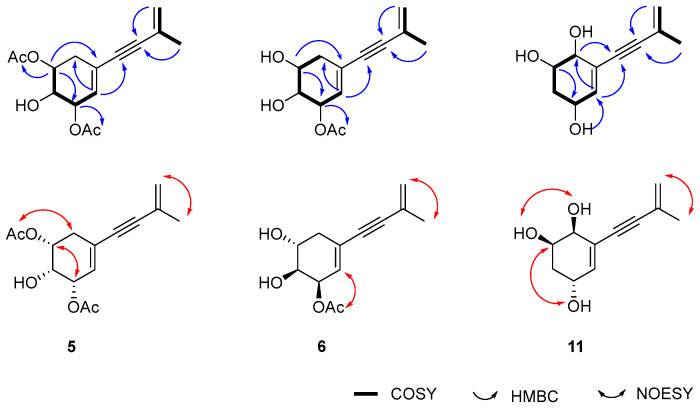
Key COSY, HMBC, and NOESY correlations in **5**–**6**.

Compound **5** is a light yellow powder, and the molecular formula was determined to be C_15_H_18_O_5_ based on HRESIMS (*m*/*z* [M+H]^+^ 279.1233, calcd. for C_15_H_19_O_5_^+^, 279.1232, Figure S20). Its NMR spectrum resembled that of compound **1**, identifying it as a highly oxidized cyclohexene-type compound bearing a 3-methyl-3-buten-1-yn-1-yl substituent at C-6. The structure of **5** was elucidated as 6-(3-methylbut-3-en-1-yn-1-yl)-cyclohex-5-ene-2,4-diacetoxy-3-ol based on spectroscopic evidence. ^1^H-^1^H COSY and HMBC experiments confirmed the substitution of acetoxy groups at C-2 and C-4 and the side chain at C-6. Specifically, HMBC correlations from H-2, H-3, H-4, and H-5 to their respective neighboring carbons, along with side chain correlations (=CH_2_-10/CH_3_-11 to C-6/C-8/C-9/C-11), defined the planar framework (Figure 5).

The relative configuration was inferred from the coupling constants of H-2 (*J* = 5.8 Hz), H-3 (*J* = 5.8 Hz), and H-4 (*J* = 5.4, 4.8, 4.0 Hz), indicating a uniform orientation for the substituents at C-2, C-3, and C-4. The absolute configuration of compound **5** was subsequently assigned using NOESY, DP4+, and ECD analyses (Table 3, Figures S21–S25), and the compound was named aspergienyne S.

Compound **6** was purified as a white powder. Its molecular formula was established as C_13_H_16_O_4_ based on HRESIMS (*m*/*z* [M-H]^−^ 235.0969, calcd. C_13_H_15_O_4_^−^, 235.0970, Figure S26). The data of NMR were very similar to those of **5**. By comparing the molecular formulas of the two, it is found that **6** has one less acetyl group than **5**. Its planar structure was elucidated as 6-(3-methylbut-3-en-1-yn-1-yl)-cyclohex-5-ene-4-acetoxy-2,3-diol based on comprehensive 2D NMR analysis (Figure 5). ^1^H-^1^H COSY correlations delineated the H-1 to H-5 spin system and located the acetoxy group at C-4 via H-4/OAc coupling. The proposed connectivity was supported by HMBC correlations, which showed interactions between H-2/C-3/C-6, H-3/C-2/C-5, H-4/C-2/C-5, and H-5/C-1/C-3/C-7, alongside characteristic long-range correlations from the side-chain protons (=CH_2_-10 and CH_3_-11) to C-8, C-9, C-10, and C-11. The stereostructure is determined by the NOESY spectrum and coupling constants (Table 3, Figures S28–S30). The *J* of H-2 with both H-1s are less than 5.0 Hz, indicating that H-2 occupies a *pseudo*-equatorial position in the boat conformation; The 7.8 Hz coupling constant between H-2 and H-3 suggests a *trans* orientation; and 4.8 Hz between H-3 and H-4 suggests a *cis* orientation. There is no NOE correlation observed between H-2 and H-4, which is consistent with the previous speculation about the orientation of the hydroxyl group. Finally, the absolute configurations of 2*R*, 3*S*, 4*R* were elucidated by ECD calculations (Figure S27). Compound **6** was named aspergienyne T.

**Figure 6:**
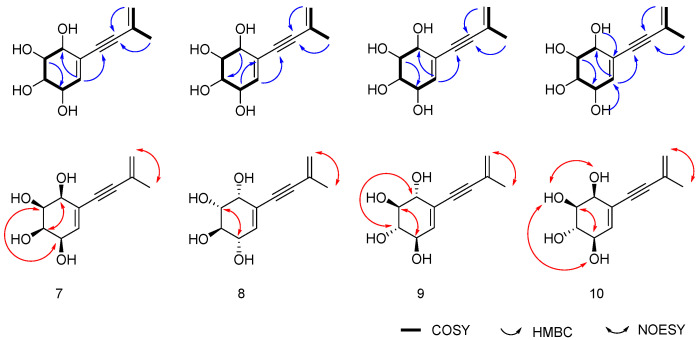
Key COSY, HMBC, and NOESY correlations in **7**–**10**.

Compound **7**, is a white powder, and its molecular formula was determined to be C_11_H_14_O_4_, according to HRESIMS data (*m*/*z* [M+H]^+^ 211.0968, calcd. for C_11_H_15_O_4_^+^, 211.0965, Figure S31). According to the data of NMR (Figures S33–S35), this compound is 6-(3-methylbut-3-en-1-yn-1-yl)-cyclohex-5-ene-1,2,3,4-tetrols (Figure 6). Coupling constants (*J*) of 2.7, 3.0, 3.0, and 2.7 Hz for H-1 through H-4 indicate a *cis* configuration between adjacent protons. The relative configuration was supported by NOESY correlations observed between H-1 and H-3, as well as between H-2 and H-4. These findings confirm the validity of the above hypothesis. Subsequently, ECD calculations were performed to confirm the absolute configuration as 1*S*,2*S*,3*R*,4*R* (Figure S32). Compound **7** was named aspergienyne U.

Compound **8** was purified as a white powder. Its molecular formula, C_11_H_14_O_4_, was determined according to HRESIMS data (*m*/*z* [M-H+K]^−^ 248.0459, calcd. C_11_H_13_O_4_K^−^, 248.0456, Figure S36). It is also 6-(3-methylbut-3-en-1-yn-1-yl)-cyclohex-5-ene-1,2,3,4-tetrols (Figure 6), according to its data of NMR (Figures S38–S40). H-1 and H-2 are in a *cis* configuration, according to the coupling constant (4.8 Hz) of H-2 with H-1. The 10.0 Hz coupling constant between H-2 and H-3 suggests a *trans* orientation; and 7.3 Hz between H-3 and H-4 also suggests a *trans* orientation. The observation of NOESY correlation for H-2 and H-4 corroborated the proposed relative configuration, while ECD calculations confirmed the absolute configuration as 1*R*,2*R*,3*R*,4*S*. Compound **8** was named aspergienyne V.

Compound **9** was isolaed as a white powder. Its molecular formula, C_11_H_14_O_4_, was determined based on HRESIMS (*m*/*z* [M-H+K]^−^ 248.0463, calcd. C_11_H_13_O_4_K^−^, 248.0456, Figure S41). Similar to the compound described above, Compound **9** was also 6-(3-methylbut-3-en-1-yn-1-yl)-cyclohex-5-ene-1,2,3,4-tetrols (Figure 6), according to its data of NMR (Figures S43–S45). Coupling constants (*J*) of 7.2, 10,2, 10.2, and 7.4 Hz for H-1 through H-4 indicate a *trans* configuration between adjacent protons. Correlations between H-1/H-3, and H-2/H-4, were observed in the NOESY spectrum, confirming the validity of the above hypothesis. Subsequently, ECD calculations were performed to determine the absolute configuration as 1*R*,2*S*,3*S*,4*R* (Figure S42). Compound **9** was named aspergienyne W.

Compound **10** is a white powder, and its molecular formula was determined to be C_11_H_14_O_4_ based on its HRESIMS (*m*/*z* [M+H]^+^ 211.0969, calcd. C_11_H_15_O_4_^+^, 211.0965, Figure S46). The relative configuration was deduced to be the same as that of compound **8**, according to the coupling constants. However, ECD calculations identified it as the enantiomer of **8**, named aspergienyne X (Figures S47–S50).

Interestingly, while aspergienyne Q of this type has been previously reported from a mangrove endophytic fungal strain [22], our investigation into *A. fumigatus* has yielded four distinct stereoisomers.

Compound **11** was purified as a yellow powder, and its molecular formula was established as C_11_H_14_O_3_ based on HRESIMS data (*m*/*z* [M-H]^−^ 193.0860, calcd. C_11_H_13_O_3_^−^, 193.0865, Figure S51). From the NMR data, it can be seen that, similar to the previous compounds, this is also a cyclohexenol compound with a 6-(3-Methylbut-3-en-1-yn-1-yl) substitution. However, it lacks one carbon atom with an oxygen substitution. A carbon signal (*δ*_C_ 34.4) appears in the high-field region, indicating that only three hydroxyl groups substituted on the cyclohexene ring. The HMQC spectrum shows two sets of high-field hydrogen signals (*δ*_H_ 1.66 and 1.73) connected to *δ*_C_ 34.4, confirming the above speculation. ^1^H-^1^H COSY correlations confirmed the connectivity of the H-1 to H-5 chain, and H-10/H-11 fragment. The presence of hydroxyl substituents was identified at C-1, C-2, and C-4, based on correlations with protons at *δ*_H_ 5.23, 4.81, and 4.83, respectively. The HMBC spectrum provided long-range correlations (H-1 to C-5, 6, 7; H-2 to C-4, 6; H-3 to C-2, 5; H-4 to C-5, 6; H-5 to C-1, 7) that defined the core skeleton, while correlations from H-10 and CH_3_-11 confirmed the connectivity of the side chain. This further determines the attribution of each hydrogen signal and carbon signal. In summary, the planar structure of the compound is 6-(3-methylbut-3-en-1-yn-1-yl)-cyclohex-5-ene-1,2,4-triol. In NOESY spectrum, the hydrogen signals of 1-OH and 2-OH are correlated, indicating that their orientations are the same (Figures S53–S55). Also, H-2 and 4-OH are correlated, indicating that their orientations are the same. Thus, the relative stereostructure of **11** is determined (Figure 5). The absolute configurations of these three chiral carbons, 1S, 2R, 4R, were determined through ECD calculations (Figure S52). Compound **11** was named aspergienynes Y.

In addition, two known compounds were identified as anofinoc acid (**12**) [30] and asperpentyn (**13**) [31].

### 3.3. PTPs Inhibitory Activities of Isolated Compounds

All compounds were tested for their inhibitory activities against a panel of seven protein tyrosine phosphatases (PTPs), including TCPTP, PTP1B, MEG2, CD45, SHP1, PTPσ, and LAR. Among them, compounds **4**, **12**, and **13** exhibited varying degrees of inhibitory potency against the first three enzymes (TCPTP, PTP1B, and MEG2). Specifically, compound **4** showed significant inhibition against TCPTP, PTP1B, and MEG2, with IC_50_ values of 43.22 ± 3.39 nM, 20.16 ± 2.01 nM, and 62.19 ± 5.83 nM, respectively. Compound **12** displayed potent activity exclusively against PTP1B, with an IC_50_ value of 12.20±1.23 nM. Compound **13** demonstrated significant inhibition against TCPTP and PTP1B, with IC_50_ values of 26.60 ± 2.44 nM and 21.56 ± 2.06 nM, respectively. The positive control, AC484, exhibited IC_50_ values of 12.7 ± 0.48 nM, 9.12 ± 0.38 nM, and 20.1 ± 0.52 nM against TCPTP, PTP1B, and MEG2, respectively, Figures S56–S58.

### 3.4. Molecular Docking

**Figure 7:**
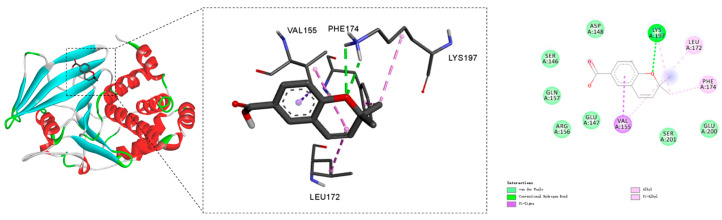
Molecular docking results of compound **12** with PTP1B.

Molecular docking of compound **12** was performed using AutoDock Vina(v1.1.2). The top 10 binding conformations were evaluated and ranked based on their binding affinities (Table S1). The conformation with the most favorable binding energy was selected for further interaction analysis (Figure 7). The ligand is stabilized within the binding pocket through multiple non-covalent interactions, including hydrogen bonds, Pi-Sigma, alkyl, and Pi-alkyl interactions. Specifically, a hydrogen bond is formed with Lys197, a Pi-Sigma interaction with Val155, and alkyl as well as Pi-alkyl interactions with Leu172 and Phe174.

## 4. Conclusions

**Figure 8:**
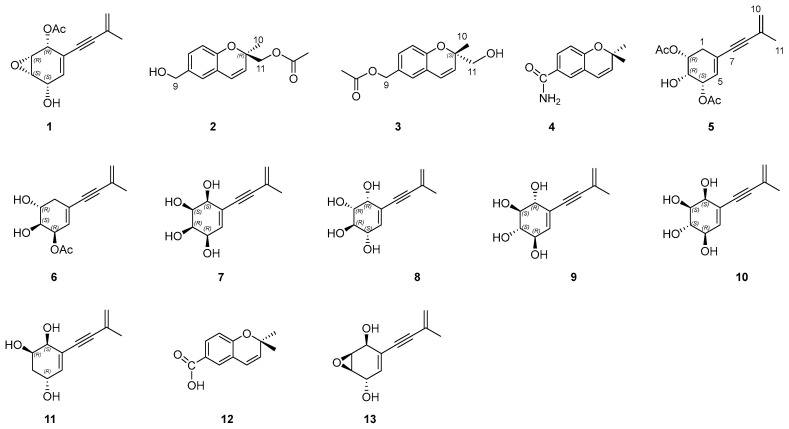
Structures of compounds **1**–**13**. Molecular docking results of compound 12 with PTP1B.

A total of 13 compounds were found from *A. fumigatus* F15ZA56, and compounds **1**–**11** were newly isolated from nature for the first time (Figure 8). The biosynthetic pathways for compounds **1** and **5**–**11** were hypothesized to involve enzyme-mediated reactions resulting in diverse stereoisomers, Figure S59. Compounds **2**–**4** are benzopyran derivatives, and compounds **1**, **5**–**11** are highly oxygenated cyclohexenol derivatives. In particular, the structural elucidation of these cyclohexenols provides a strategy and data support for determining the absolute configurations of this class of compounds.

Bioassay results identified compounds **4**, **12**, and **13** as potent PTP inhibitors. Notably, compound **4** acted as a dual/multi-target inhibitor with nanomolar potency against TCPTP, PTP1B, and MEG2. In contrast, compound **12** exhibited remarkable selectivity towards PTP1B over the other six PTPs, including the highly homologous TCPTP, with an IC_50_ value comparable to the positive control AC484. The molecular docking study confirms that **12** possesses a strong binding potential towards PTP1B. The formation of a critical hydrogen bond with Lys197, supported by hydrophobic contacts with Val155, Leu172, and Phe174, contributes significantly to the stability of the complex. These structural insights highlight the **12** as a promising candidate for PTP1B inhibition. These findings suggest that subtle structural variations within this scaffold significantly influence enzyme selectivity, providing a valuable basis for the future design of selective PTP1B inhibitors.

## Figures and Tables

**Table 1:** ^1^H (600 MHz) and ^13^C (150 MHz) NMR data of **1** and **11**.

Position	1 (CDCl_3_)		11 (DMSO-*d*_6_)	
	*δ*_C_	*δ*_H_ (mult, *J* in Hz)	*δ*_C_	*δ*_H_ (mult, *J* in Hz)
1	67.7	5.64 (d, 2.5)	70.6	3.61 (brd, 4.4)
2	52.1	3.63 (d, 2.4)	68.8	3.69 (p, 3.2)
3	53.6	3.64 (d, 2.4)	34.4	1.66 (d, 7.4), 1.73 (m)
4	65.6	4.41 (d, 5.4)	62.6	4.17 (t, 8.2)
5	128.8	5.76 (d, 1.6)	139.6	5.97 (d, 3.2)
6	123.7		122.9	
7	84.4		89.1	
8	93.4		89.4	
9	126.2		126.6	
10	123.4	5.30 (p, 1.6) 5.35 (dd, 2.0, 1.0)	121.8	5.25 (m), 5.29 (m)
11	23.4	1.91 ((t, 1.3)	23.3	1.86 (s)
1-OAc	170.7, 21.1	2.16 (s)		
1-OH				5.23 (brs)
2-OH				4.81 (brs)
4-OH		2.25 (d, 7.7)		4.83 (brs)

**Table 2:** ^1^H (600 MHz) and ^13^C (150 MHz) NMR data of **2**–**4** in CD_3_OD.

Position	2		3		4	
	*δ*_C_	*δ*_H_ (mult, *J* in Hz)	*δ*_C_	*δ*_H_ (mult, *J* in Hz)	*δ*_C_	*δ*_H_ (mult, *J* in Hz)
1						
2	78.4		80.5		78.5	
3	129.5	5.63 (d, 9.9)	129.9	5.67 (d, 9.9)	132.4	5.75 (d, 9.9)
4	122.0	6.51 (d, 9.9)	122.5	6.46 (d, 9.9)	122.1	6.41 (d, 9.9)
4a	126.7		128.0		122.6	
5	125.8	7.01 (d, 2.2)	124.9	7.01 (d, 2.2)	129.3	7.67 (d, 2.1)
6	135.4		130.7		130.8	
7	127.3	7.10 (dd, 8.2, 2.2)	128.9	7.10 (dd, 8.2, 2.2)	132.3	7.77 (dd, 8.4, 2.1)
8	116.8	6.69 (d, 8.2)	117.1	6.74 (d, 8.2)	117.0	6.76 (d, 8.4)
8a	153.4		154.3		158.4	
9	64.8	4.48 (s)	67.2	4.98 (s)	168.8	
10	23.8	1.41(s)	20.9	1.35 (s)	28.5	1.43 (s)
11	69.5	4.10 (d, 11.6) 4.18 (d, 11.6)	68.6	3.60 (d, 11.6) 3.54 (d, 11.6)	28.5	1.43 (s)
OAc	172.5 20.6	1.95 (s)	172.8 23.2	2.04 (s)		

**Table 3:** ^1^H (600 MHz) and ^13^C (150 MHz) NMR data of **5**–**6**.

Position	5 (CD_3_OD)		6 (CDCl_3_)	
	*δ*_C_	*δ*_H_ (mult, *J* in Hz)	*δ*_C_	*δ*_H_ (mult, *J* in Hz)
1	30.2	1.30 (dd, 12.0, 4.0) 2.05 (dd, 12.0, 5.6)	33.3	2.11 (dt, 15.0, 3.6) 1.88 (ddd, 15.0, 4.8, 4.8)
2	73.1	5.05 (q, 5.8)	68.8	3.94 (dd, 7.8, 3.6)
3	69.8	4.01 (dt, 5.8, 1.2)	73.5	3.97 (dd, 7.8, 4.8)
4	68.0	5.39 (dd, 5.4, 4.0)	66.8	5.39 (q, 4.5)
5	133.5	6.09 (d, 4.0)	131.0	6.11 (d, 4.8)
6	128.1		128.1	
7	87.4		84.2	
8	92.9		94.4	
9	128.3		126.1	
10	122.9	5.32 (m), 5.30 (m)	123.5	5.37 (m) 5.32 (m)
11	23.4	1.91 (t, 1.3)	23.4	1.93 (3H, t, 1.3)
2-OAc	172.1, 20.9	2.06 (s)		
4-OAc	172.0, 20.9	2.07 (s)	170.4, 21.2	2.05 (s)

**Table 4:** ^1^H (600 MHz) and ^13^C (150 MHz) NMR data of **7**–**10**.

Position	7 (CD_3_OD)		8 (CD_3_OD)		9 (DMSO-*d*_6_)		10 (CD_3_OD)	
	*δ*_C_	*δ*_H_ (mult, *J* in Hz)	*δ*_C_	*δ*_H_ (mult, *J* in Hz)	*δ*_C_	*δ*_H_ (mult, *J* in Hz)	*δ*_C_	*δ*_H_ (mult, *J* in Hz)
1	70.1	4.20 (d, 2.7)	72.0	4.24 (t, 4.8)	71.2	3.91 (dt, 7.2, 2.8)	71.3	4.13 (d, 4.1)
2	70.4	3.91 (d, 3.0)	73.3	3.51 (dd, 10.0, 4.3)	75.3	3.16 (dd, 10.2, 7.2)	72.4	3.42 (dd, 10.5, 4.1)
3	70.8	3.90 (d, 3.0)	74.3	3.72 (dd, 10.0, 7.3)	75.5	3.17 (dd, 10.2, 7.2)	73.3	3.63 (dd, 10.5, 7.7)
4	67.4	4.30 (dd, 4.3, 2.7)	67.5	3.87 (dd, 7.3, 1.8)	72.4	3.78 (dt, 7.4, 2.3)	73.6	3.99 (dd, 7.7, 2.6)
5	136.2	6.03 (d, 4.3)	134.0	6.07 (dd, 5.2, 1.8)	137.3	5.74 (t, 2.2)	138.7	5.95 (d, 2.6)
6	125.9		128.2		123.8		124.0	
7	88.6		87.4		87.8		88.5	
8	91.6		92.6		90.2		91.2	
9	128.3		128.3		126.5		128.2	
10	122.4	5.27 (m), 5.28 (m)	122.6	5.28 (m), 5.30 (m)	122.0	5.25 (m), 5.30 (m)	122.4	5.28 (m), 5.30 (m)
11	23.5	1.90 (t, 1.3)	23.5	1.91 (t, 1.3)	23.3	1.86 (t, 1.3)	23.5	1.90 (t, 1.3)
1-OH						5.08		
2-OH						4.96		
3-OH								
4-OH						5.22		

## Data Availability

The original contributions presented in the study are included in the article/Supplementary Material, further inquiries can be directed to the corresponding authors.

## Supplementary Materials

The following supporting information can be downloaded at: https://www.mdpi.com/article/10.3390/jof12070504/s1.
