# Supplementary material for: New Cyclohexenols and Benzopyran Derivatives from Fungus Aspergillus fumigatus F15ZA56
Source: J Fungi (Basel). 2026 Jul 9;12(7):504. doi: 10.3390/jof12070504 (PMC13413059; doi:10.3390/jof12070504)
Supplement: Supplementary file 1 [file jof-12-00504-s001.zip › jof-4421490-supplementary.pdf]

SUPPLEMENTARY MATERIAL

New Cyclohexenols and Benzopyran Derivatives from Fungus *Aspergillus fumigatus* F15ZA56

Ningning Shi [1], Junling Guo [1], Zhen Zhang [1], Feng Jing [1], Shuoyu Zhao [1], Yan Fu [2], Xinhua Lu [3], Yucheng Gu [4], Binliang Tong [5,*] and Manli Zhang [1,*]

[1] Hebei Key Laboratory of Innovative Drug Development and Evaluation, School of Pharmaceutical Sciences, Hebei Medical University, Shijiazhuang 050017, China; 18800970@hebmu.edu.cn (N.S.); 24033100172@stu.hebmu.edu.cn (J.G.); 13503225760@163.com (Z.Z.); jing4389@126.com (F.J.); 25034100681@stu.hebmu.edu.cn (S.Z.)

[2] Core Facilities and Centers, Hebei Medical University, Shijiazhuang 050017, China; fuyan0228@hebmu.edu.cn

[3] New Drug Research & Development Center of North China Pharmaceutical Group Corporation, Shijiazhuang 052165, China; luxinhua89@yeah.net

[4] Syngenta Jealott's Hill International Reasearch Centre, Bracknell RG12 6EY, Berkshire, UK; yucheng.gu@syngenta.com

[5] Department of Pharmacy, The First Affiliated Hospital of Hebei Medical University, Shijiazhuang 050000, China

* Correspondence: 58201302@hebmu.edu.cn (B.T.); 17800938@hebmu.edu.cn (M.Z.)

Content

Experimental Section 4
Figure S1. Isolation of compounds **7-11** by preparative HPLC 4
Figure S2. HRESIMS spectrum of **1** 5
Figure S3. Structures and experimental ECD spectra of **1** 5
Figure S4. ^{1}H NMR (600 MHz) and ^{13}C NMR (150 MHz) spectra of **1** in $CDCl_3$ 6
Figure S5. ^{1}H-^{1}H COSY and HMBC spectra (600 MHz, $CDCl_3$) of **1** 7
Figure S6. HMQC and NOESY spectra (600 MHz, $CDCl_3$) of **1** 8
Figure S7. HRESIMS spectrum of **2** 9
Figure S8. Experimental ECD spectra of **2** 9
Figure S9. ^{1}H (600 MHz) and ^{13}C (150 MHz) NMR data of **2** in CD_3OD 10
Figure S10. ^{1}H ^{1}H COSY and HMBC spectra (600 MHz, CD_3OD) of **2** 11
Figure S11. HMQC and NOESY spectra (600 MHz, CD_3OD) of **2** 12
Figure S12. HRESIMS spectrum of **3** 13
Figure S13. ^{1}H (600 MHz) and ^{13}C (150 MHz) NMR data of **3** in CD_3OD 14
Figure S14. ^{1}H-^{1}H COSY and HMBC spectra (600 MHz, CD_3OD) of **3** 15
Figure S15. HMQC and NOESY spectra (600 MHz, CD_3OD) of **3** 16
Figure S16. HRESIMS spectrum of **4** 17
Figure S17. ^{1}H (600 MHz) and ^{13}C (150 MHz) NMR data of **4** in CD_3OD 18
Figure S18. ^{1}H-^{1}H COSY and HMBC spectra (600 MHz, CD_3OD) of **4** 19
Figure S19. HMQC and NOESY spectra (600 MHz, CD_3OD) of **4** 20
Figure S20. HRESIMS spectrum of **5** 21
Figure S21. Structures and experimental ECD spectra of **5** 21
Figure S22. ^{1}H (600 MHz) and ^{13}C (150 MHz) NMR data of **5** in CD_3OD 22
Figure S23. ^{1}H-^{1}H COSY and HMBC spectra (600 MHz, CD_3OD) of **5** 23
Figure S24. HMQC and NOESY spectra (600 MHz, CD_3OD) of **5** 24
Figure S25. Regression analyses of experimental versus calculated 13C NMR chemical shifts of model 5a and 5b 25
Figure S26. HRESIMS spectrum of **6** 26
Figure S27. Structures and experimental ECD spectra of **6** 26
Figure S28. ^{1}H (600 MHz) and ^{13}C (150 MHz) NMR data of **6** in $CDCl_3$ 27
Figure S29. ^{1}H-^{1}H COSY and HMBC spectra (600 MHz, $CDCl_3$) of **6** 28
Figure S30. HMQC and NOESY spectra (600 MHz, $CDCl_3$) of **6** 29
Figure S31. HRESIMS spectrum of **7** 30
Figure S32. Structures and experimental ECD spectra of **7** 30
Figure S33. ^{1}H (600 MHz) and ^{13}C (150 MHz) NMR data of **7** in CD_3OD 31
Figure S34. ^{1}H-^{1}H COSY and HMBC spectra (600 MHz, CD_3OD) of **7** 32
Figure S35. HMQC and NOESY spectra (600 MHz, CD_3OD) of **7** 33
Figure S36. HRESIMS spectrum of **8** 34
Figure S37. Structures and experimental ECD spectra of **8** 34
Figure S38. ^{1}H (600 MHz) and ^{13}C (150 MHz) NMR data of **8** in CD_3OD 35
Figure S39. ^{1}H-^{1}H COSY and HMBC spectra (600 MHz, CD_3OD) of **8** 36

Figure S40. HMQC and NOESY spectra (600 MHz, CD_3OD) of **8** 37
Figure S41. HRESIMS spectrum of **9** 38
Figure S42. Structures and experimental ECD spectra of **9** 38
Figure S43. ^1H (600 MHz) and ^{13}C (150 MHz) NMR data of **9** in DMSO-d_6 39
Figure S44. ^1H-^1H COSY and HMBC spectra (600 MHz, DMSO-d_6) of **9** 40
Figure S45. HMQC and NOESY spectra (600 MHz, DMSO-d_6) of **9** 41
Figure S46. HRESIMS spectrum of **10** 42
Figure S47. Structures and experimental ECD spectra of **10** 42
Figure S48. ^1H (600 MHz) and ^{13}C (150 MHz) NMR data of **10** in CD_3OD 43
Figure S49. ^1H-^1H COSY and HMBC spectra (600 MHz, CD_3OD) of **10** 44
Figure S50. HMQC and NOESY spectra (600 MHz, CD_3OD) of **10** 45
Figure S51. HRESIMS spectrum of **11** 46
Figure S52. Structures and experimental ECD spectra of **11** 46
Figure S53. ^1H (600 MHz) and ^{13}C (150 MHz) NMR data of **11** in DMSO-d_6 47
Figure S54. ^1H-^1H COSY and HMBC spectra (600 MHz, DMSO-d_6) of **11** 48
Figure S55. HMQC and NOESY spectra (600 MHz, DMSO-d_6) of **11** 49
Figure S56. The IC_{50} curves of compounds **4**, **13** and AC484 against TCPTP 50
Figure S57. The IC_{50} curves of compounds **4**, **12**, **13** and AC484 against PTP1B 50
Figure S58. The IC_{50} curves of compounds **4** and AC484 against MEG2 50
Figure S59. Possible biosynthetic pathway for compounds **1** and **5-11** 51
Table S1. Binding energies of the top 10 conformations 51

Experimental Section

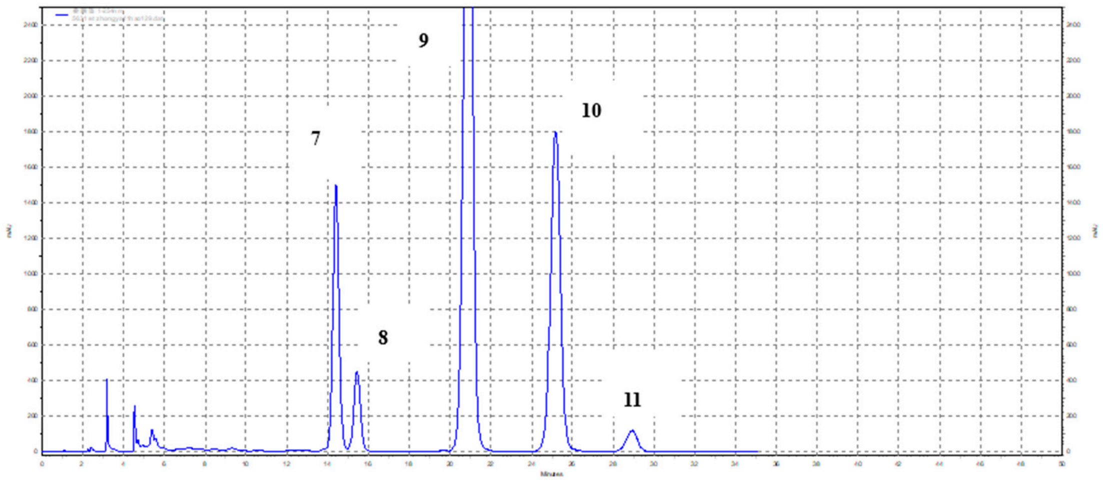

Figure S1. Isolation of compounds **7-11** by preparative HPLC (10% acetonitrile gradient elution for 35 min, 20 mL/min, 254 nm)

ECD calculations were performed based on the deduced configurations. Conformers with the lowest energy were optimized at the B3LYP/6-31G (d) level, and their stability was confirmed by considering harmonic vibrational frequencies. Time-dependent density functional methods were then applied to calculate the electronic transitions of the conformers. The overall theoretical ECD spectra were simulated using a Gaussian function, taking into account Boltzmann weighting of each conformer. The ^{13}C NMR chemical shift calculations were calculated at the B3LYP/6-311+G(d,p) level. The calculated results were averaged using Boltzmann-weighted populations.

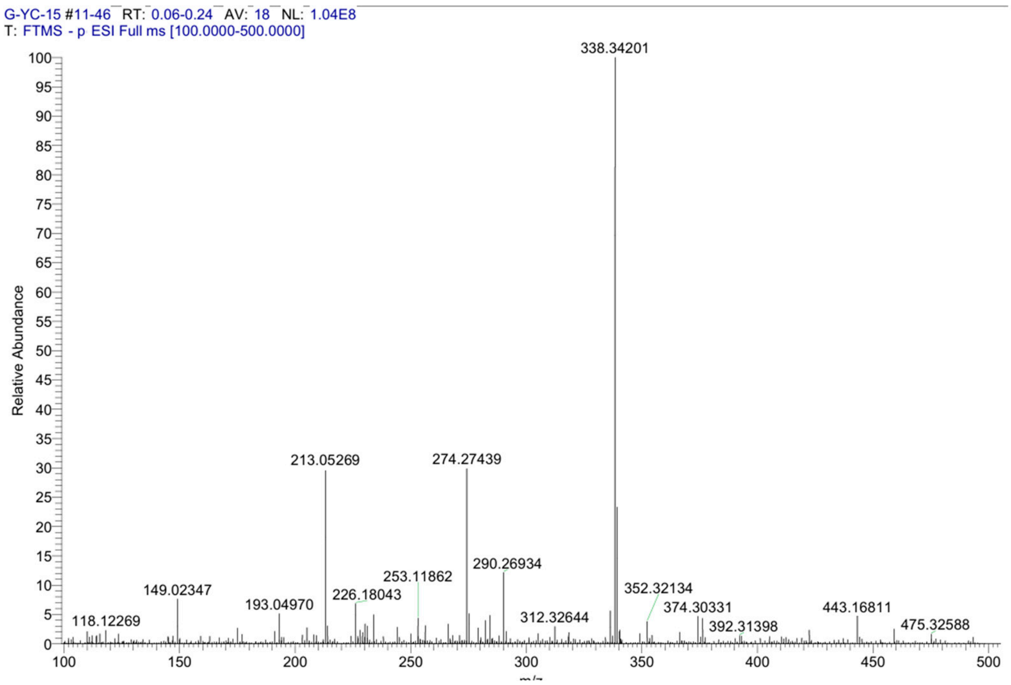

Figure S2. HRESIMS spectrum of **1**

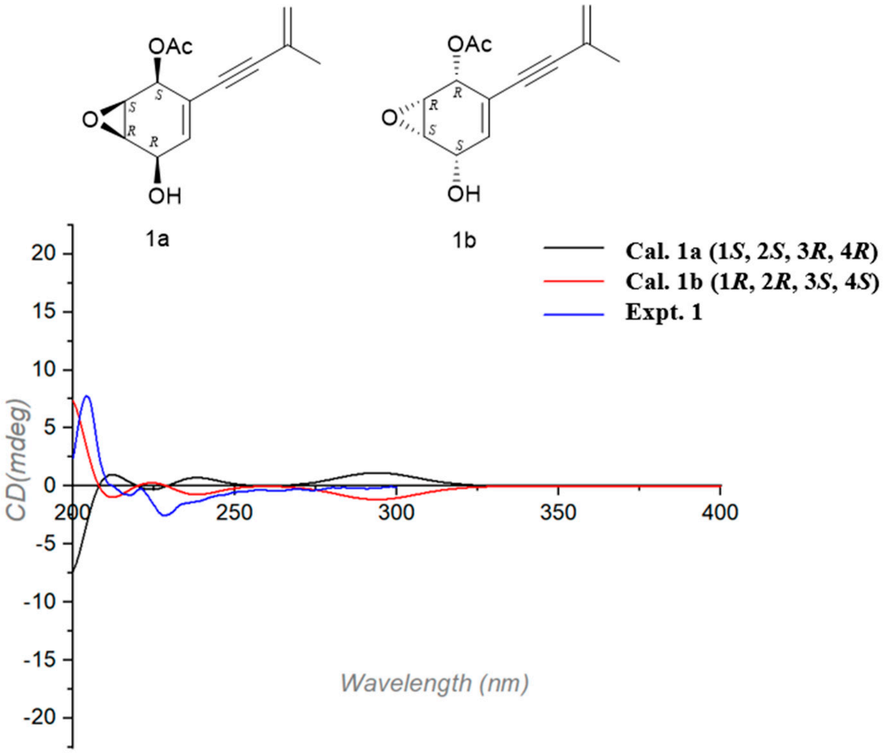

Figure S3. Structures and experimental ECD spectra of **1**

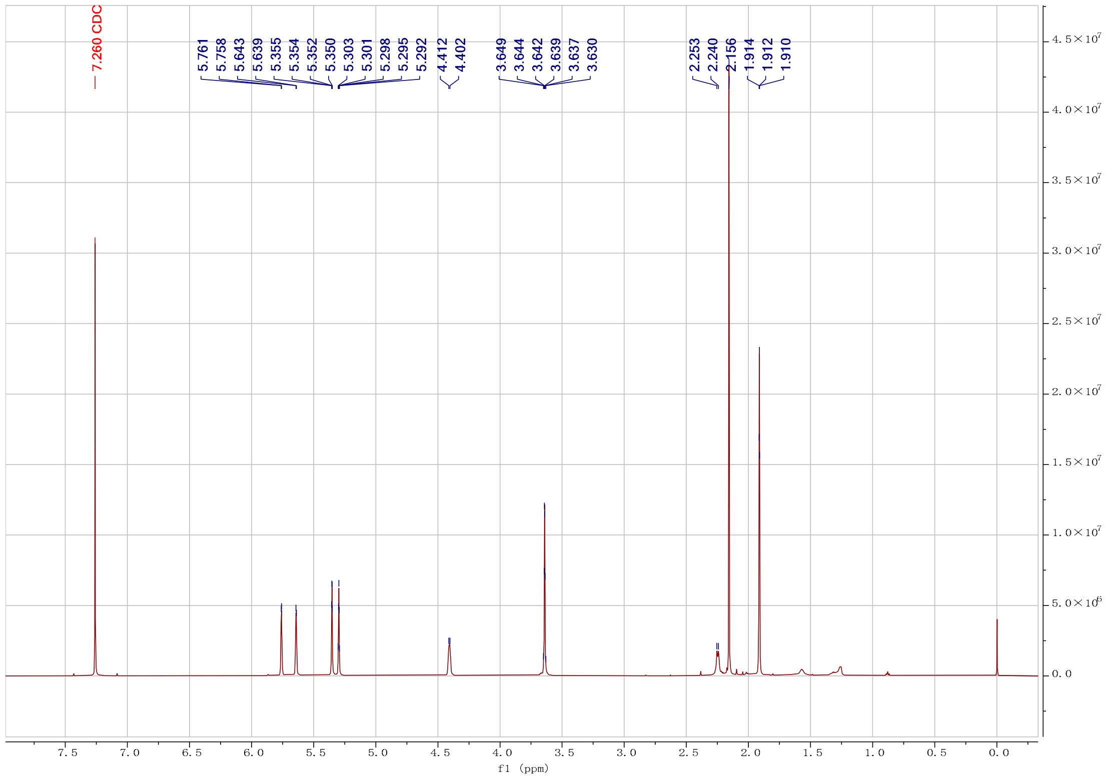

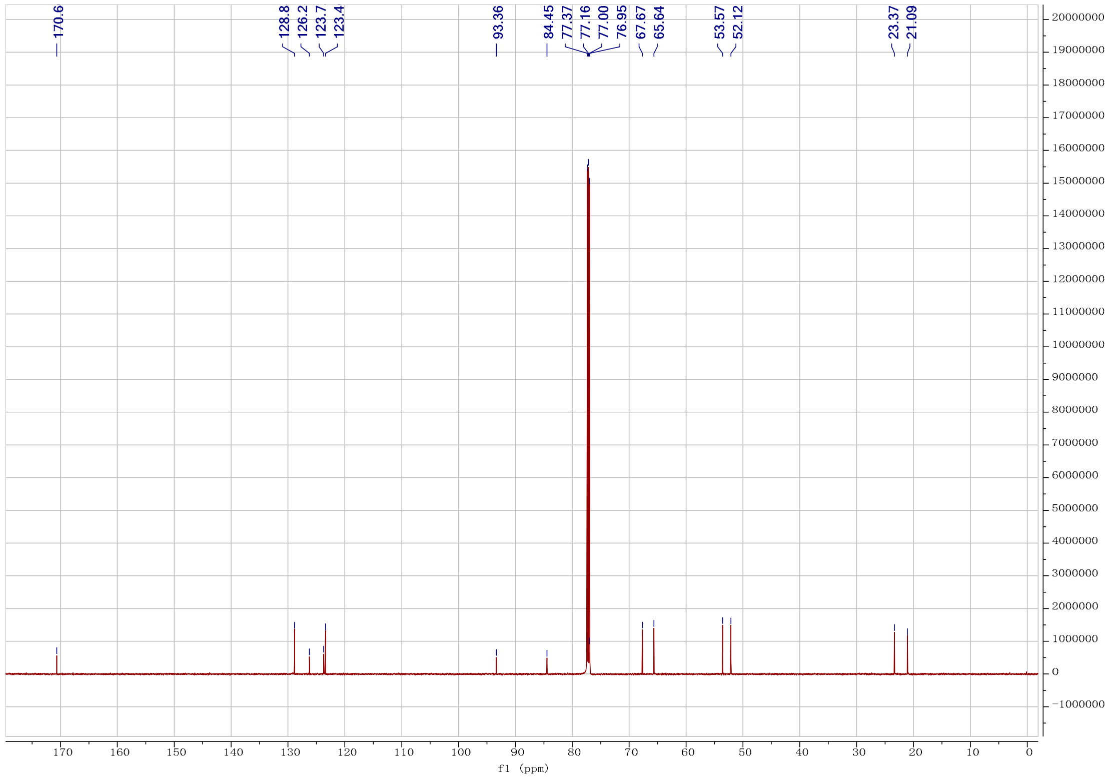

Figure S4. ^{1}H NMR (600 MHz) and ^{13}C NMR (150 MHz) spectra of **1** in $CDCl_3$

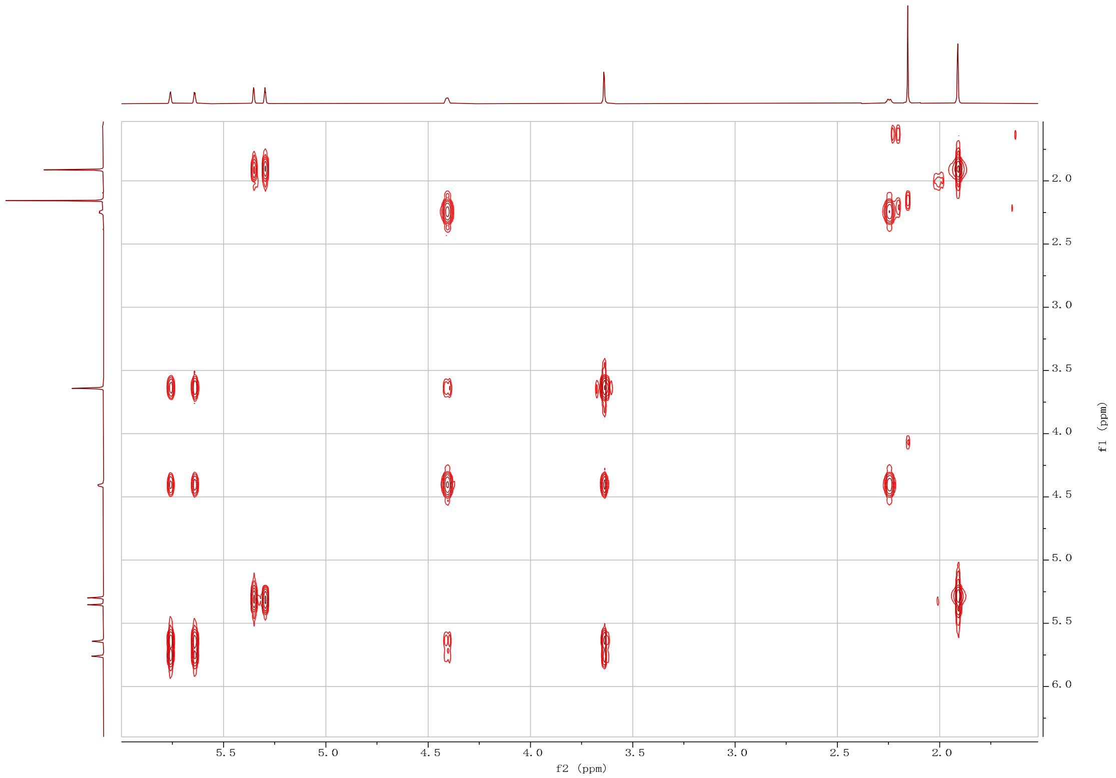

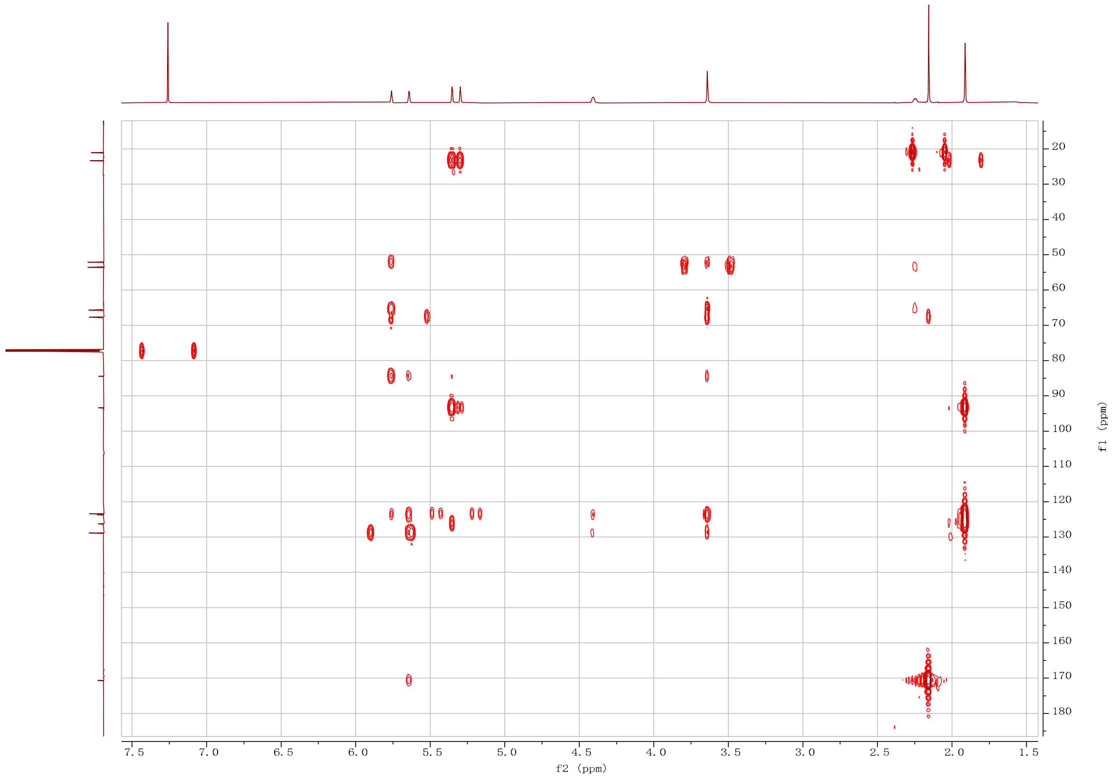

Figure S5. ^{1}H ^{1}H COSY and HMBC spectra (600 MHz, $CDCl_3$) of **1**

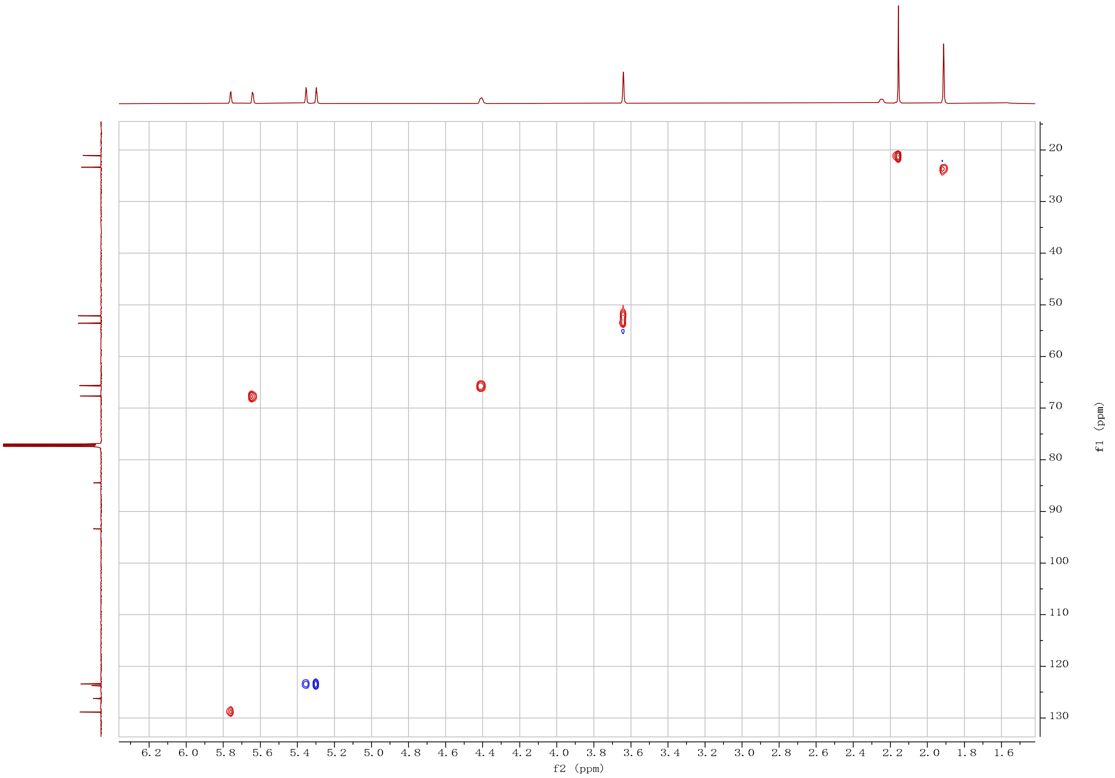

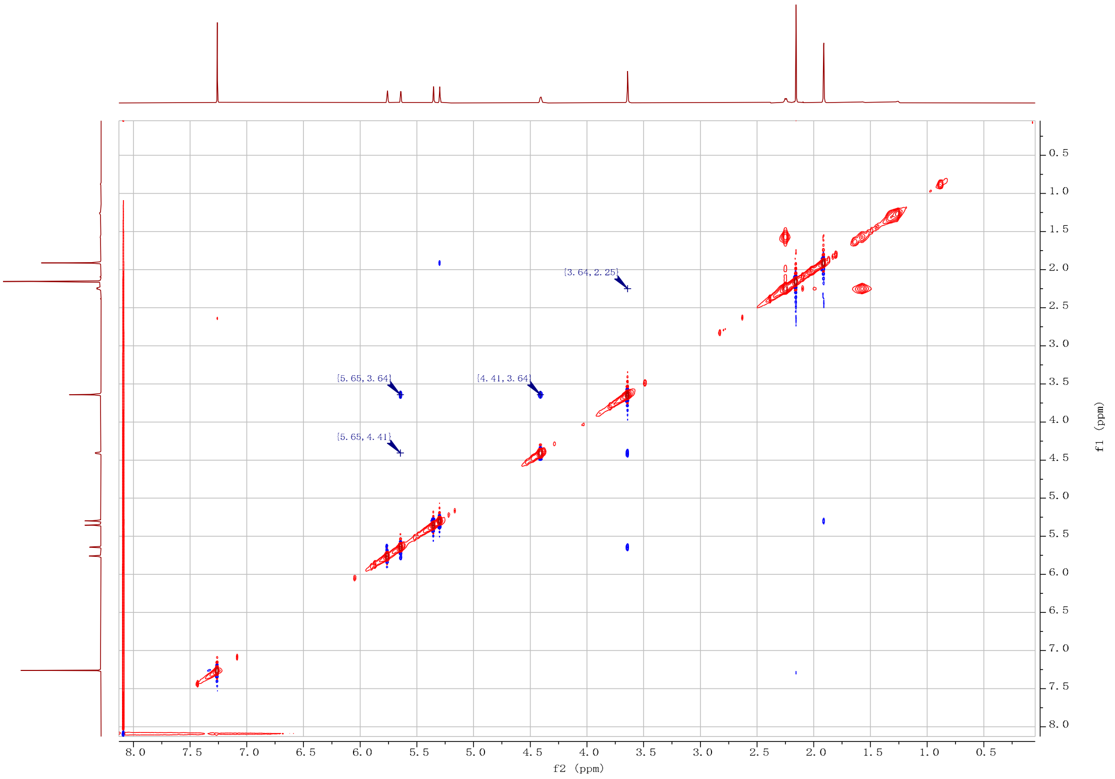

Figure S6. HMQC and NOESY spectra (600 MHz, $CDCl_3$) of **1**

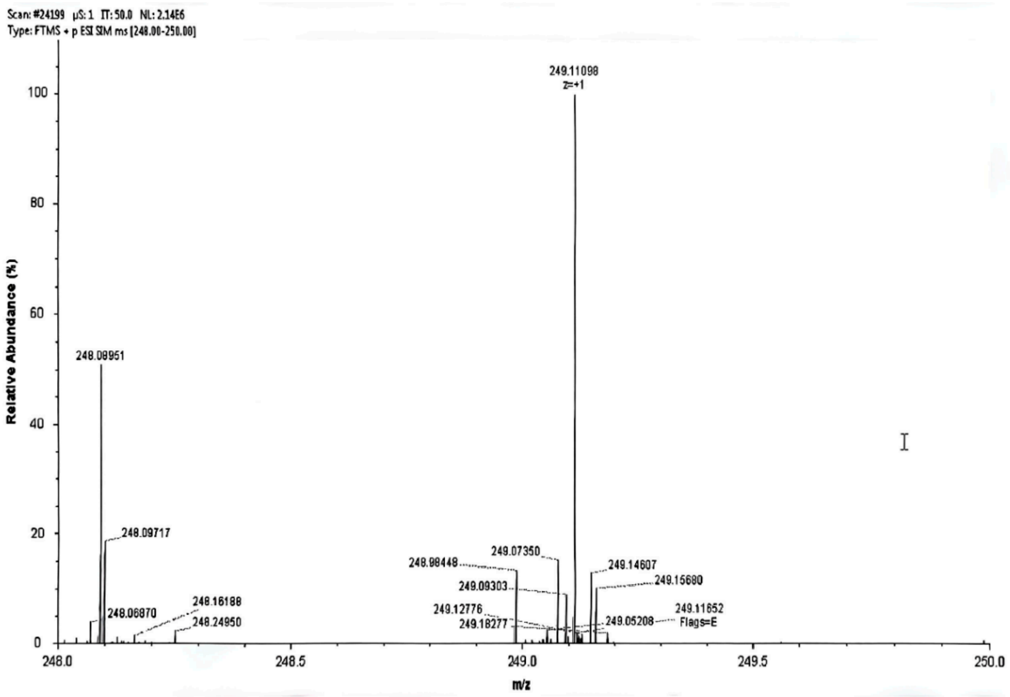

Figure S7. HRESIMS spectrum of **2**

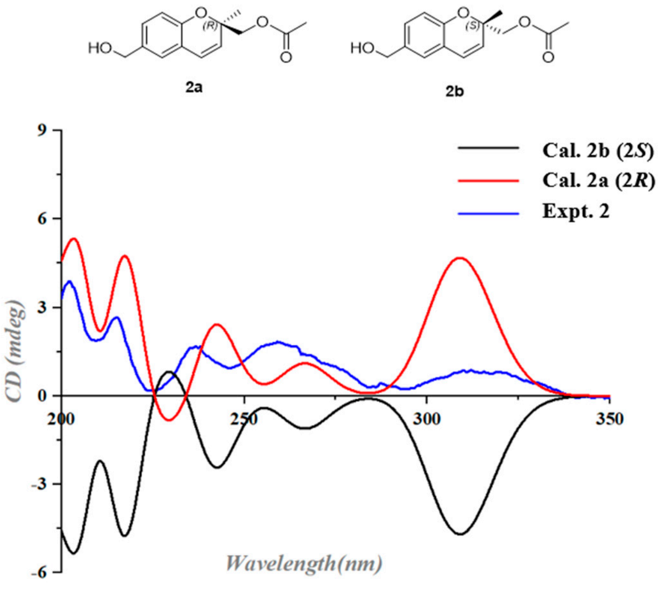

Figure S8. Experimental ECD spectra of **2**

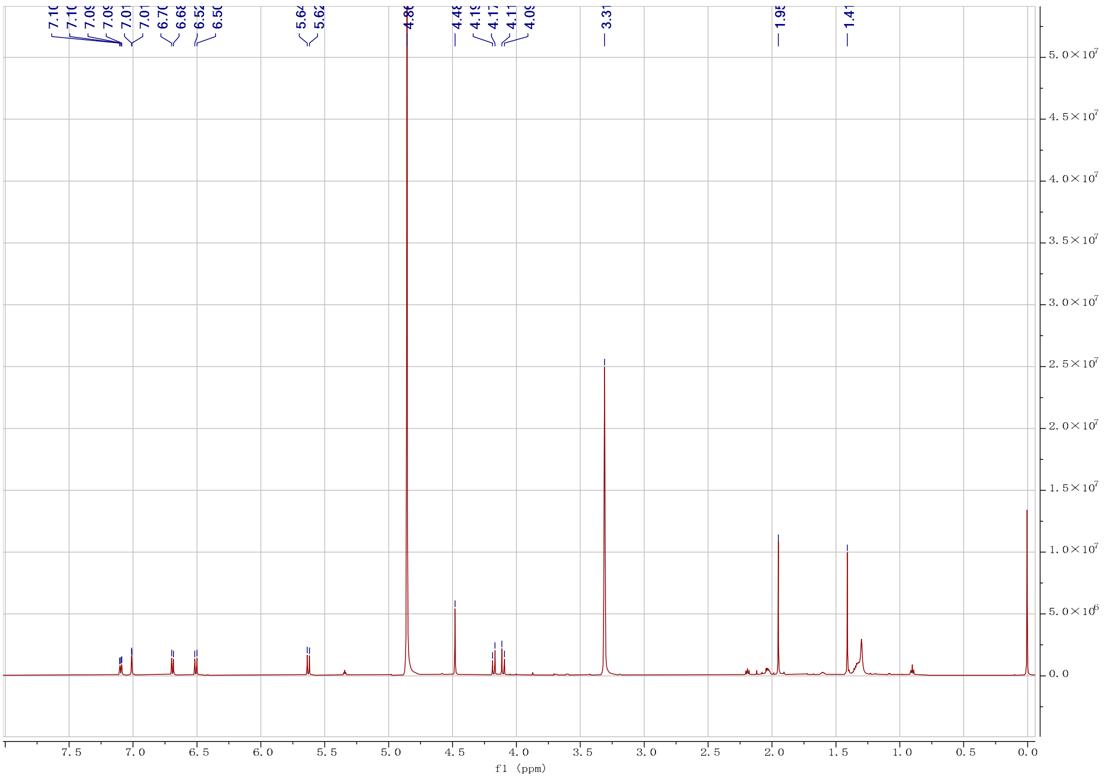

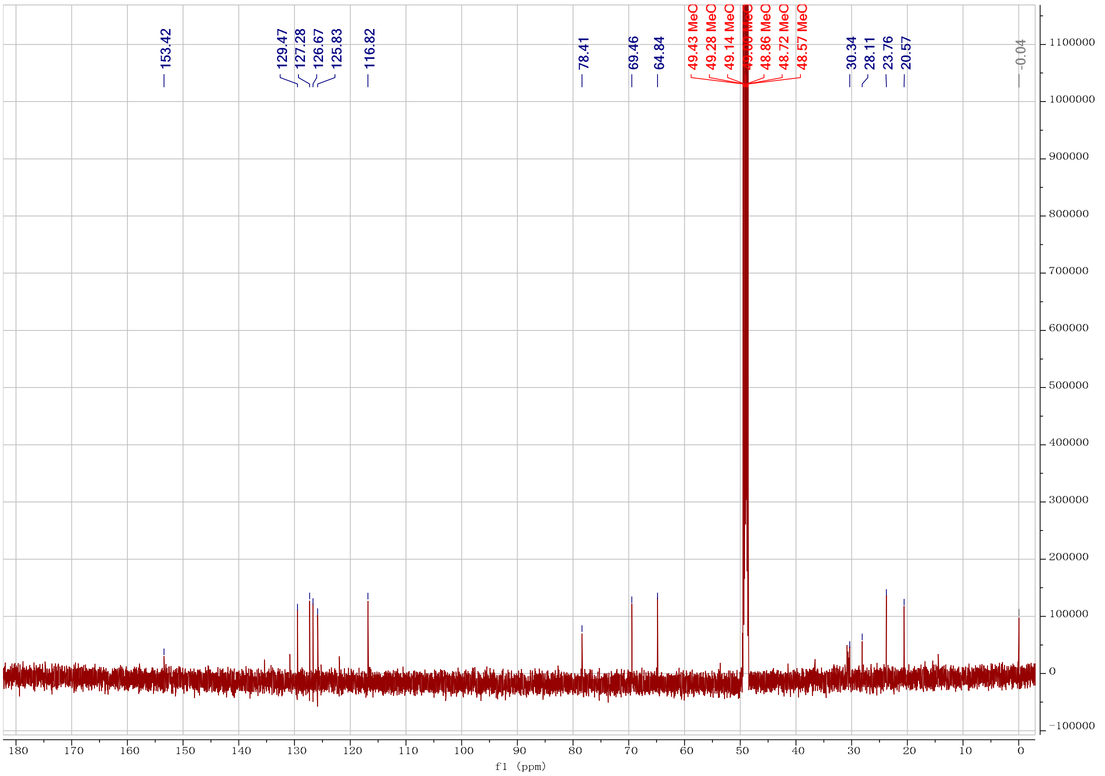

Figure S9. ^{1}H (600 MHz) and ^{13}C (150 MHz) NMR data of **2** in CD_3OD

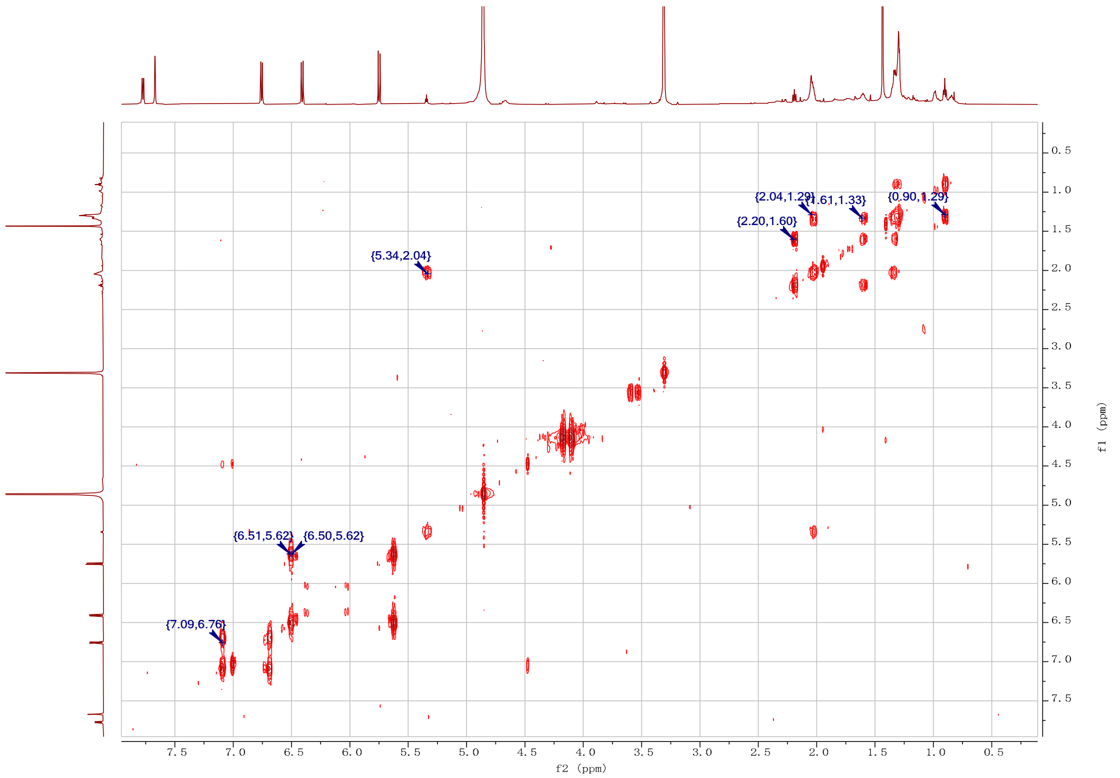

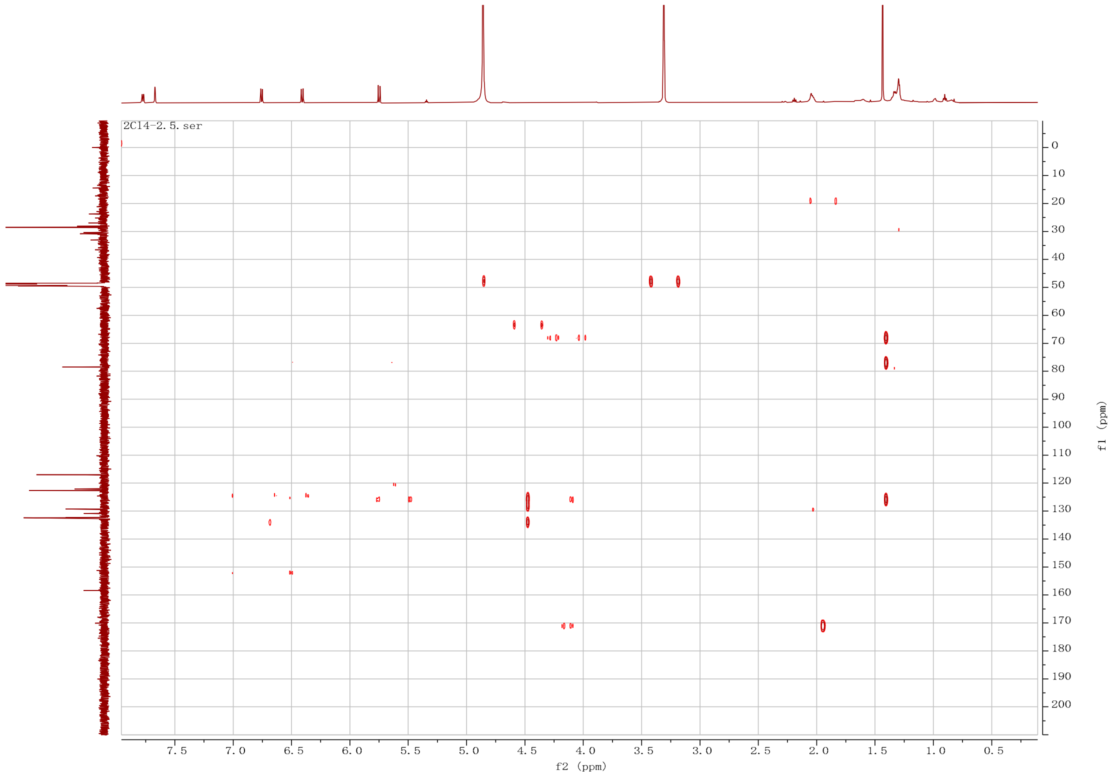

Figure S10. ^{1}H ^{1}H COSY and HMBC spectra (600 MHz, CD_3OD) of **2**

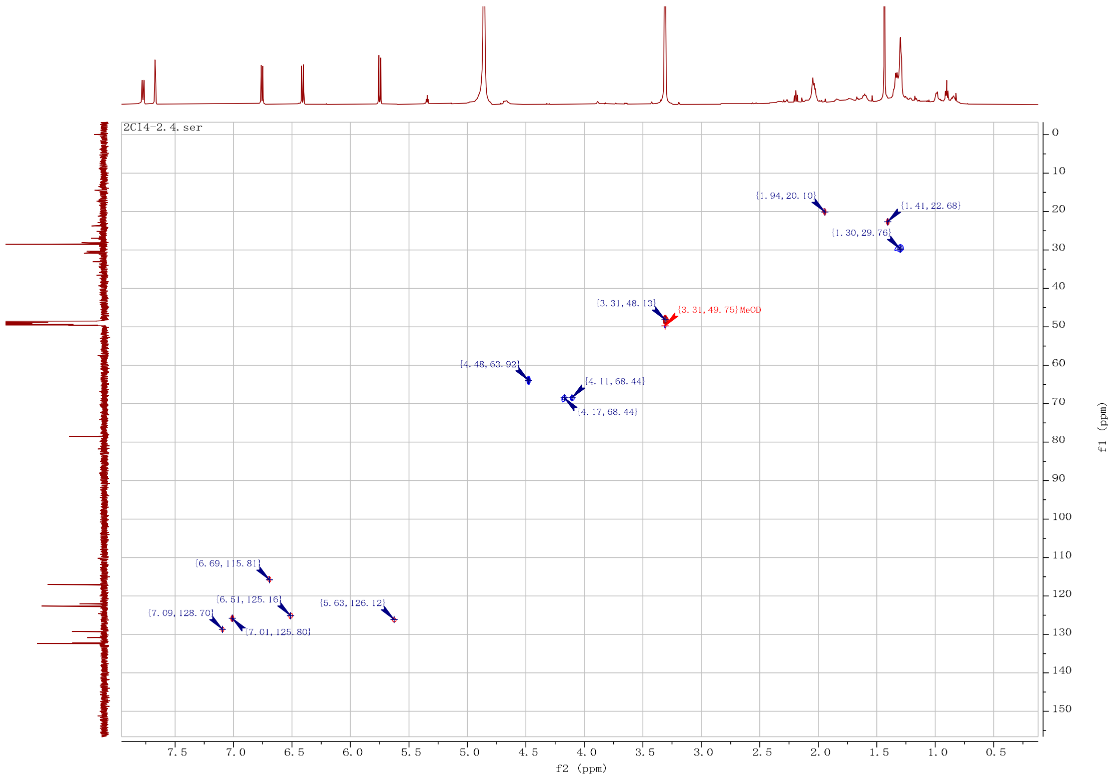

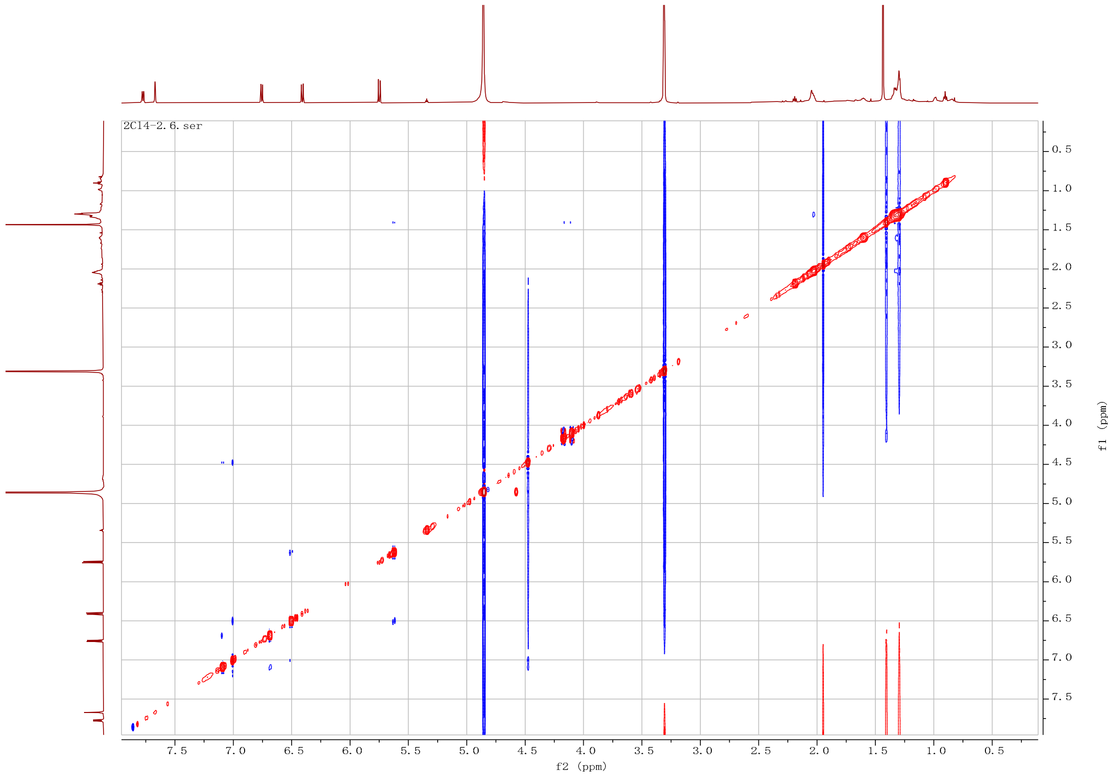

Figure S11. HMQC and NOESY spectra (600 MHz, CD_3OD) of **2**

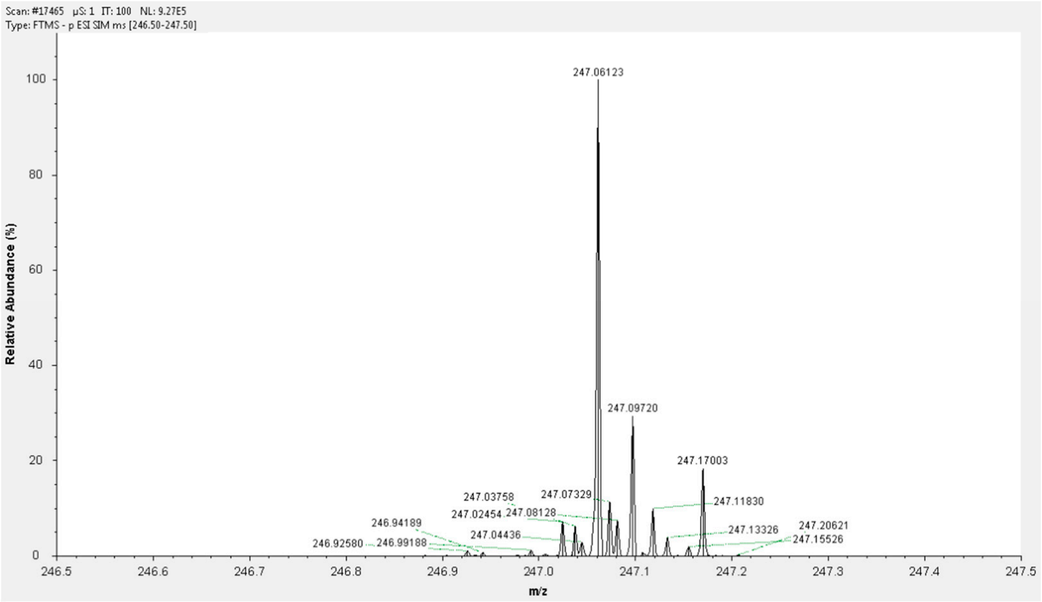

Figure S12. HRESIMS spectrum of **3**

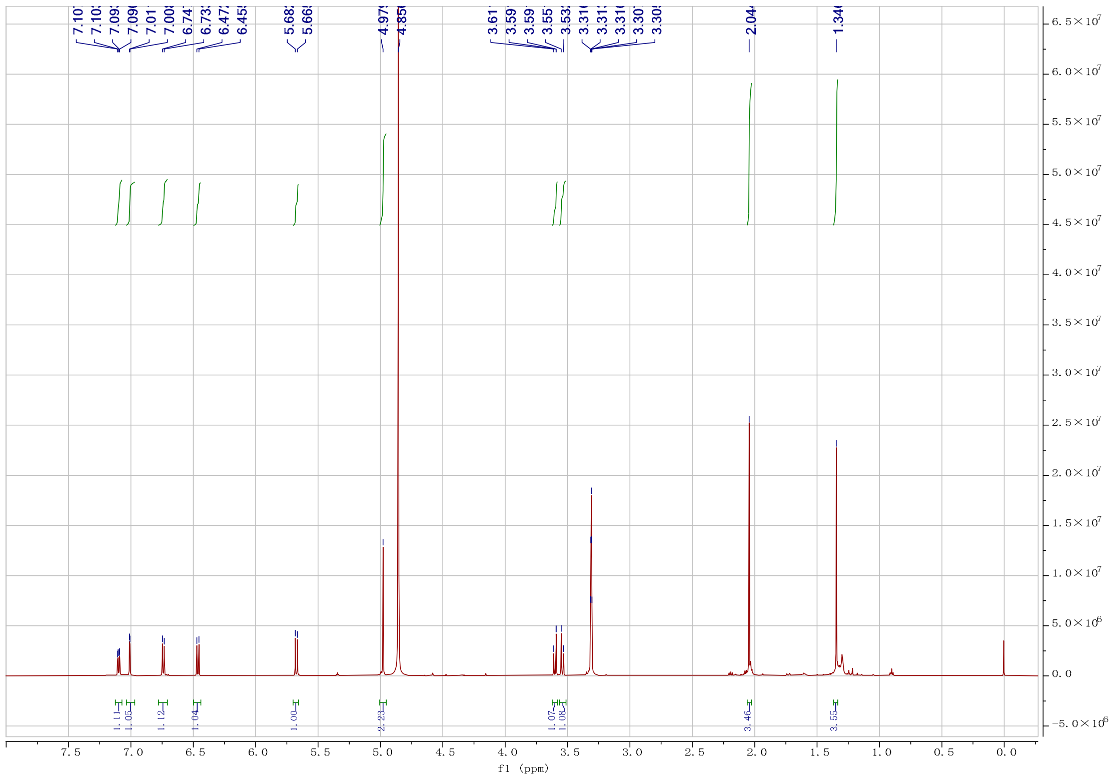

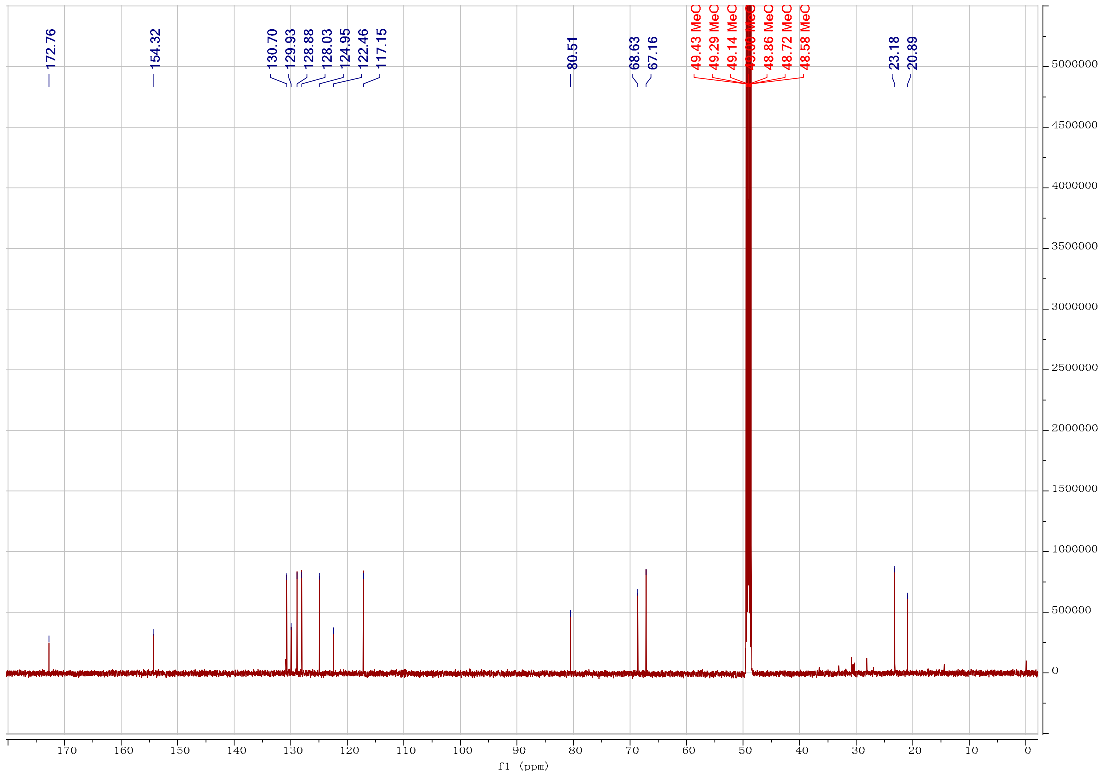

Figure S13. ^{1}H (600 MHz) and ^{13}C (150 MHz) NMR data of **3** in CD_3OD

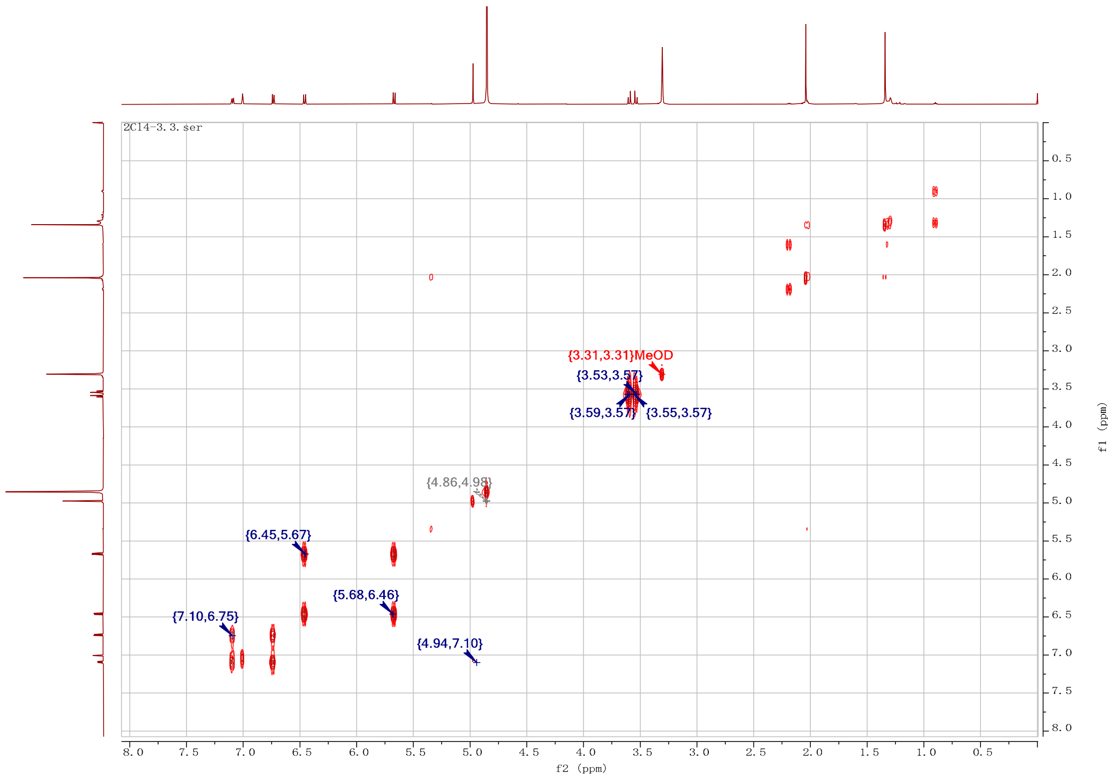

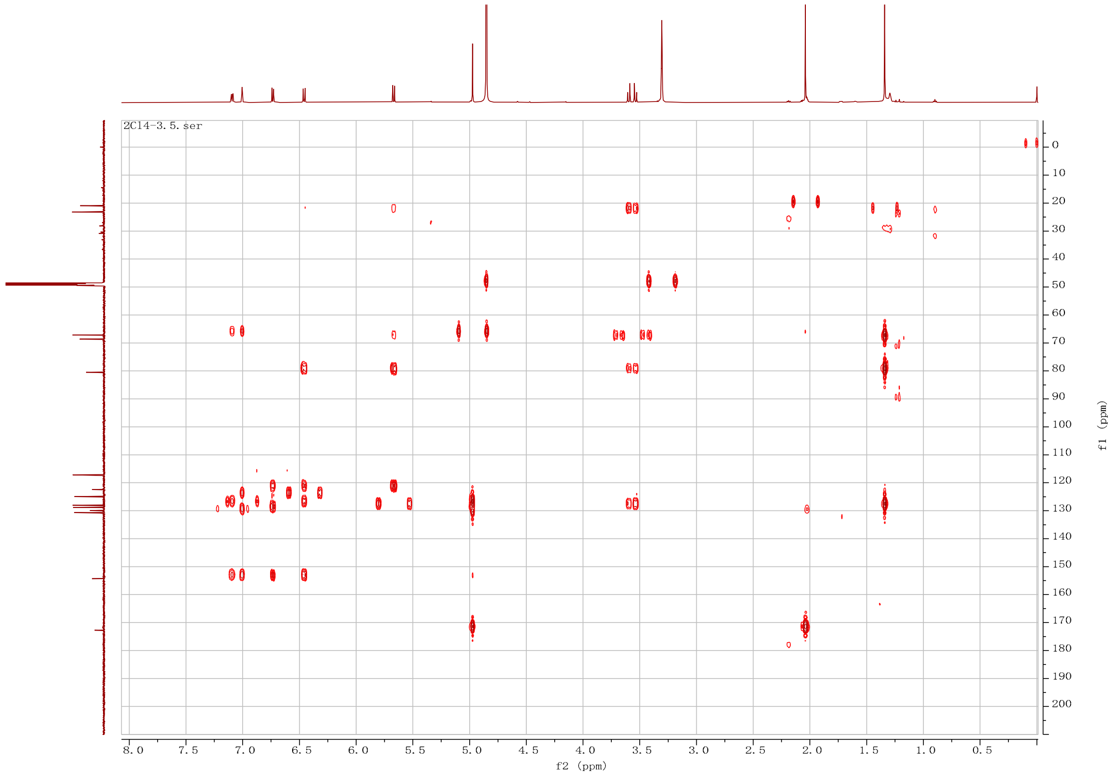

Figure S14. ^1H ^1H COSY and HMBC spectra (600 MHz, CD_3OD) of **3**

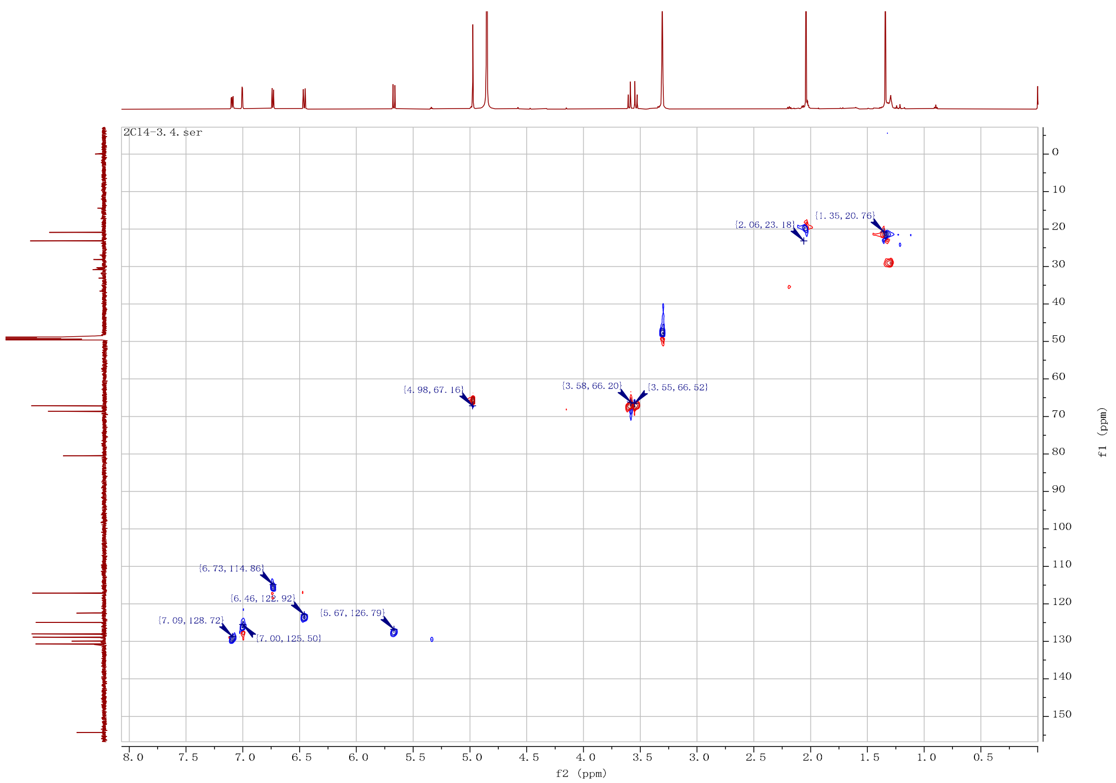

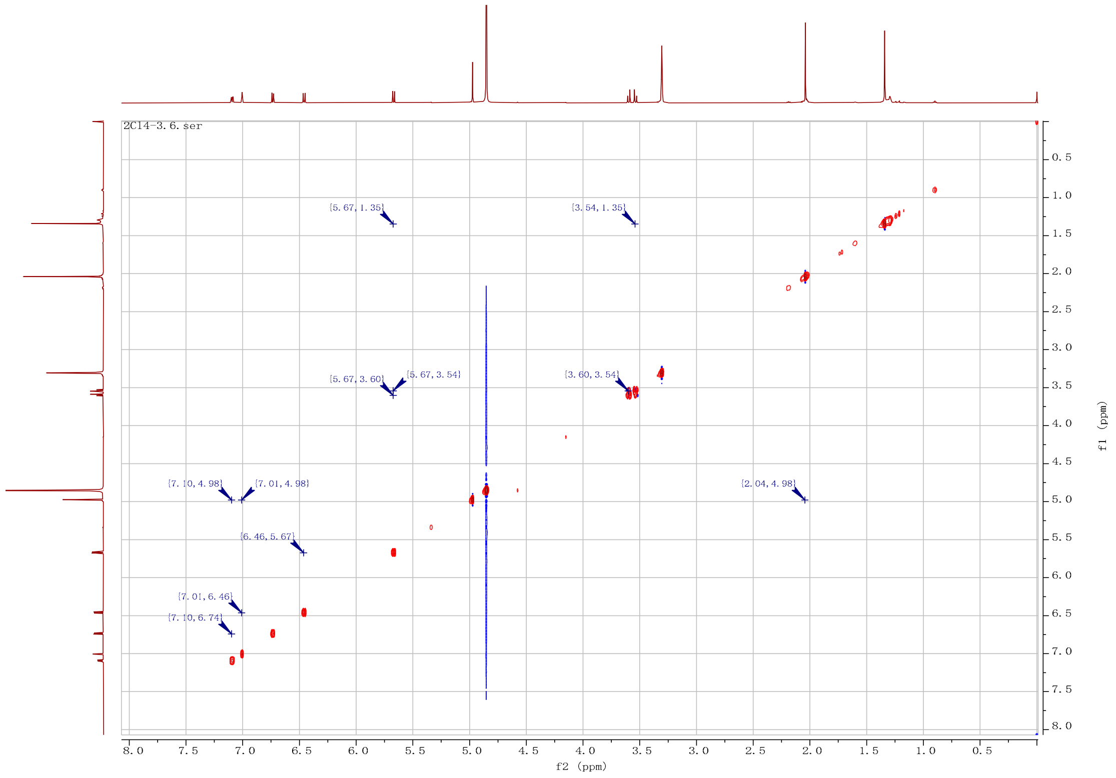

Figure S15. HMQC and NOESY spectra (600 MHz, CD_3OD) of **3**

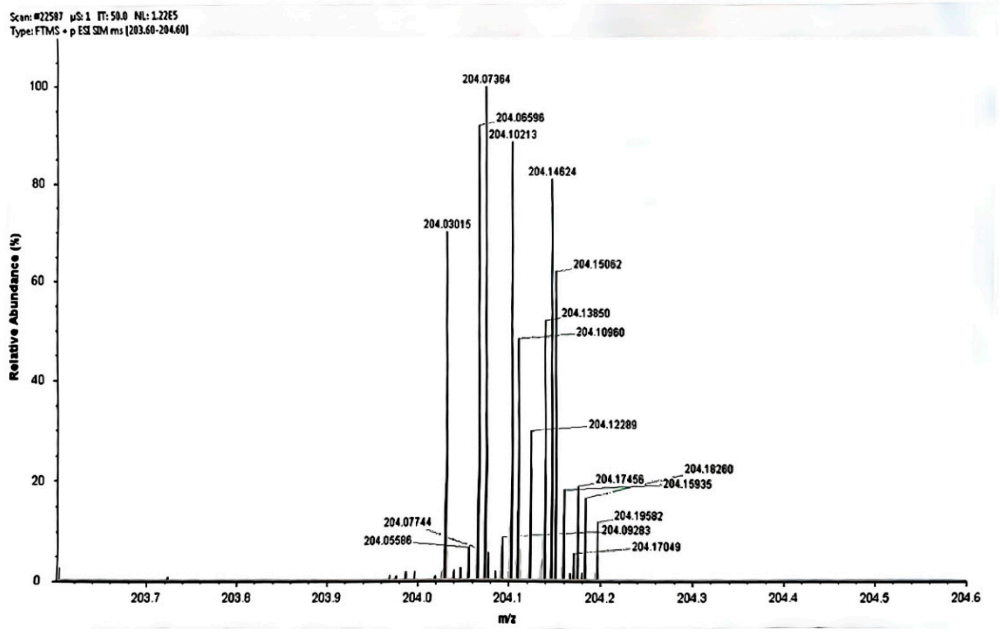

Figure S16. HRESIMS spectrum of **4**

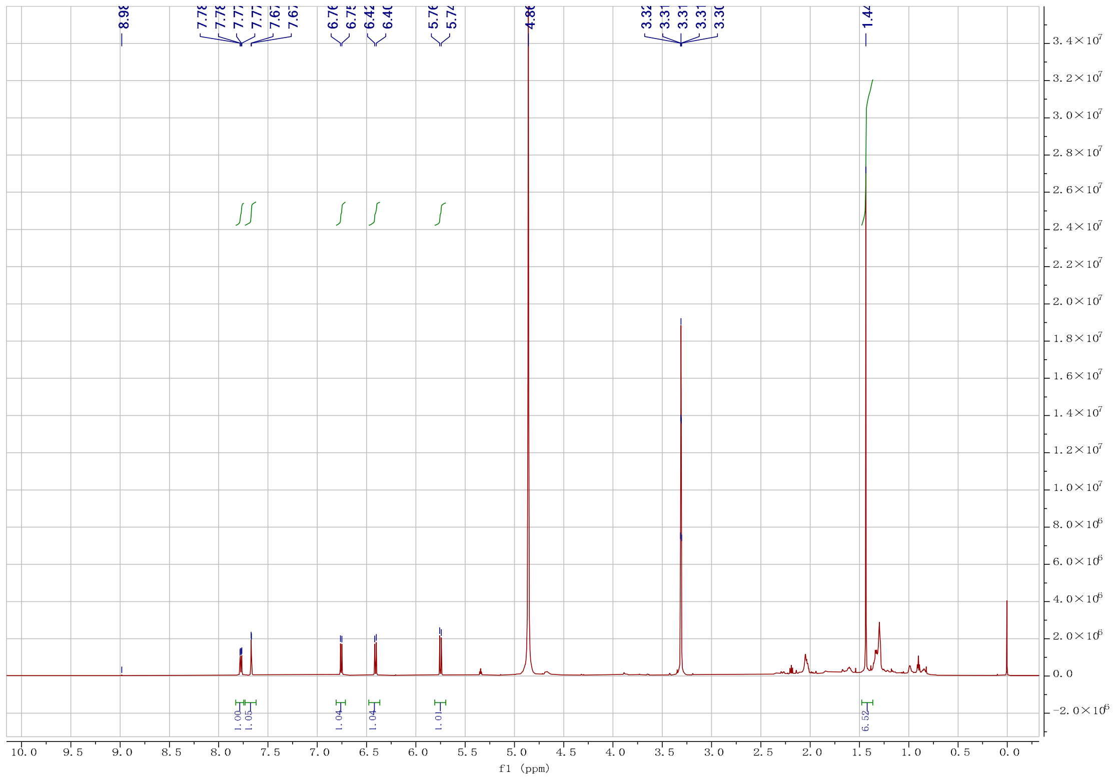

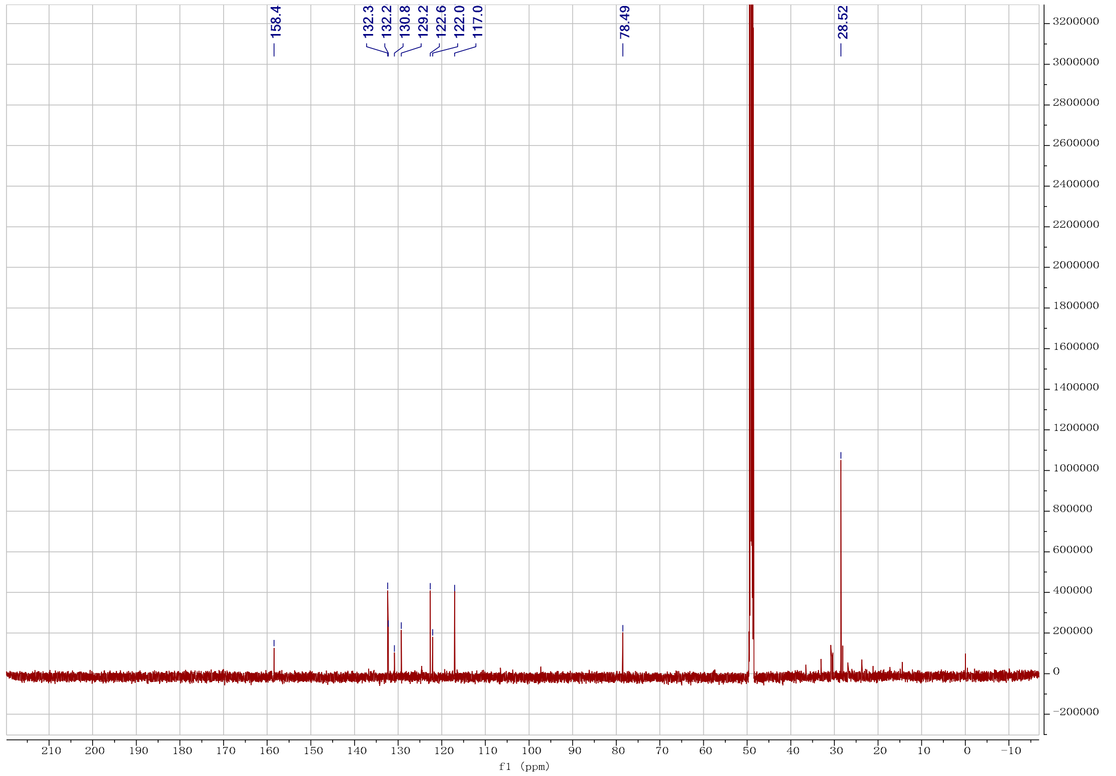

Figure S17. ^{1}H (600 MHz) and ^{13}C (150 MHz) NMR data of **4** in CD_3OD

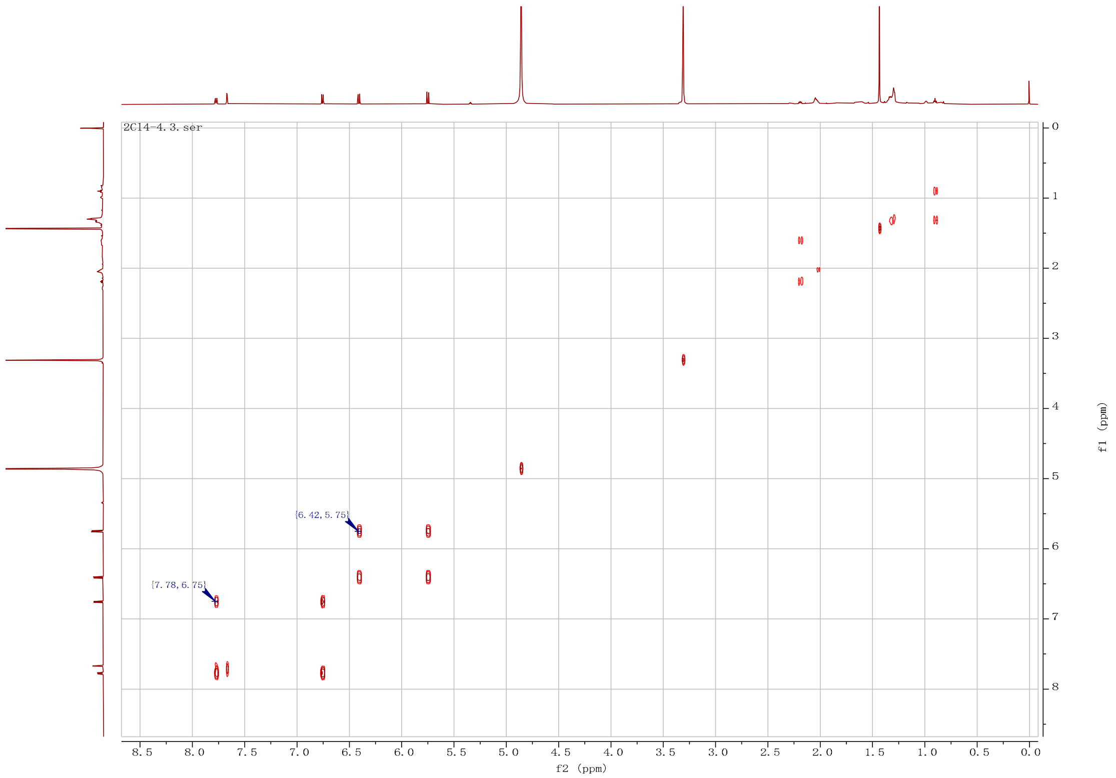

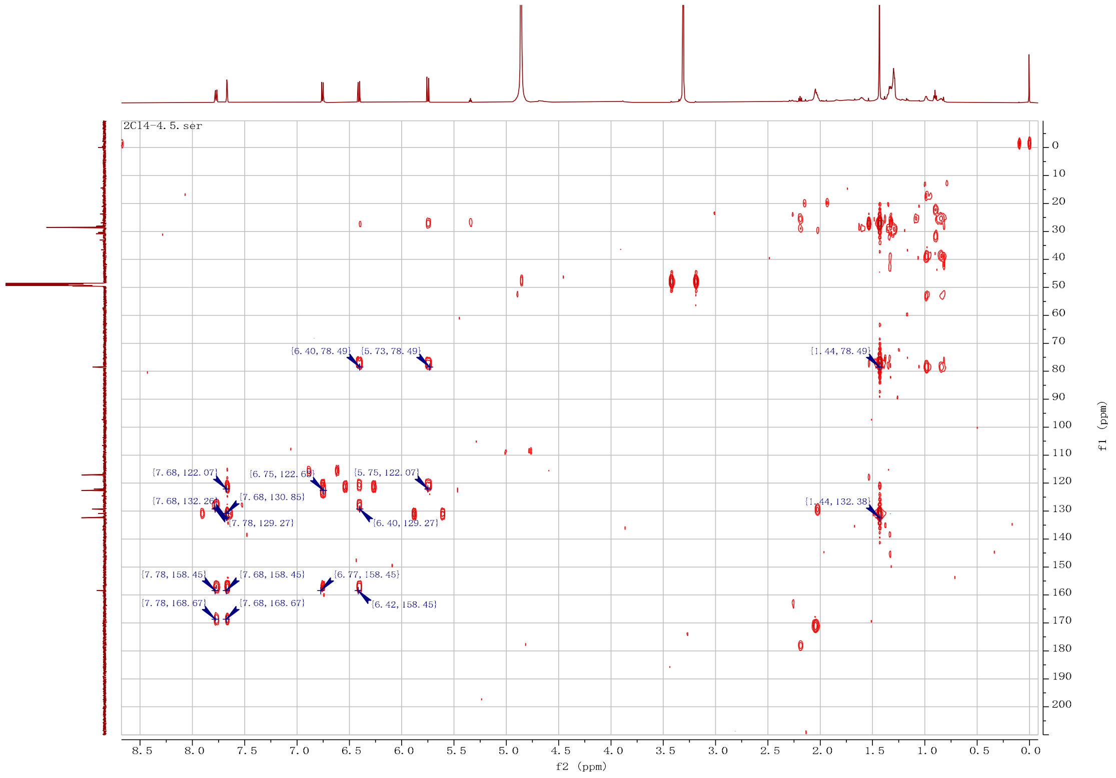

Figure S18. ^{1}H ^{1}H COSY and HMBC spectra (600 MHz, CD_3OD) of **4**

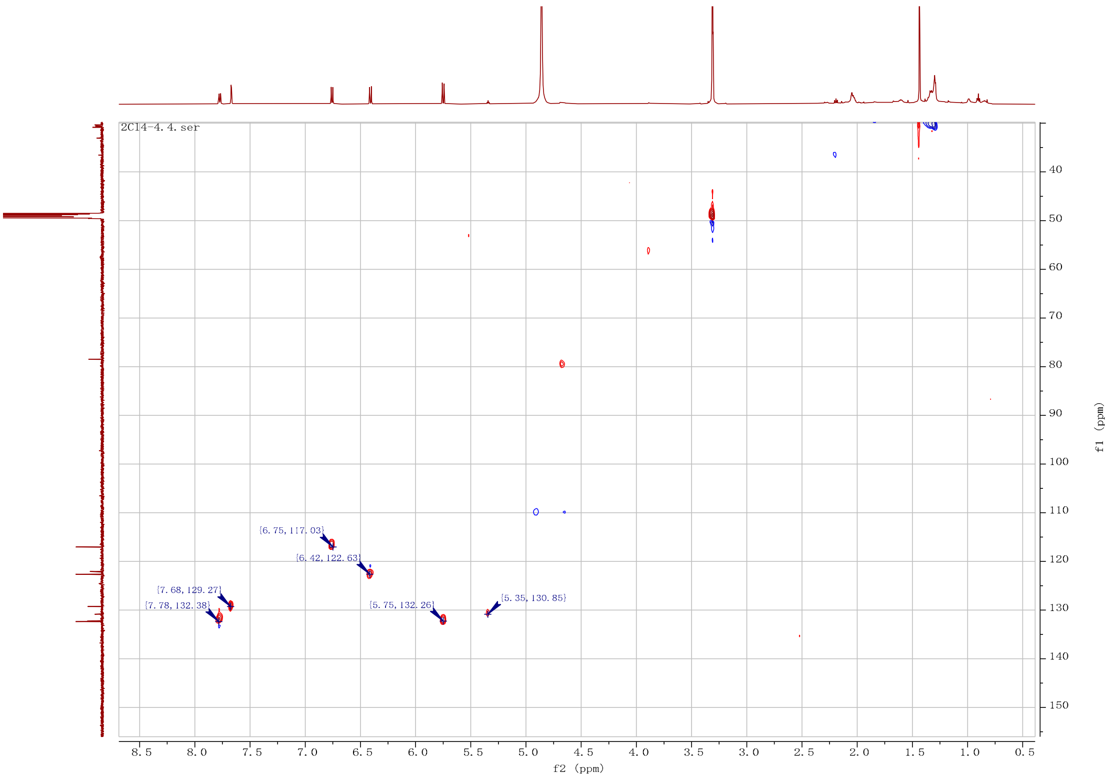

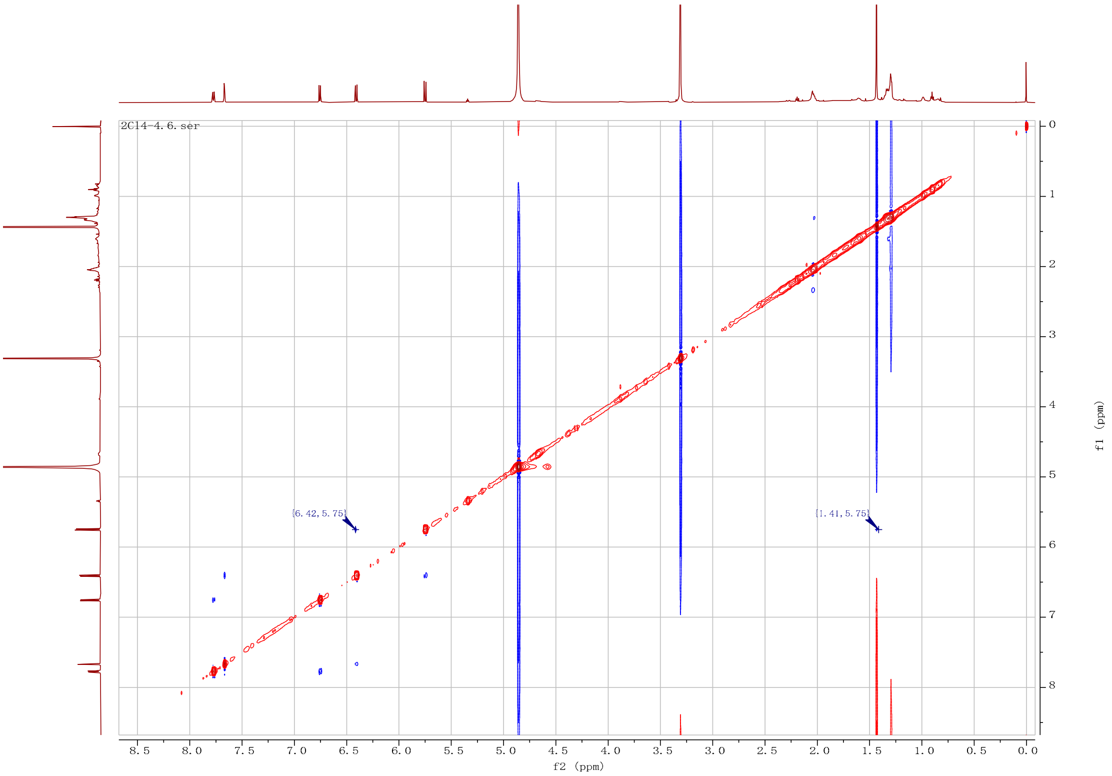

Figure S19. HMQC and NOESY spectra (600 MHz, CD_3OD) of **4**

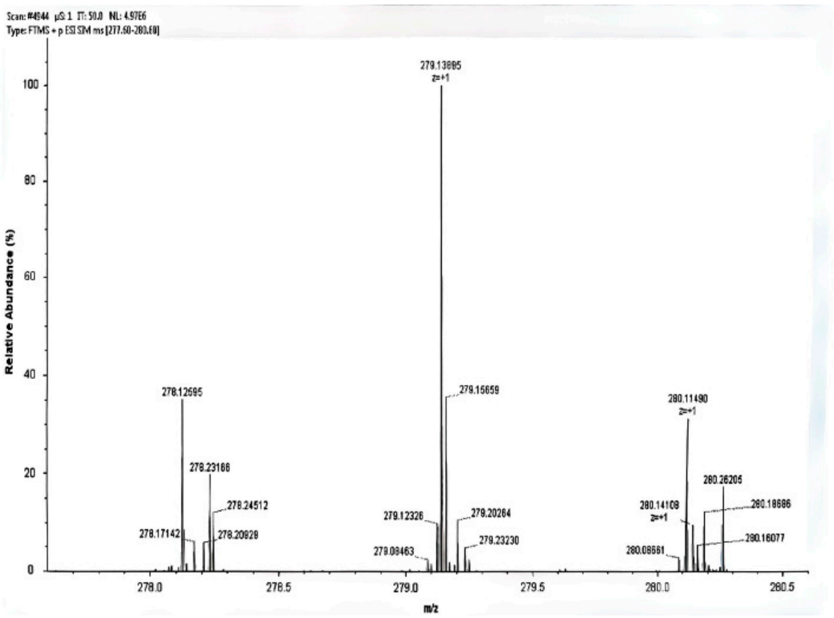

Figure S20. HRESIMS spectrum of **5**

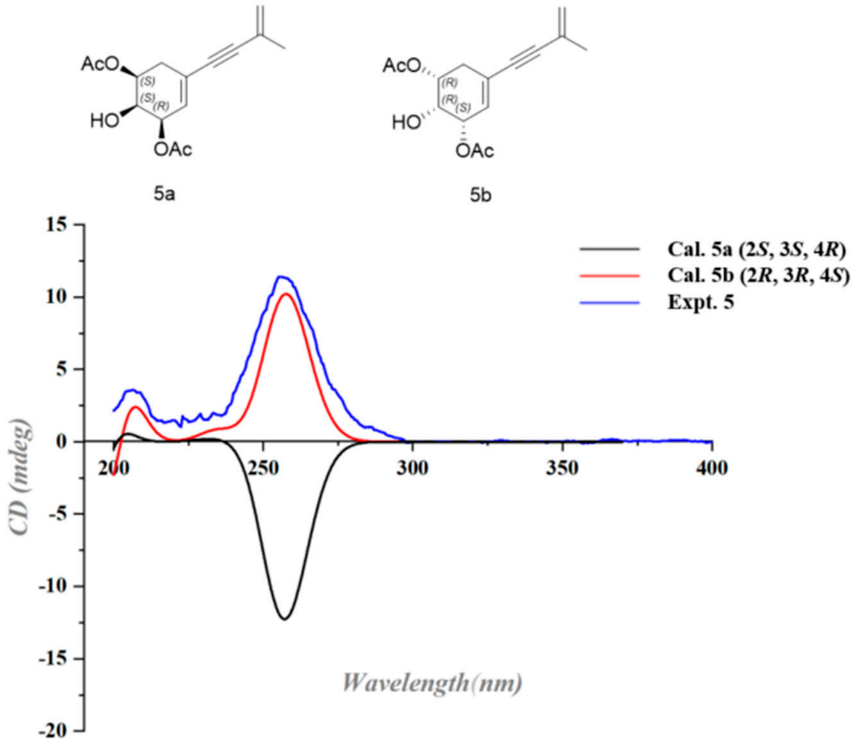

Figure S21. Structures and experimental ECD spectra of **5**

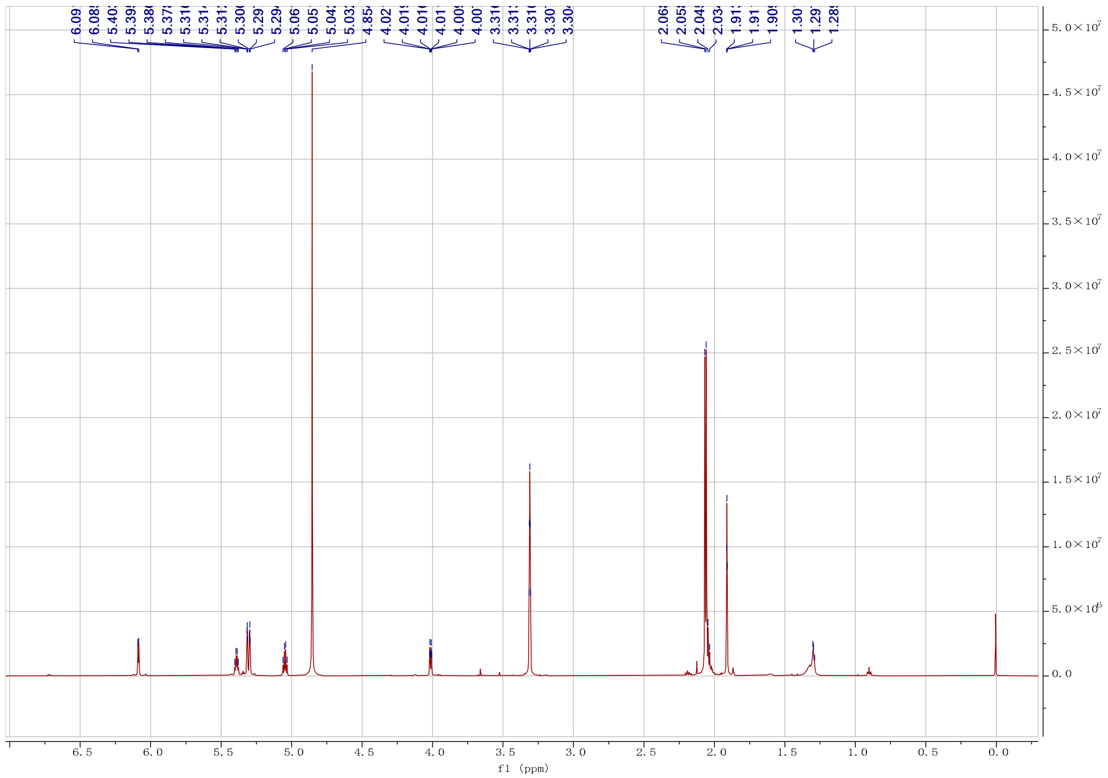

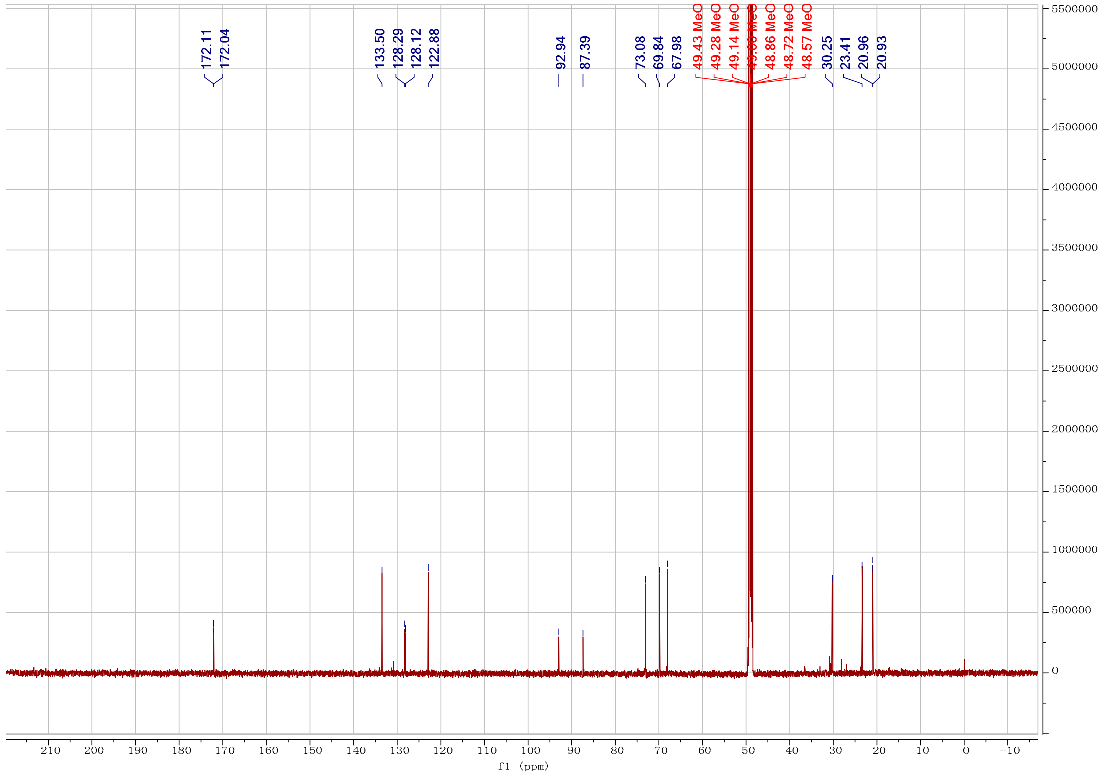

Figure S22. ^{1}H (600 MHz) and ^{13}C (150 MHz) NMR data of **5** in CD_3OD

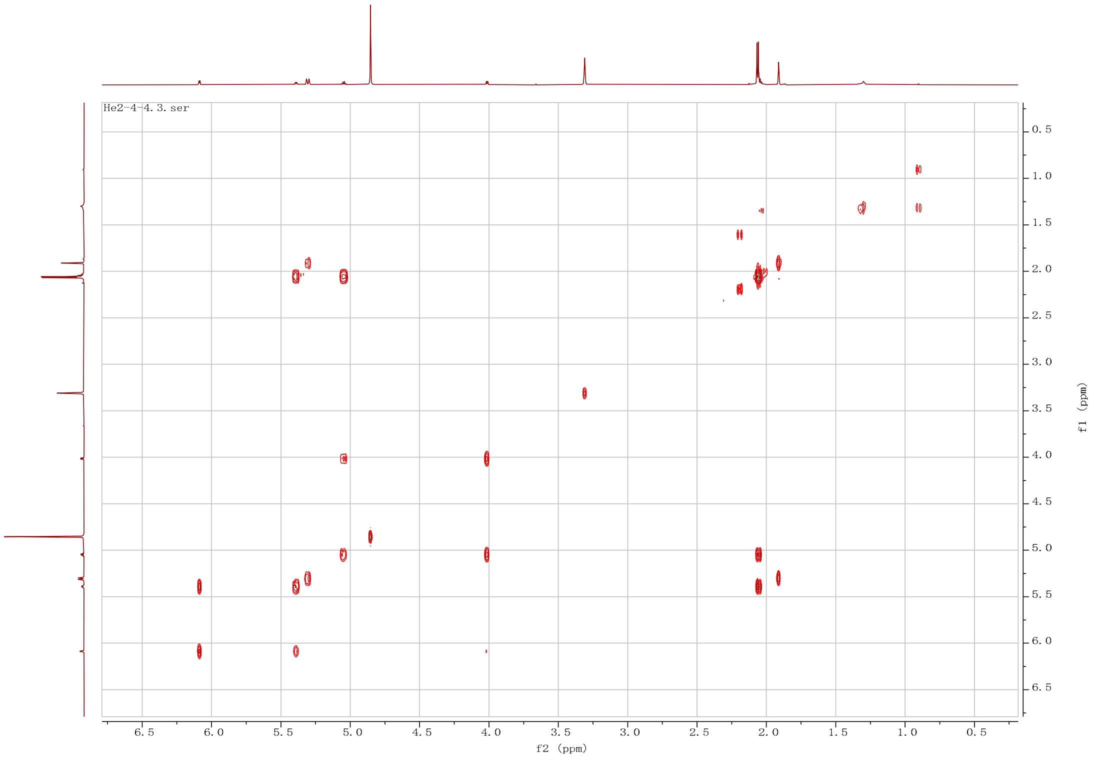

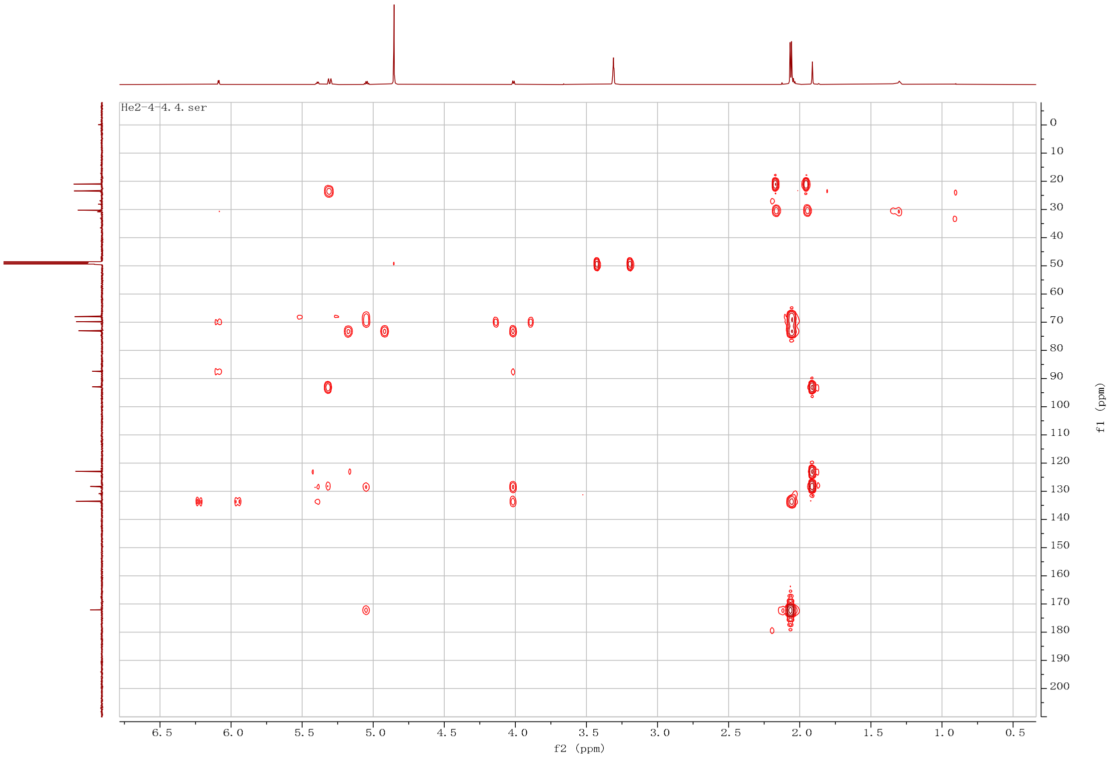

Figure S23. ^{1}H ^{1}H COSY and HMBC spectra (600 MHz, CD_3OD) of **5**

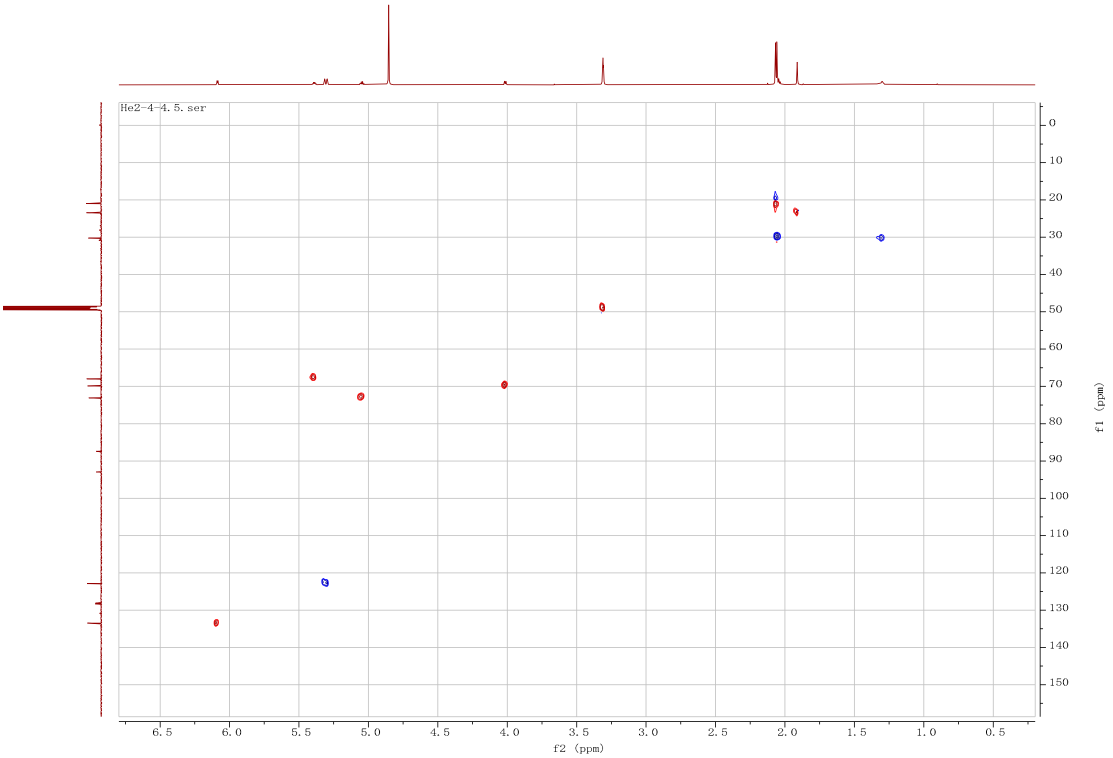

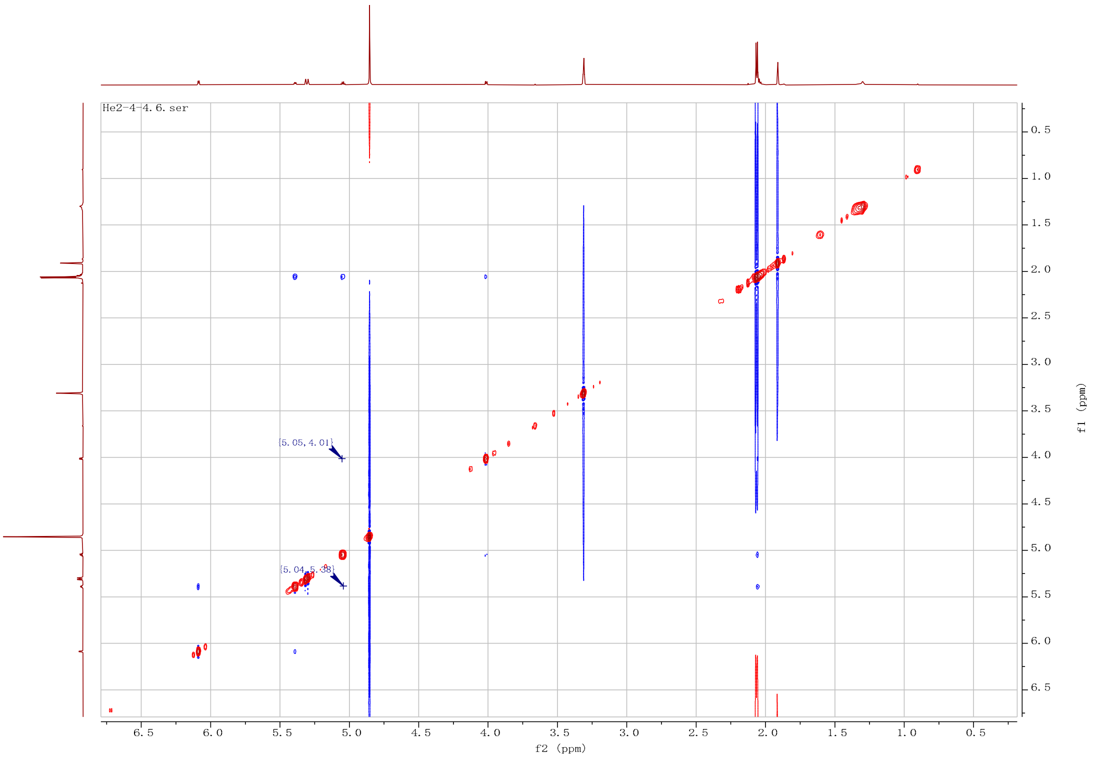

Figure S24. HMQC and NOESY spectra (600 MHz, CD_3OD) of **5**

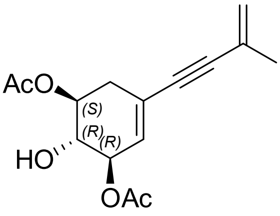

5a

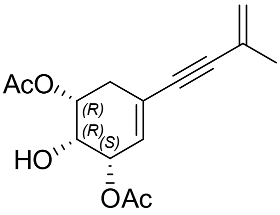

5b

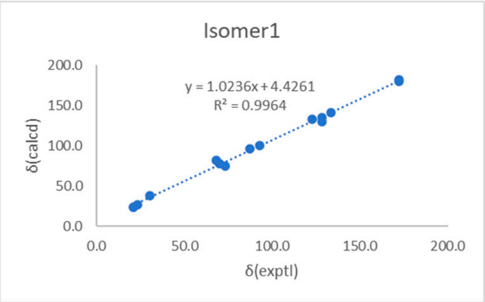

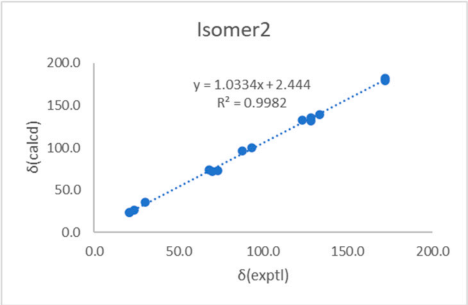

Settings		Type of data (shifts)				TMS 1H	31.795	TMS 13C	188.488
Default		Shielding tensors				Default	μ	σ	ν
						13Cu,sp2	6.885	2.144	6.663
						13Cu,sp3	4.154	1.787	8.592
Functional		Solvent?		Basis Set		1Hu,sp2	0.297	0.117	5.315
mPW1PW91		PCM		6-311G(d,p)		1Hu,sp3	-0.039	0.112	2.599
						13Cs	-	1.491	5.226
						1Hs	-	0.093	2.784
Isomer Nº		1	2	3	4	5	6	7	8
DP4+ (%)	H data	45.08%	54.92%	-	-	-	-	-	-
	C data	0.00%	100.00%	-	-	-	-	-	-
	All data	0.00%	100.00%	-	-	-	-	-	-

Figure S25. Regression analyses of experimental versus calculated 13C NMR chemical shifts of model 5a and 5b

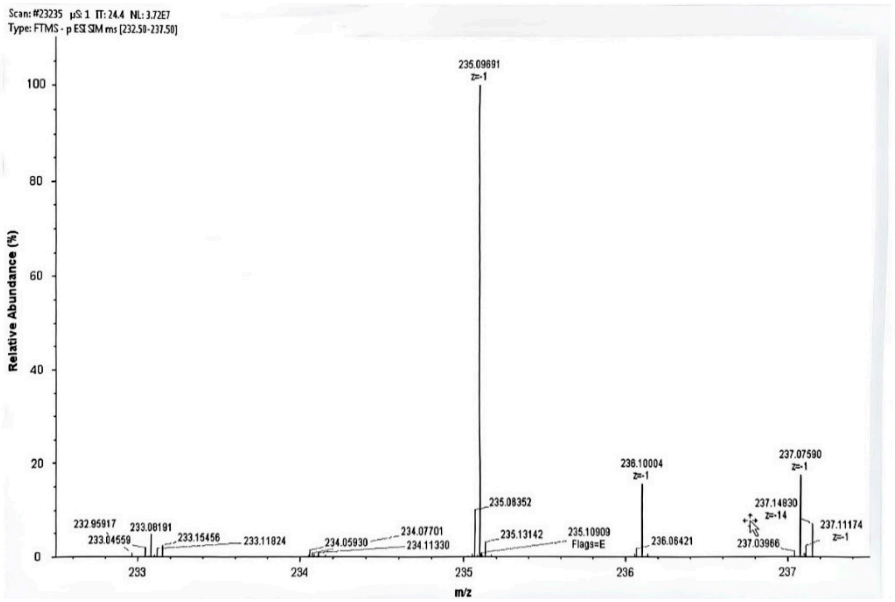

Figure S26. HRESIMS spectrum of **6**

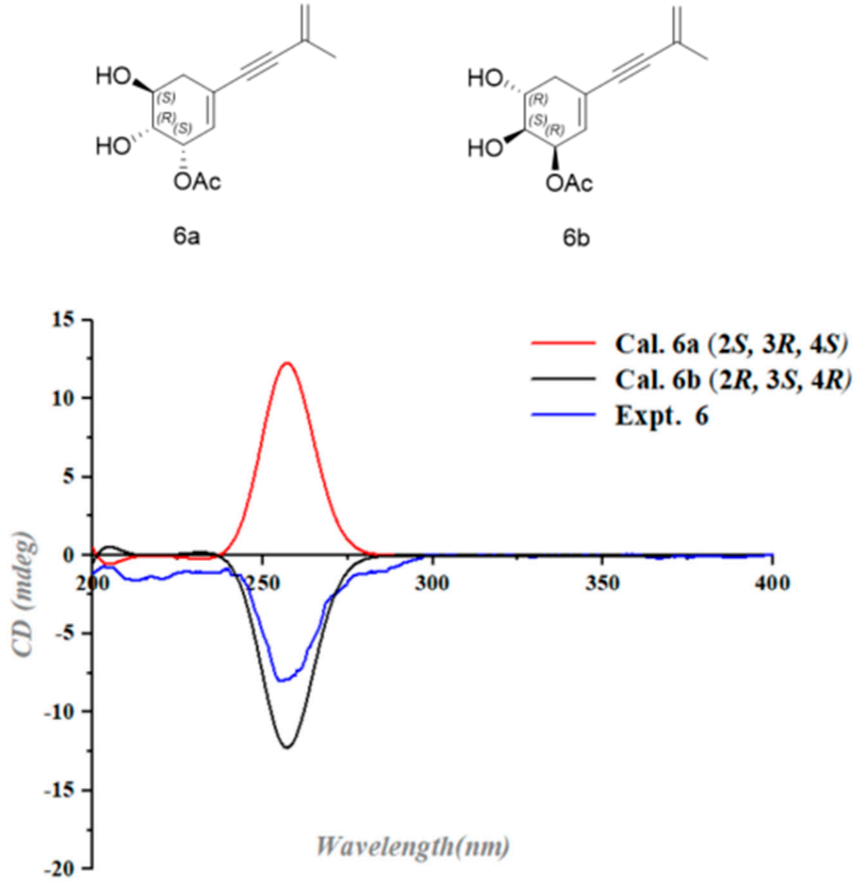

Figure S27. Structures and experimental ECD spectra of **6**

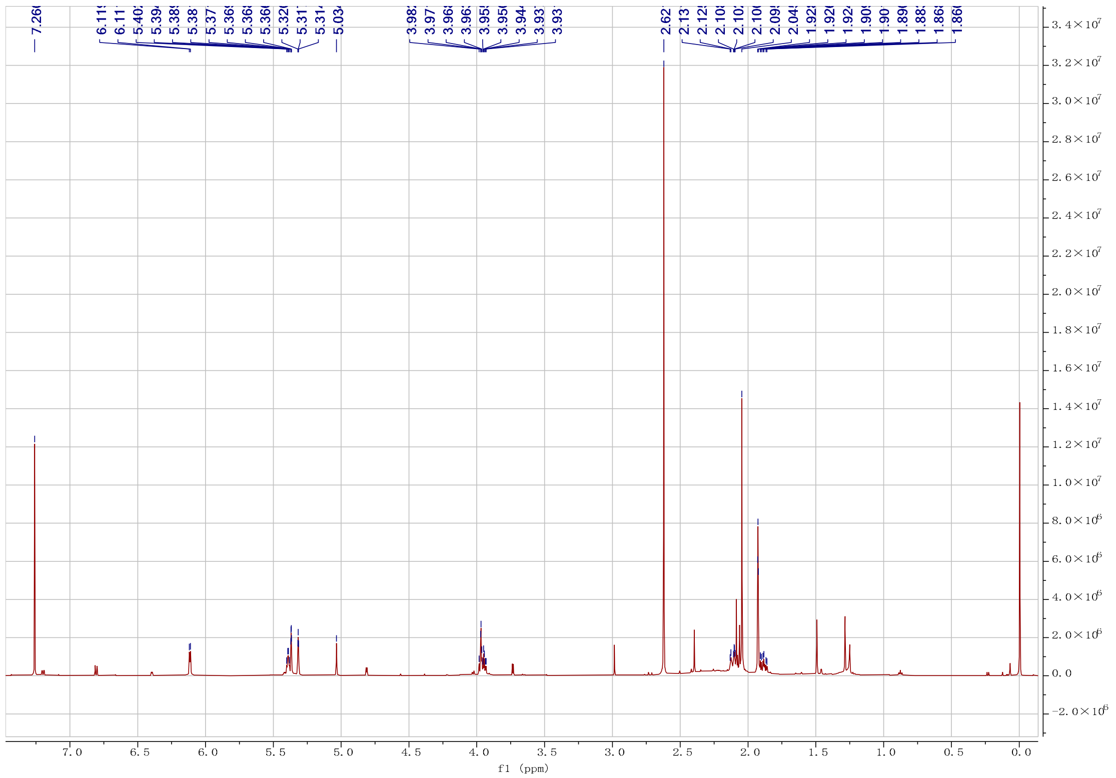

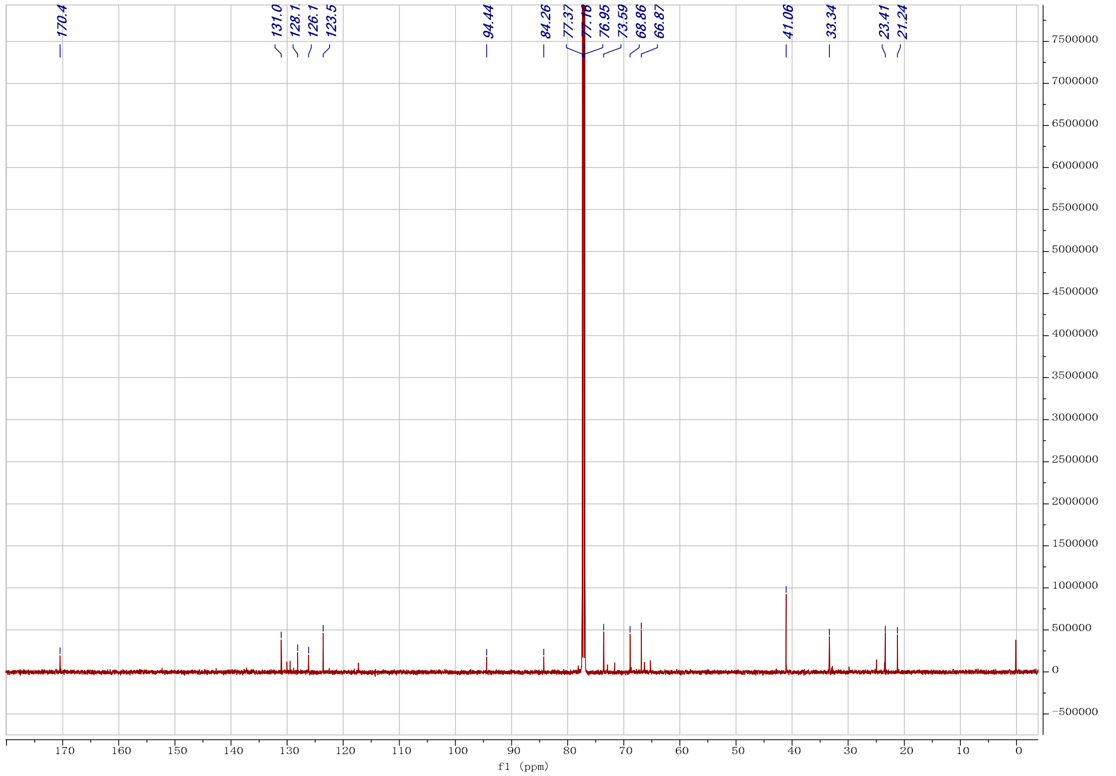

Figure S28. ^{1}H (600 MHz) and ^{13}C (150 MHz) NMR data of **6** in $CDCl_3$

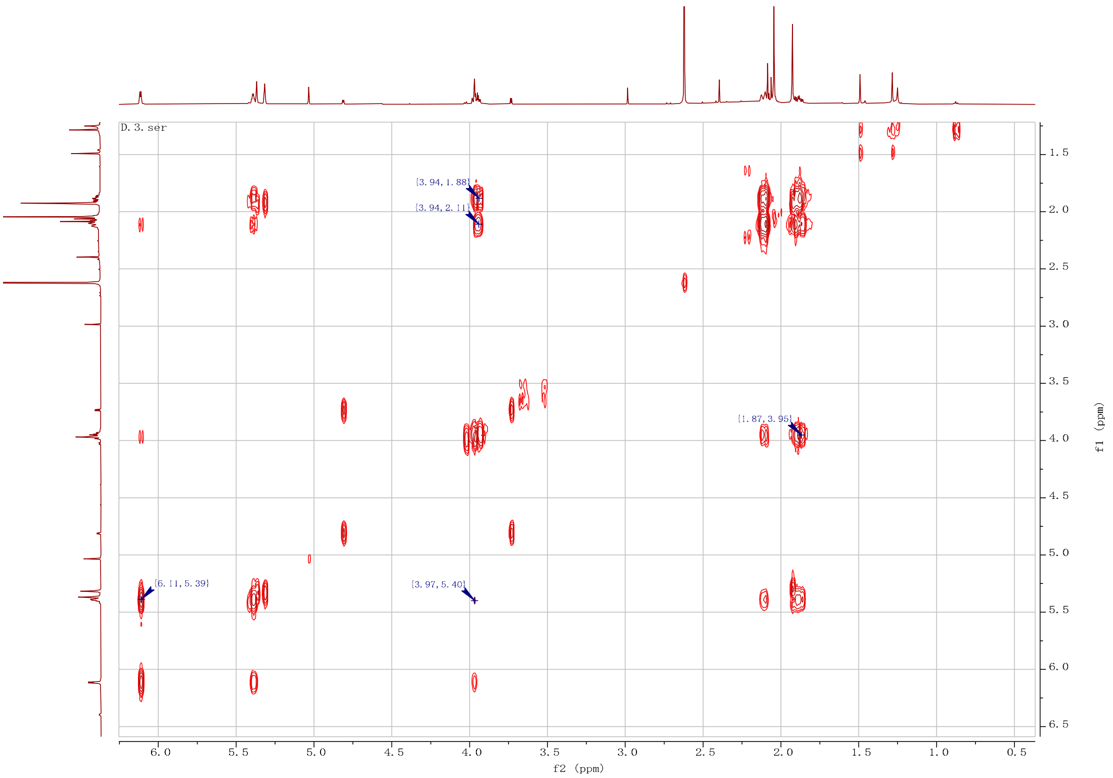

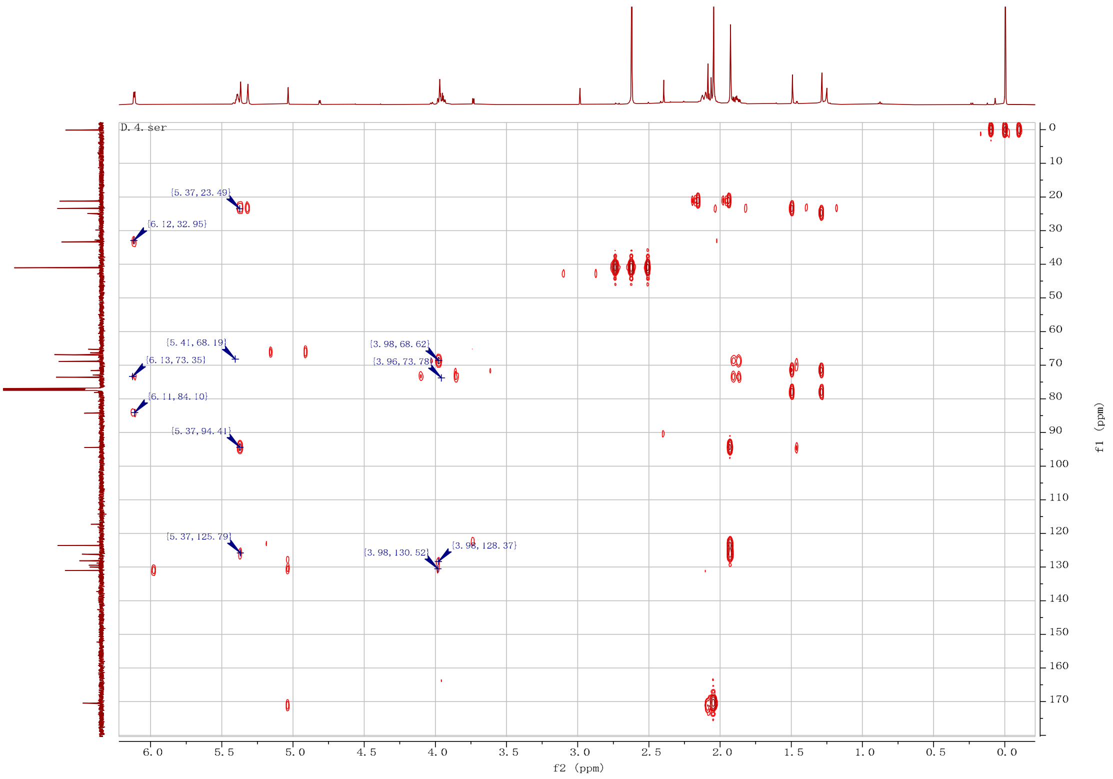

Figure S29. ^{1}H ^{1}H COSY and HMBC spectra (600 MHz, $CDCl_3$) of **6**

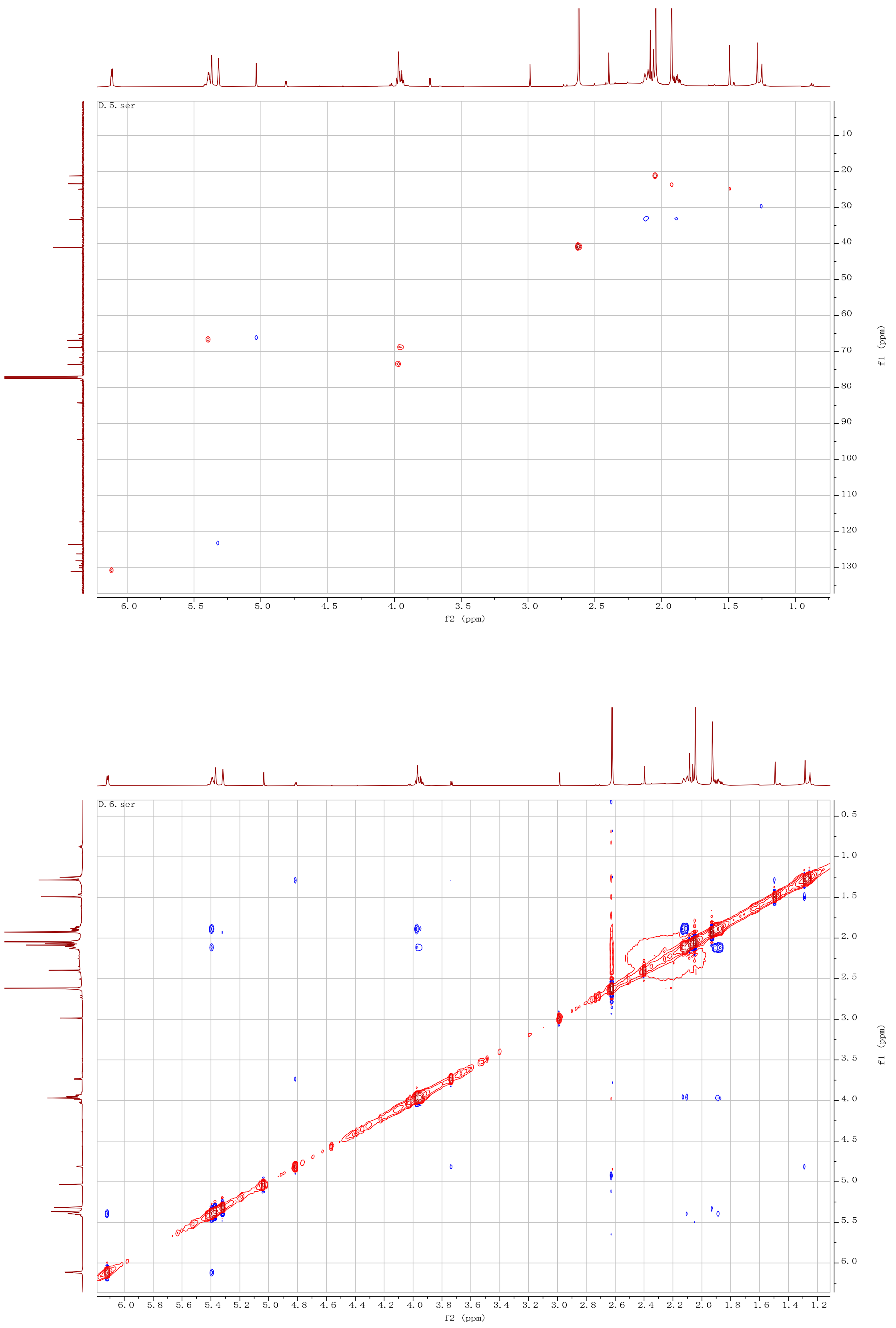

Figure S30. HMQC and NOESY spectra (600 MHz, $CDCl_3$) of **6**

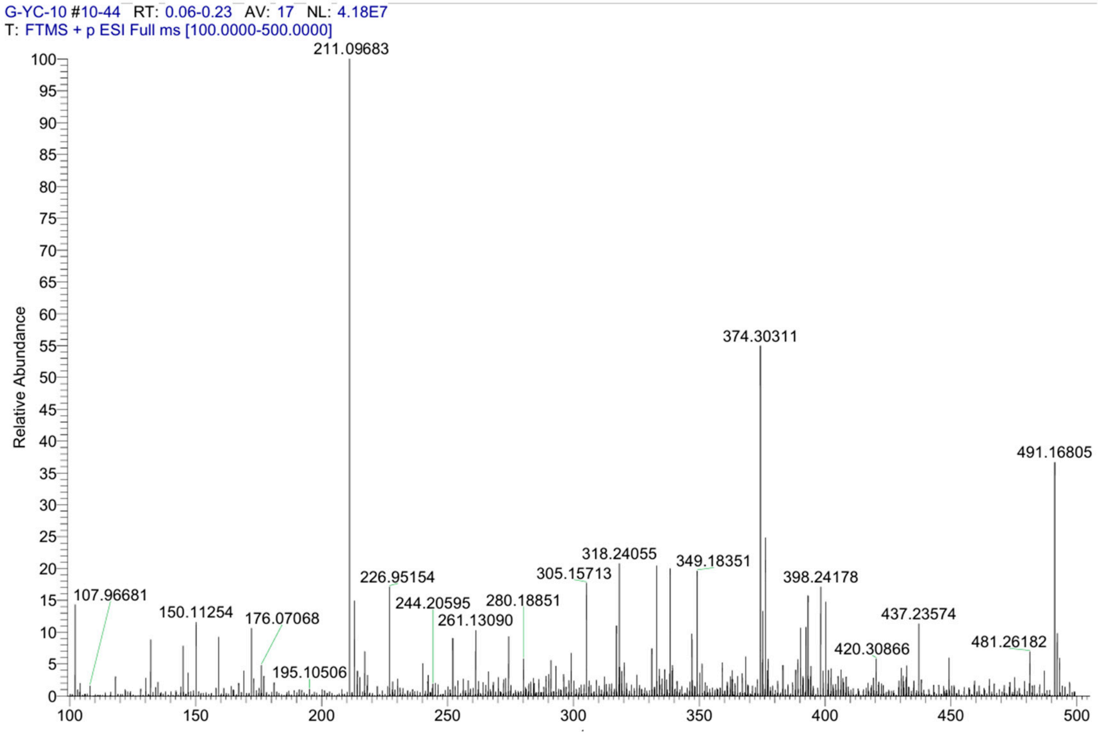

Figure S31. HRESIMS spectrum of **7**

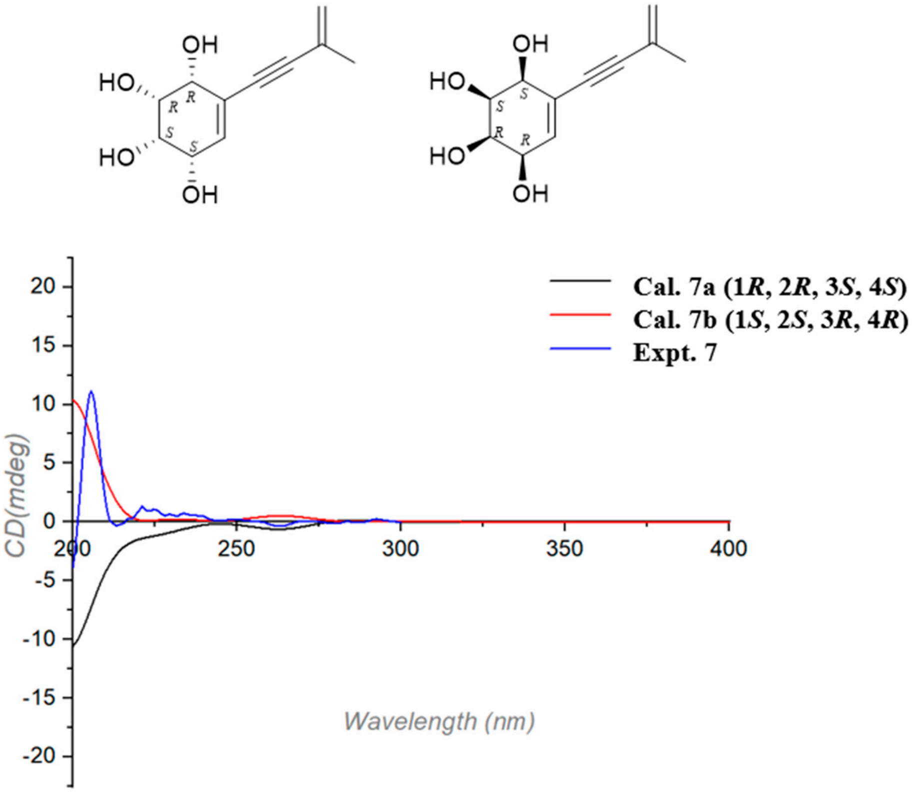

Figure S32. Structures and experimental ECD spectra of **7**

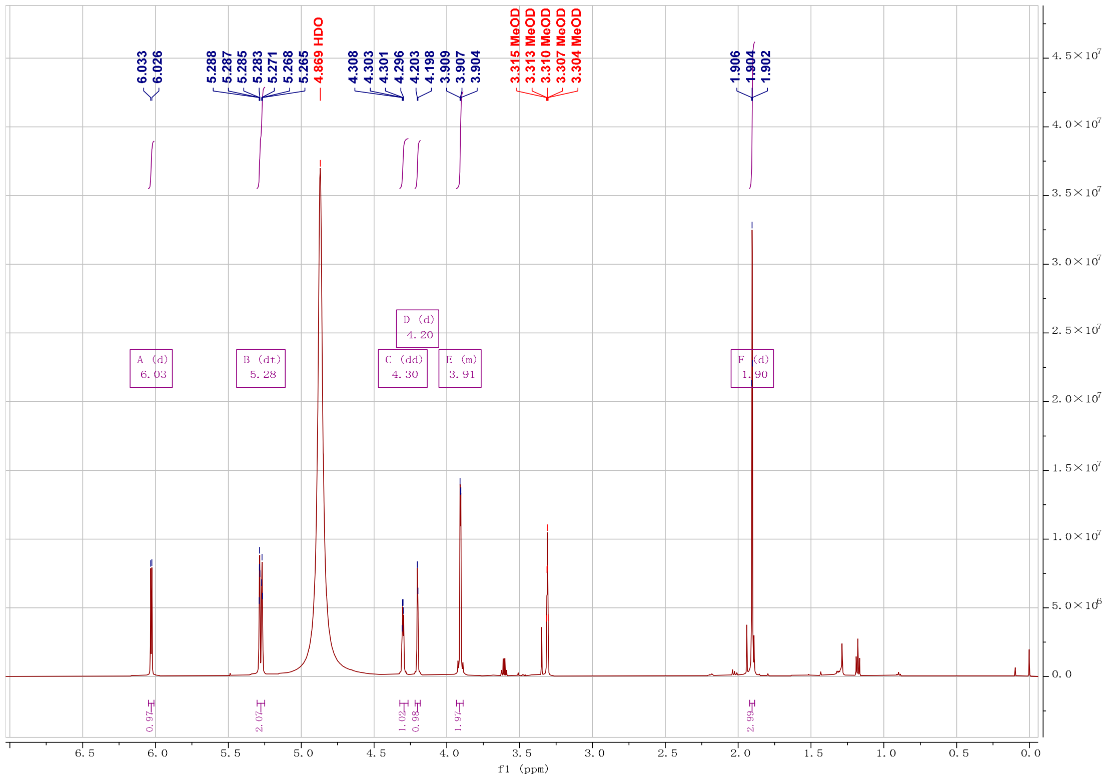

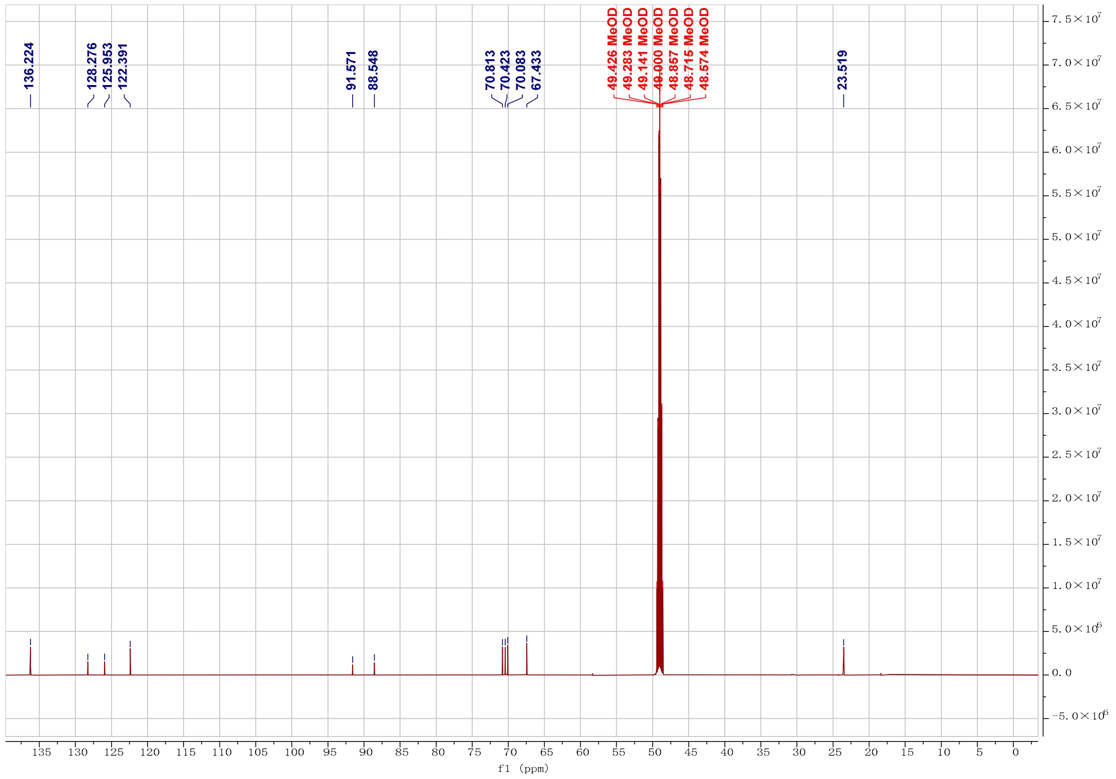

Figure S33. ^{1}H (600 MHz) and ^{13}C (150 MHz) NMR data of **7** in CD_3OD

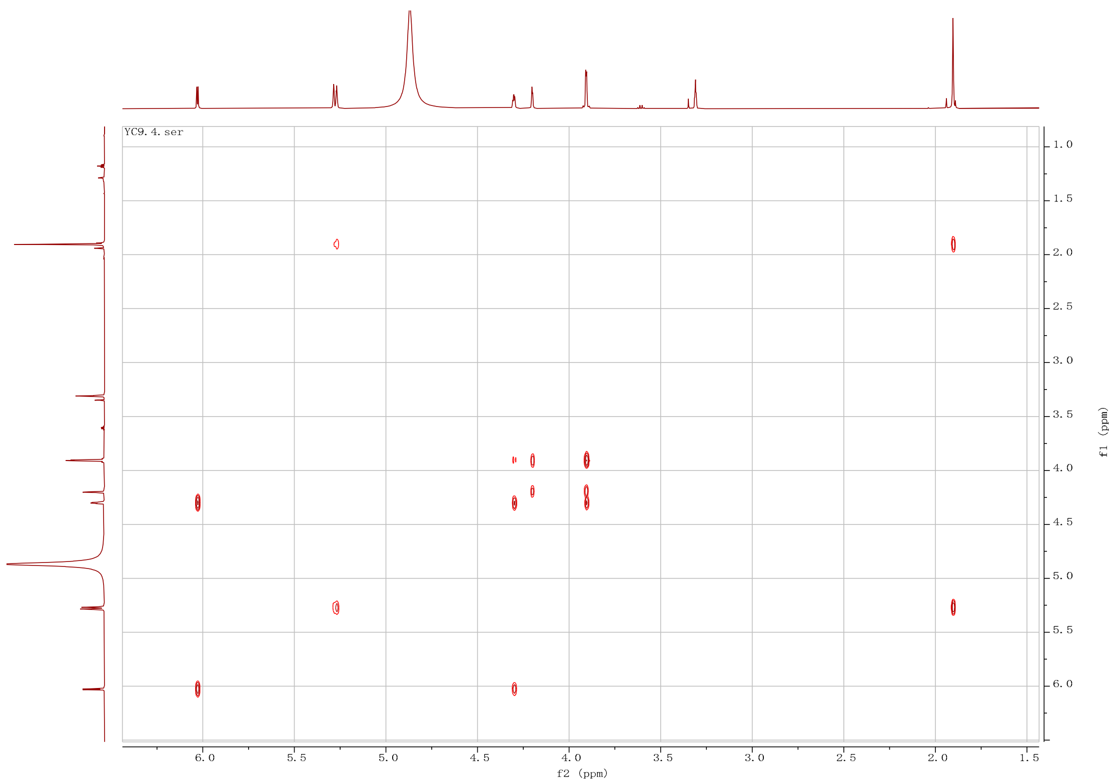

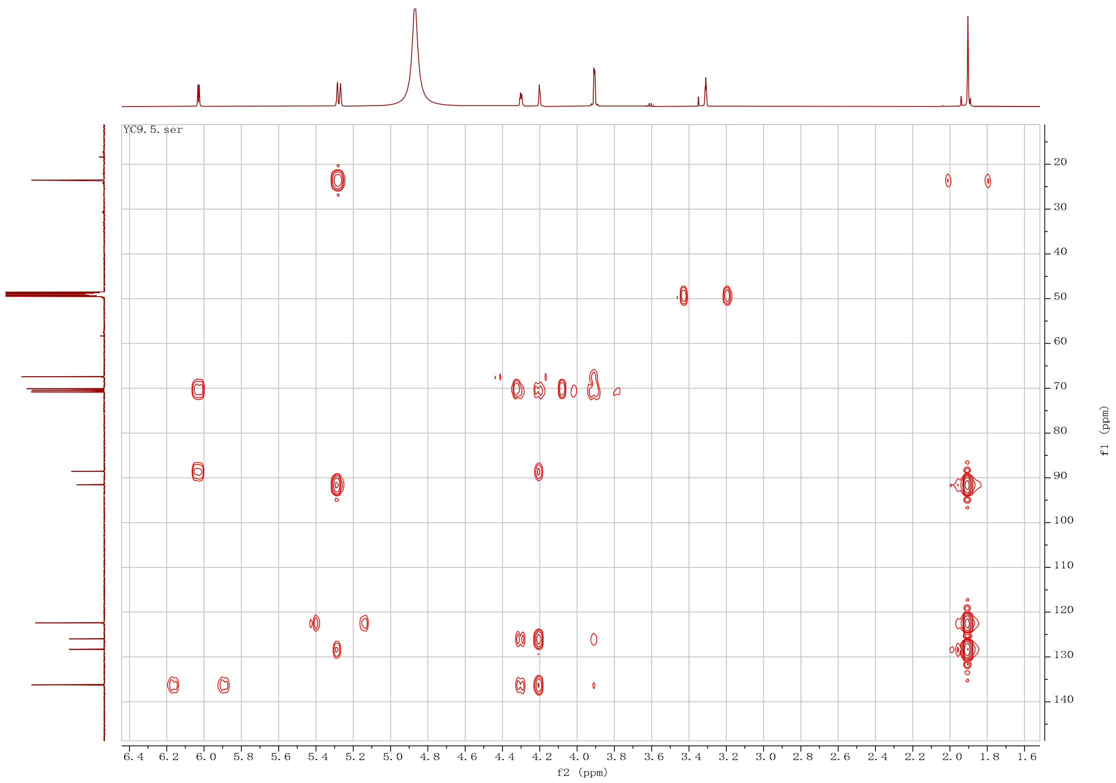

Figure S34. ^{1}H ^{1}H COSY and HMBC spectra (600 MHz, CD_3OD) of **7**

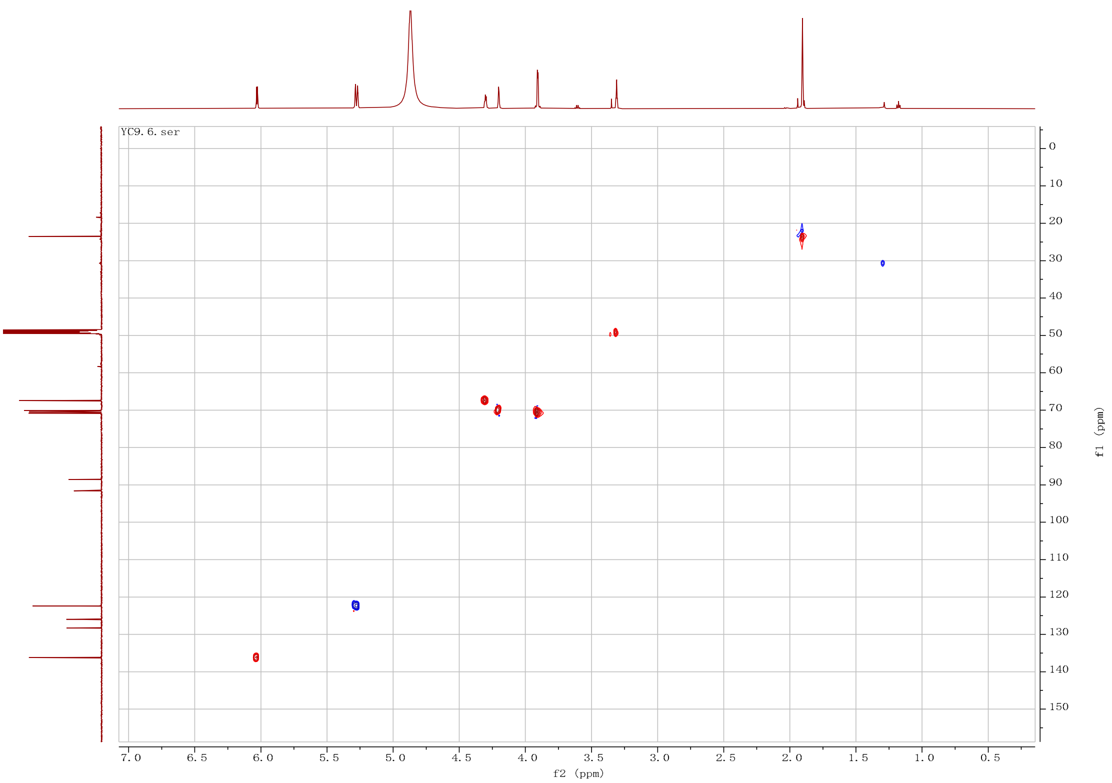

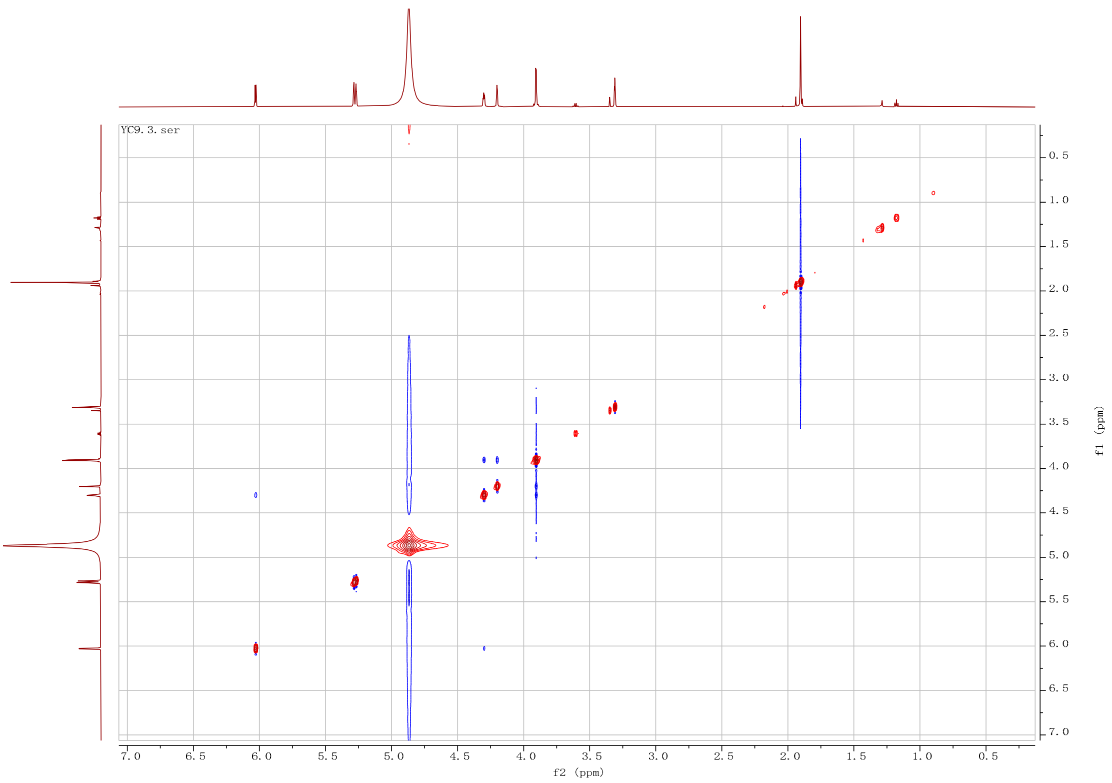

Figure S35. HMQC and NOESY spectra (600 MHz, CD_3OD) of **7**

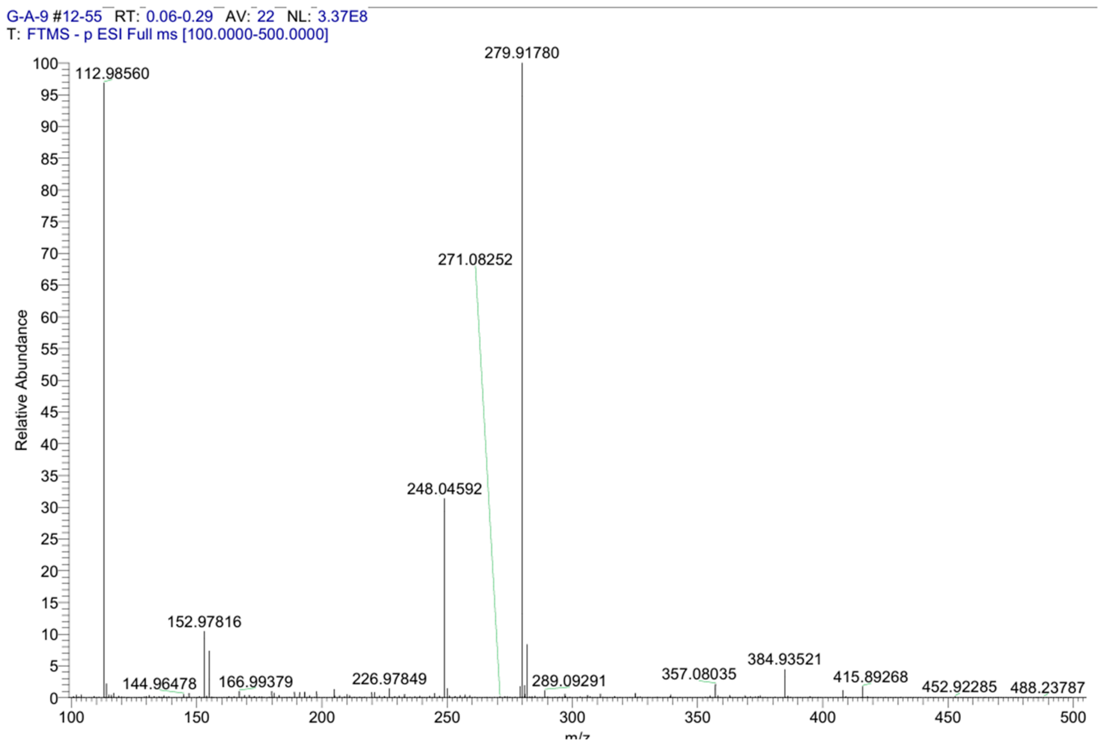

Figure S36. HRESIMS spectrum of **8**

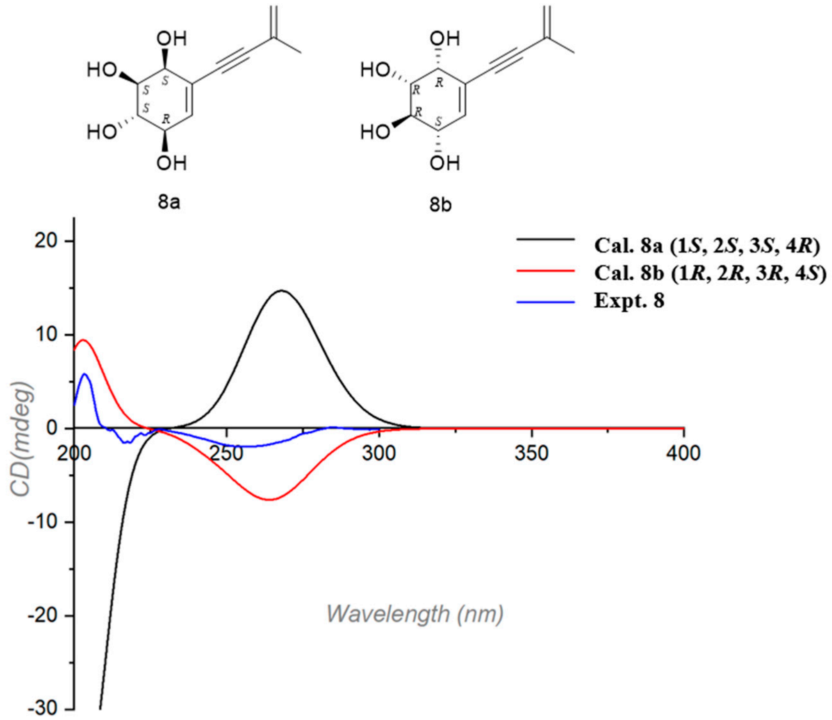

Figure S37. Structures and experimental ECD spectra of**8**

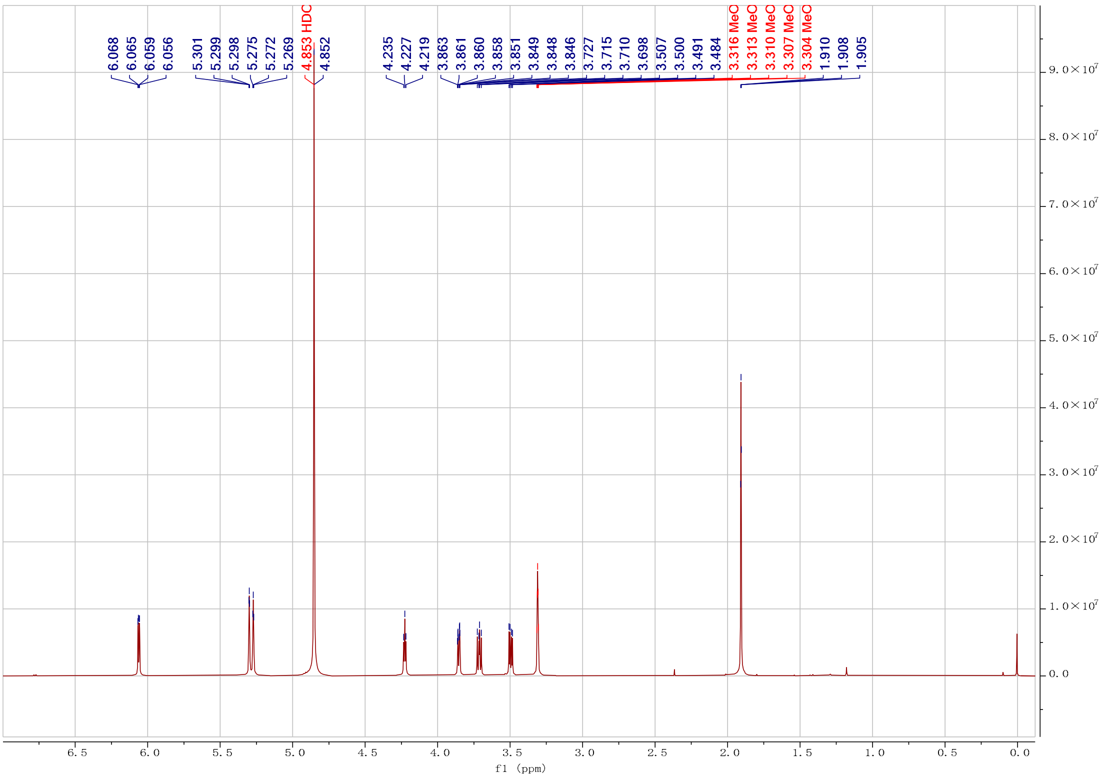

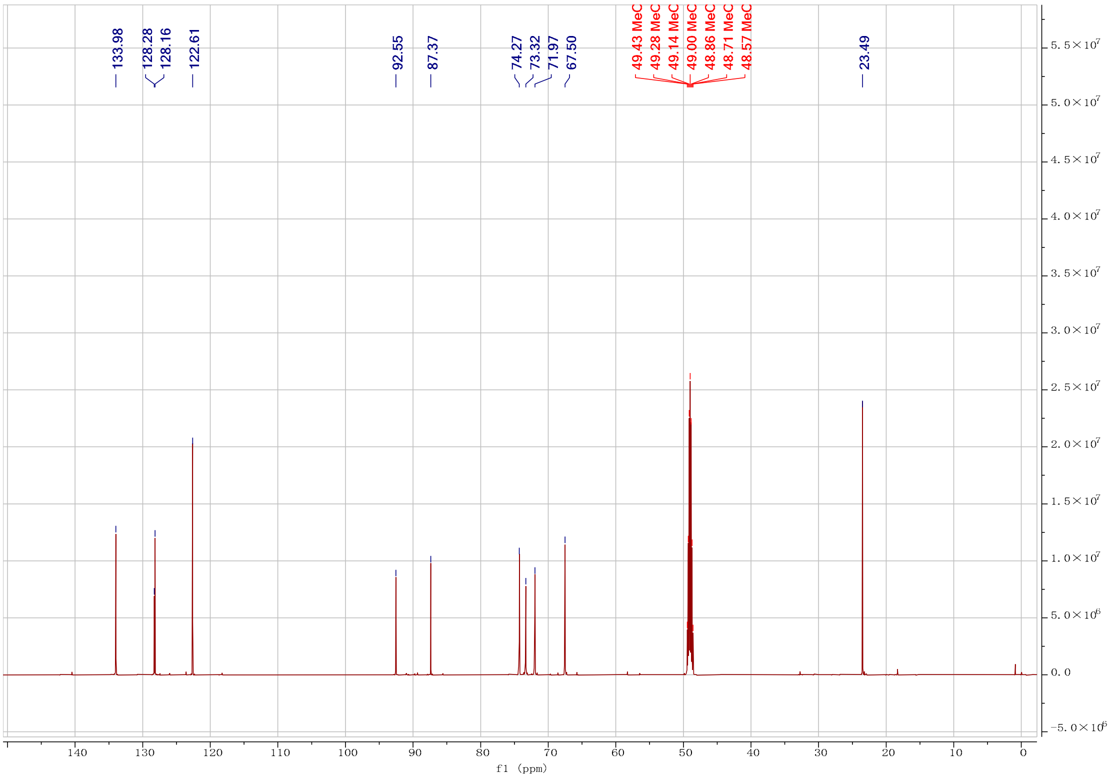

Figure S38. ^{1}H (600 MHz) and ^{13}C (150 MHz) NMR data of **8** in CD_3OD

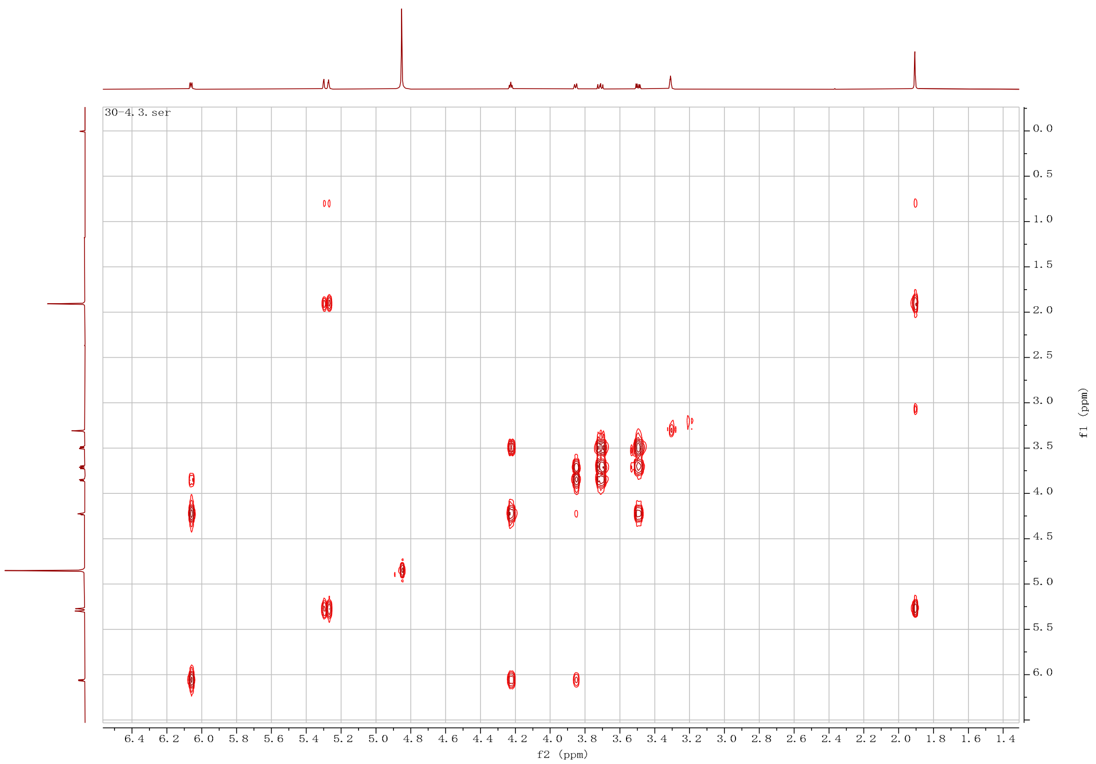

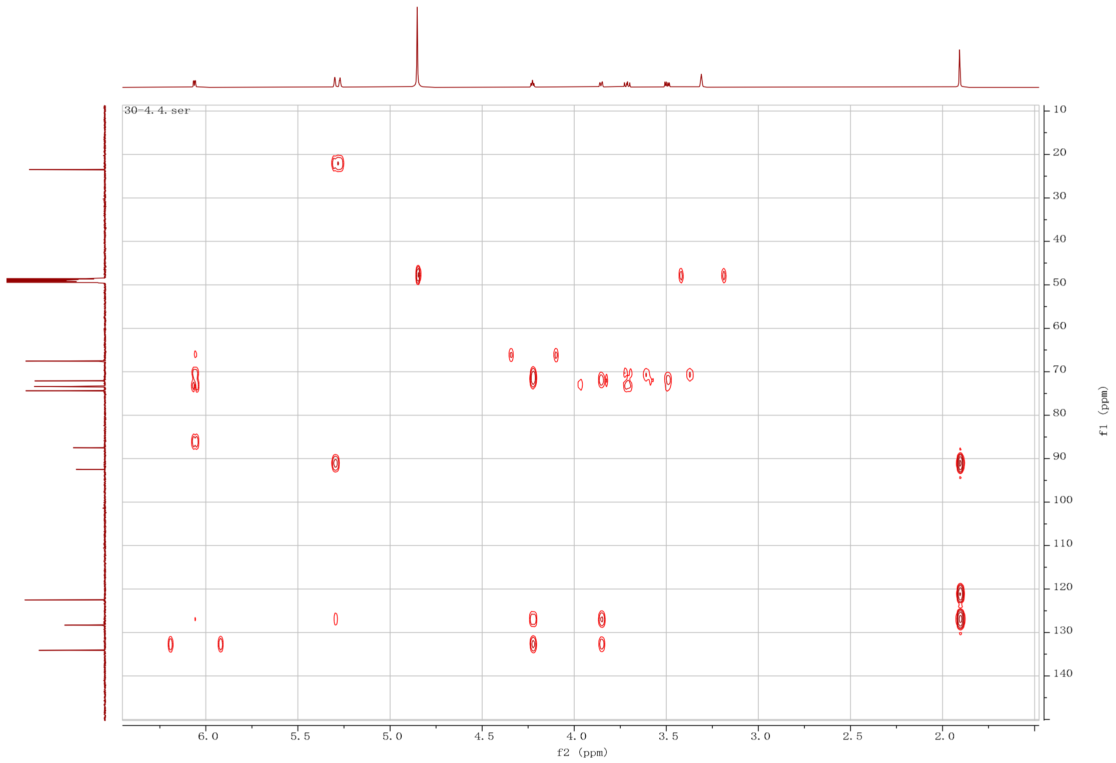

Figure S39. ${}^{1}H$ ${}^{1}H$ COSY and HMBC spectra (600 MHz, CD_3OD) of **8**

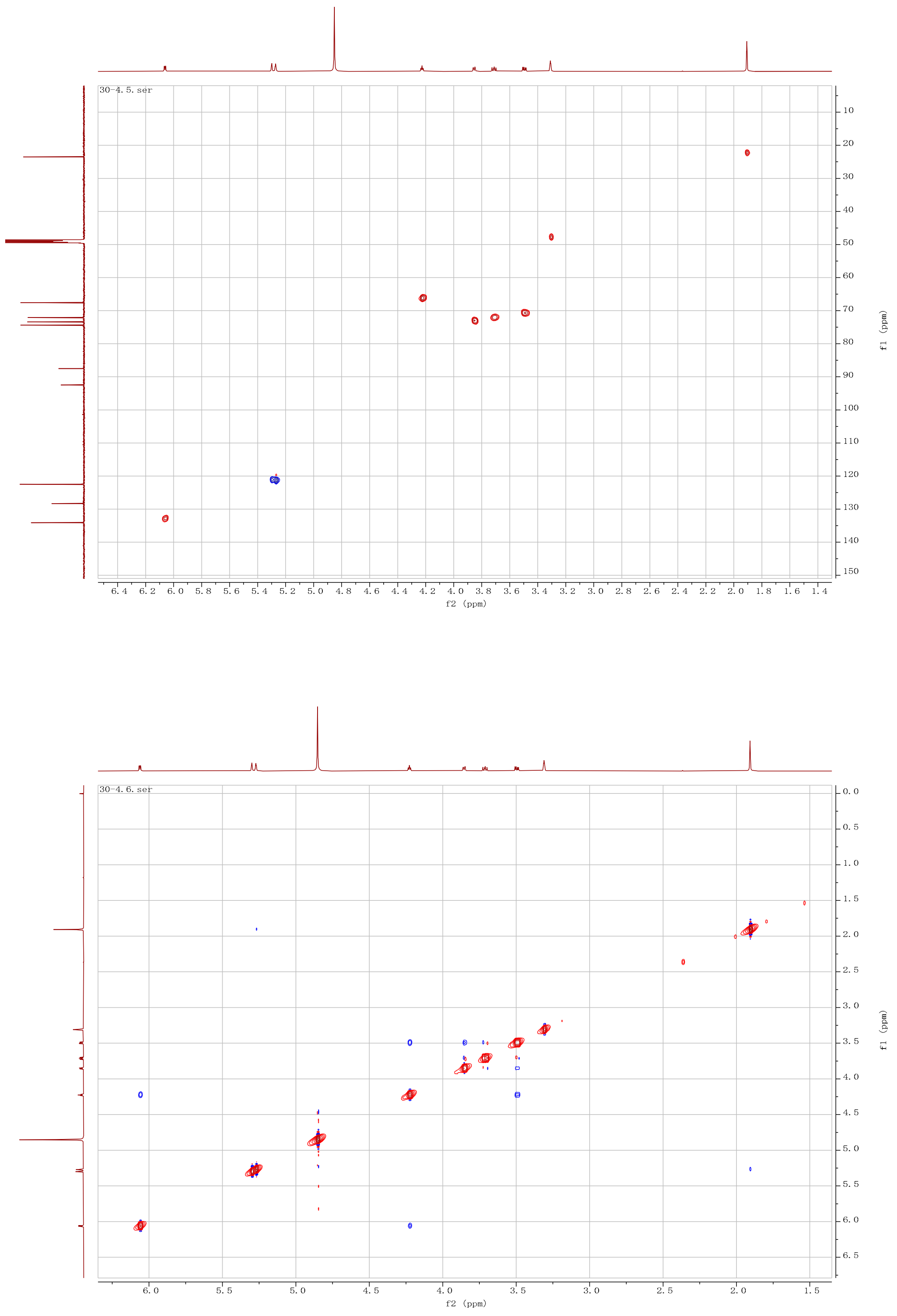

Figure S40. HMQC and NOESY spectra (600 MHz, CD_3OD) of **8**

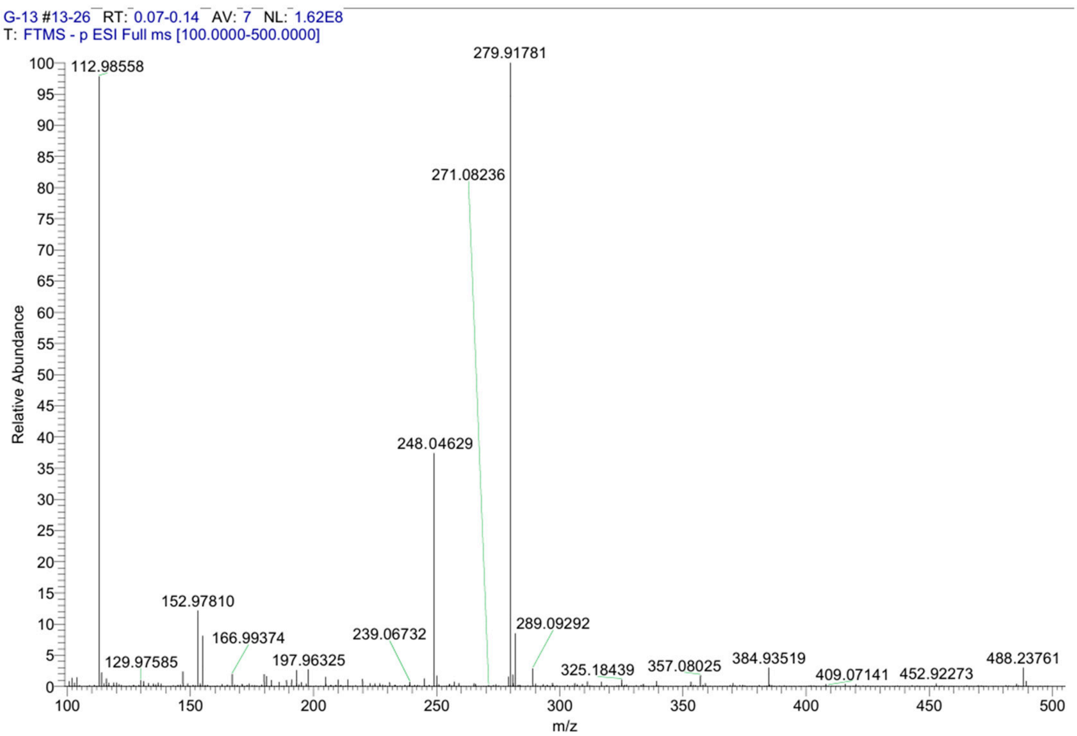

Figure S41. HRESIMS spectrum of **9**

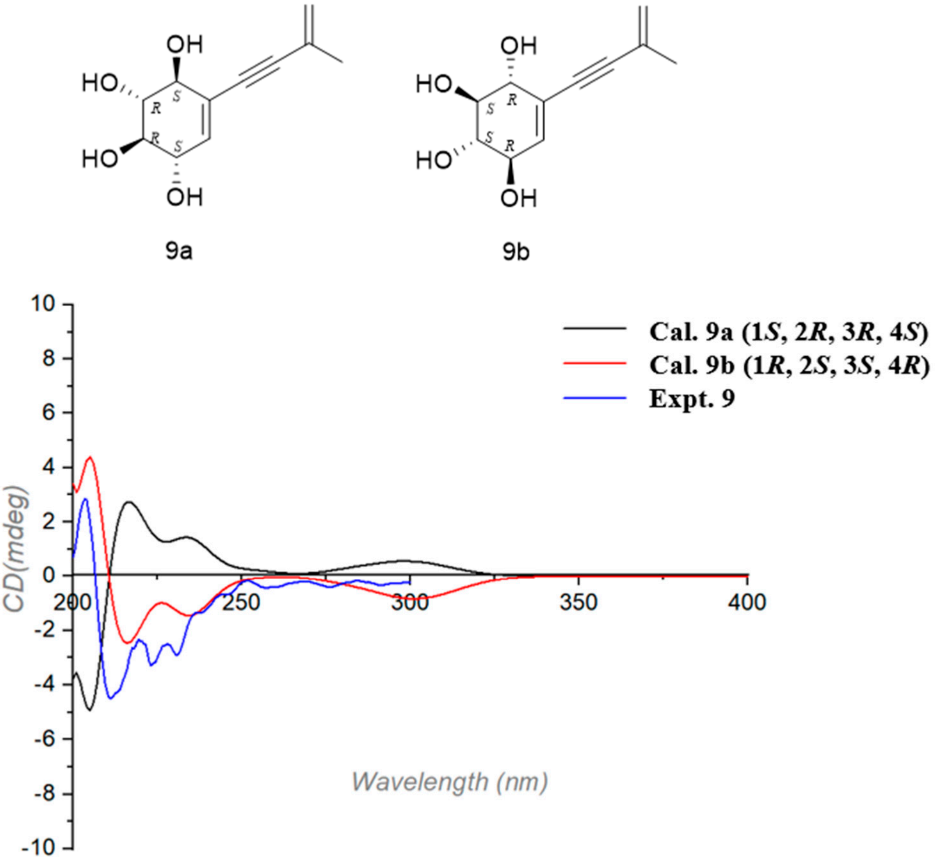

Figure S42. Structures and experimental ECD spectra of **9**

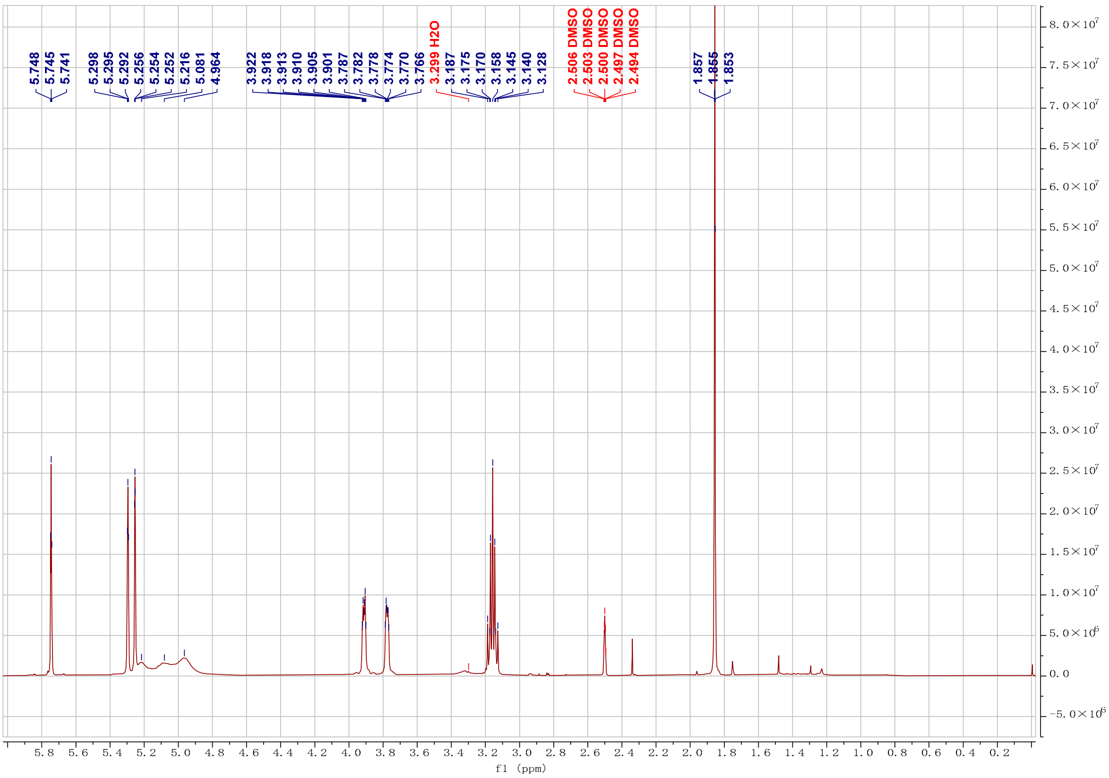

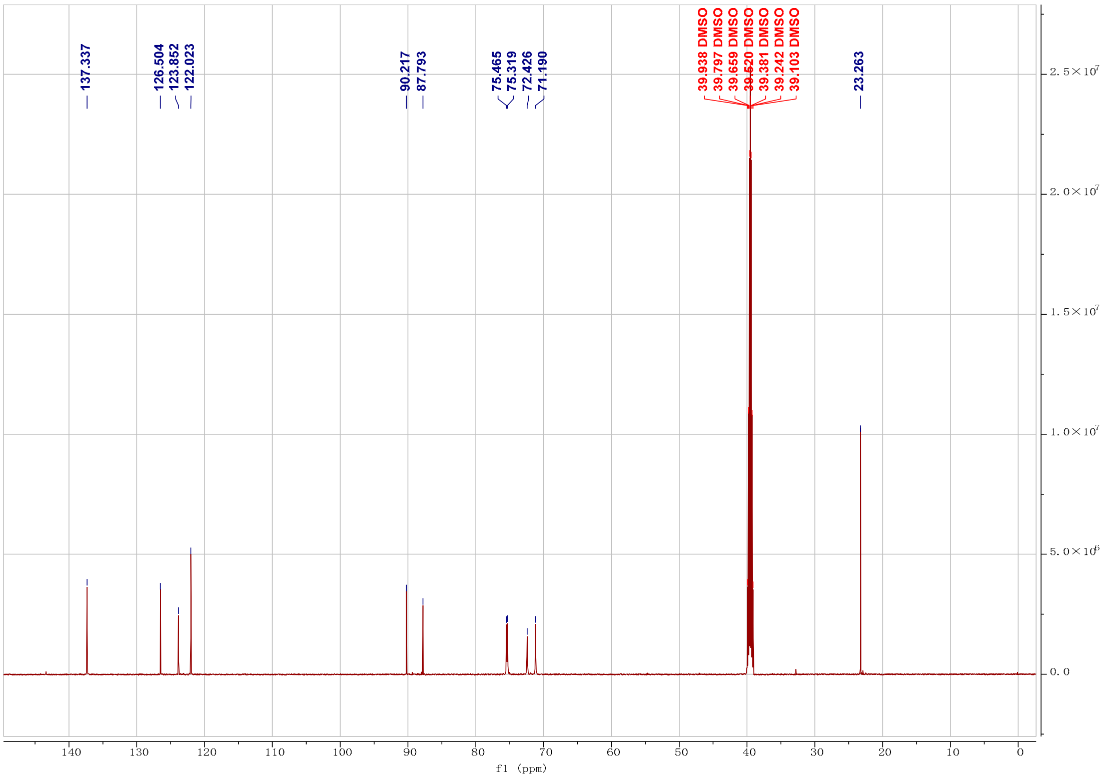

Figure S43. ^{1}H (600 MHz) and ^{13}C (150 MHz) NMR data of **9** in DMSO-d_6

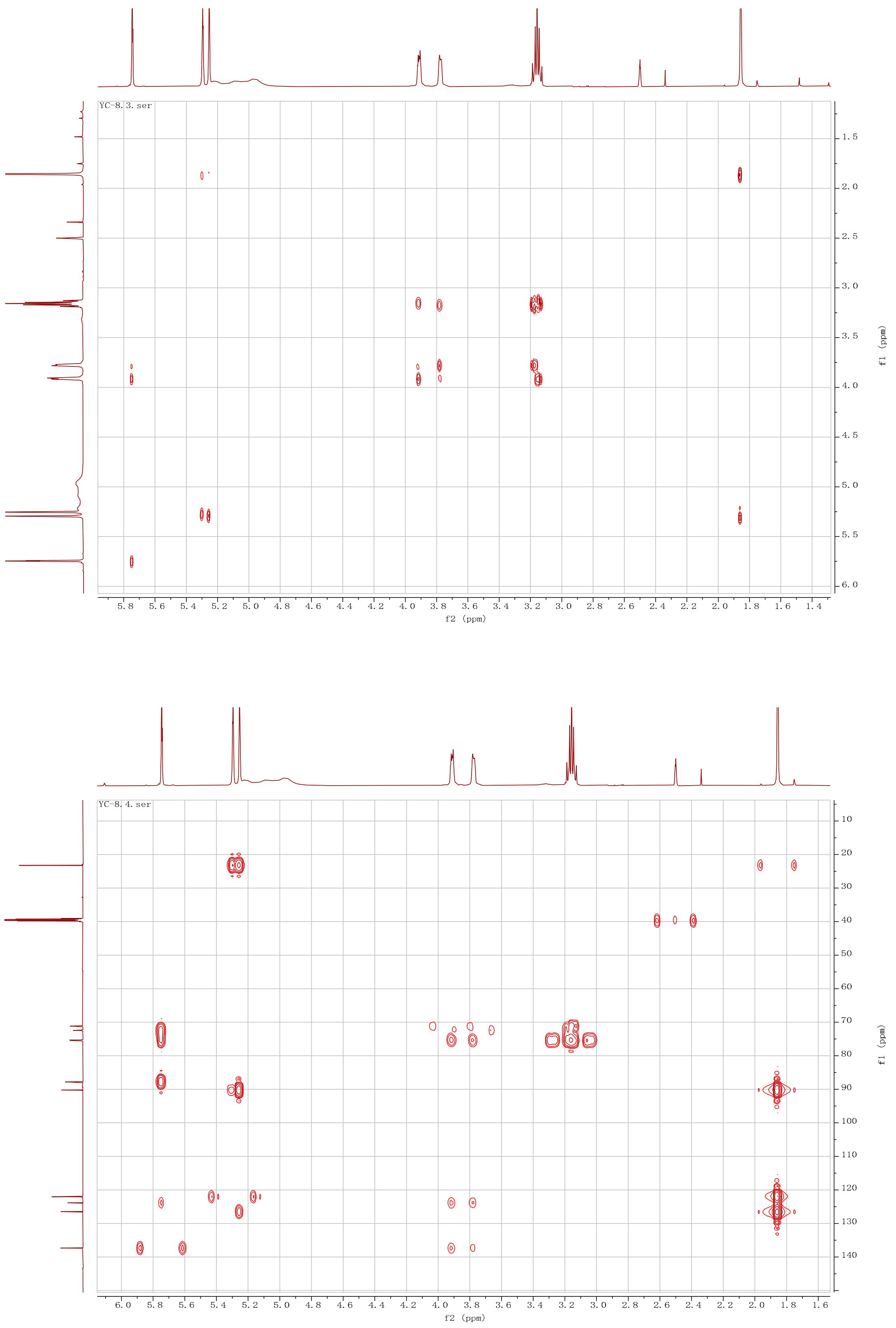

Figure S44. ^{1}H ^{1}H COSY and HMBC spectra (600 MHz, DMSO-d_6) of **9**

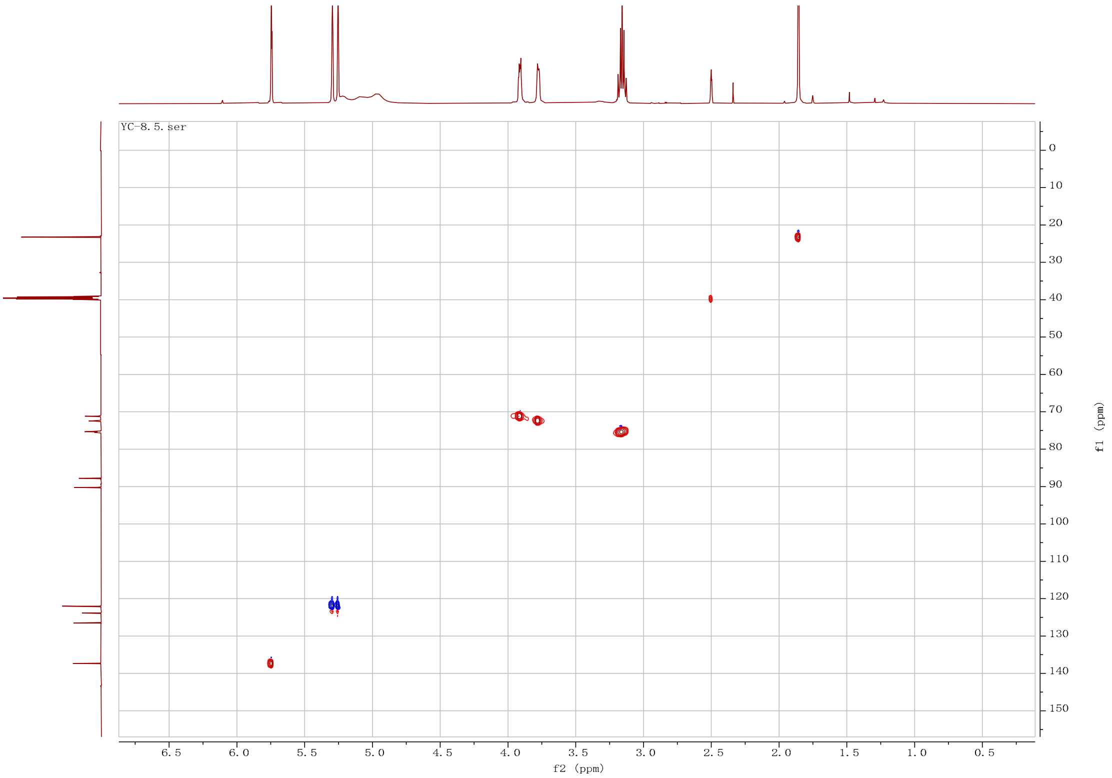

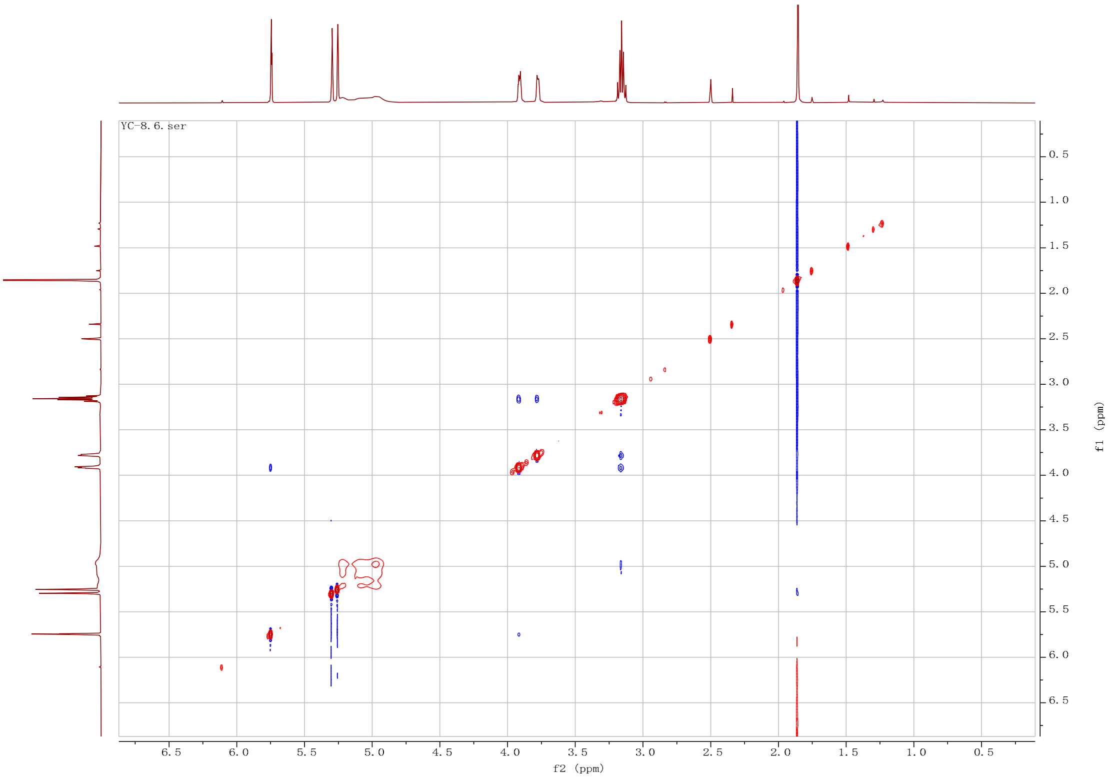

Figure S45. HMQC and NOESY spectra (600 MHz, DMSO-d_6) of **9**

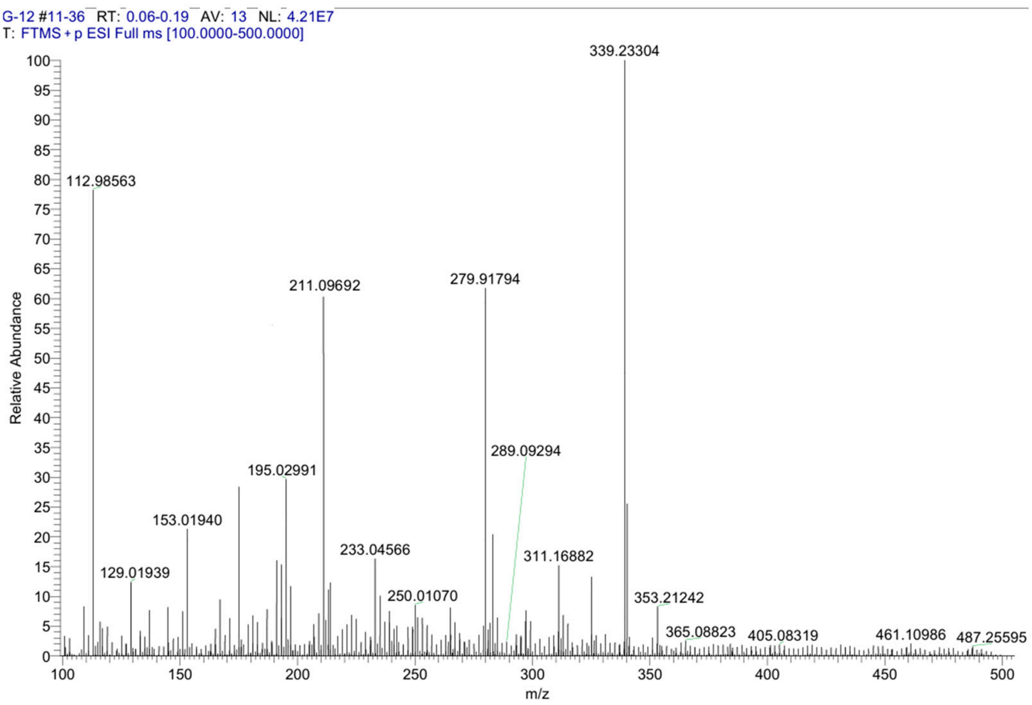

Figure S46. HRESIMS spectrum of **10**

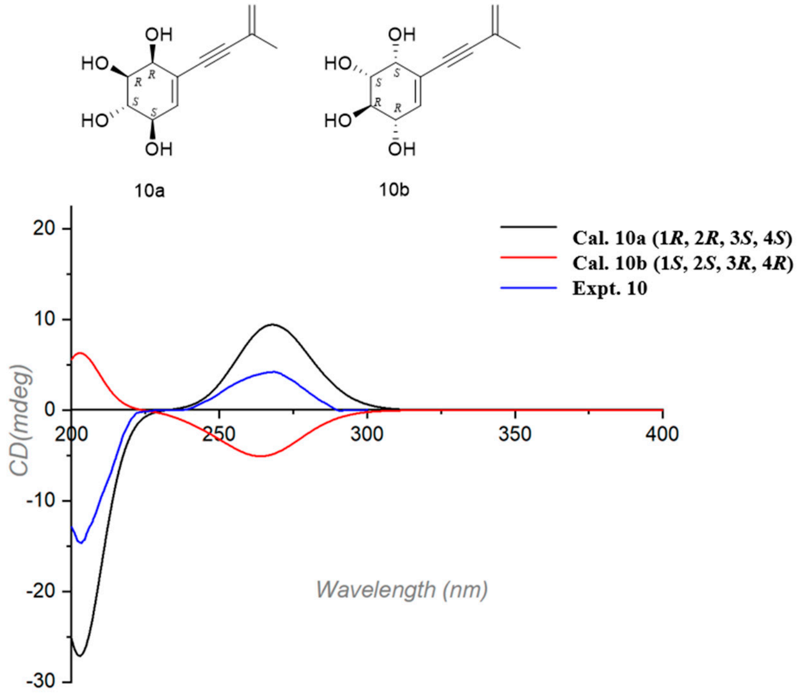

Figure S47. Structures and experimental ECD spectra of **10**

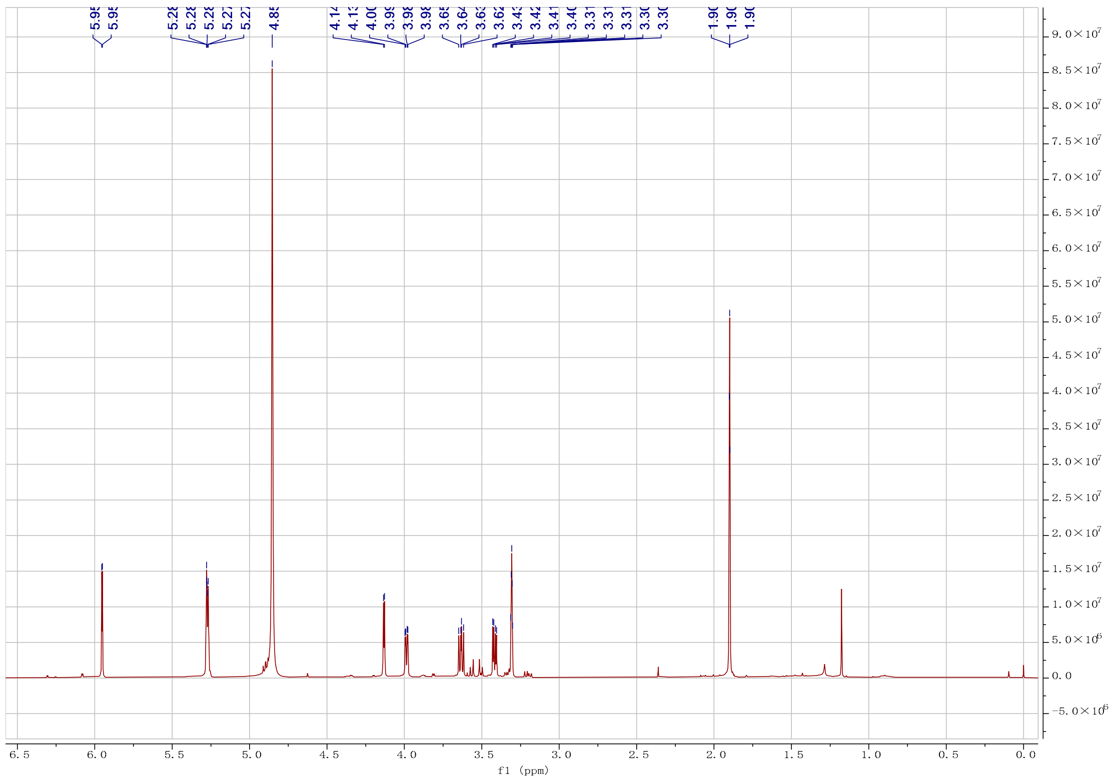

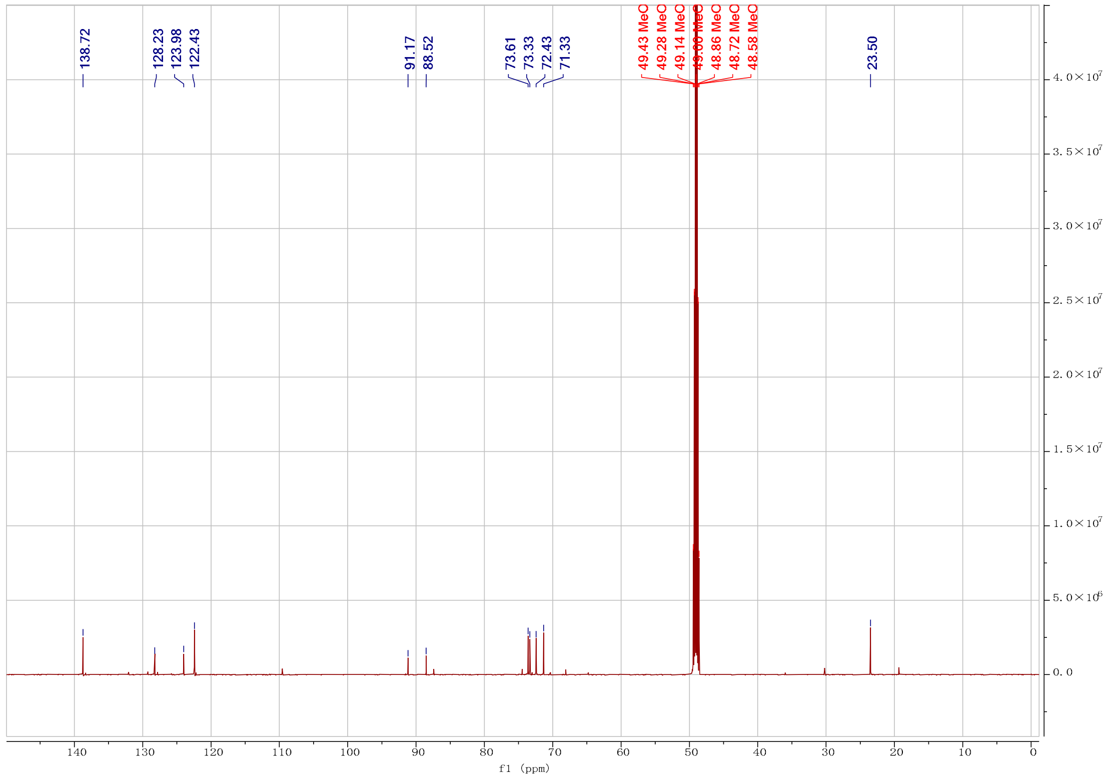

Figure S48. ^{1}H (600 MHz) and ^{13}C (150 MHz) NMR data of **10** in CD_3OD

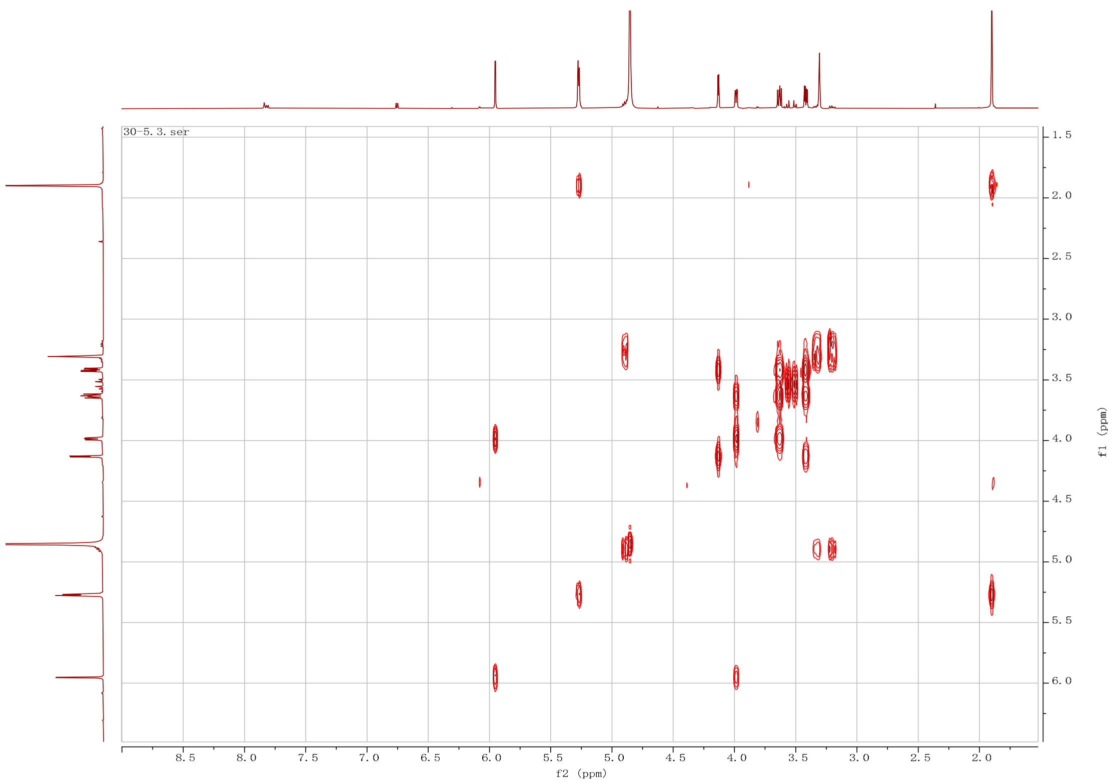

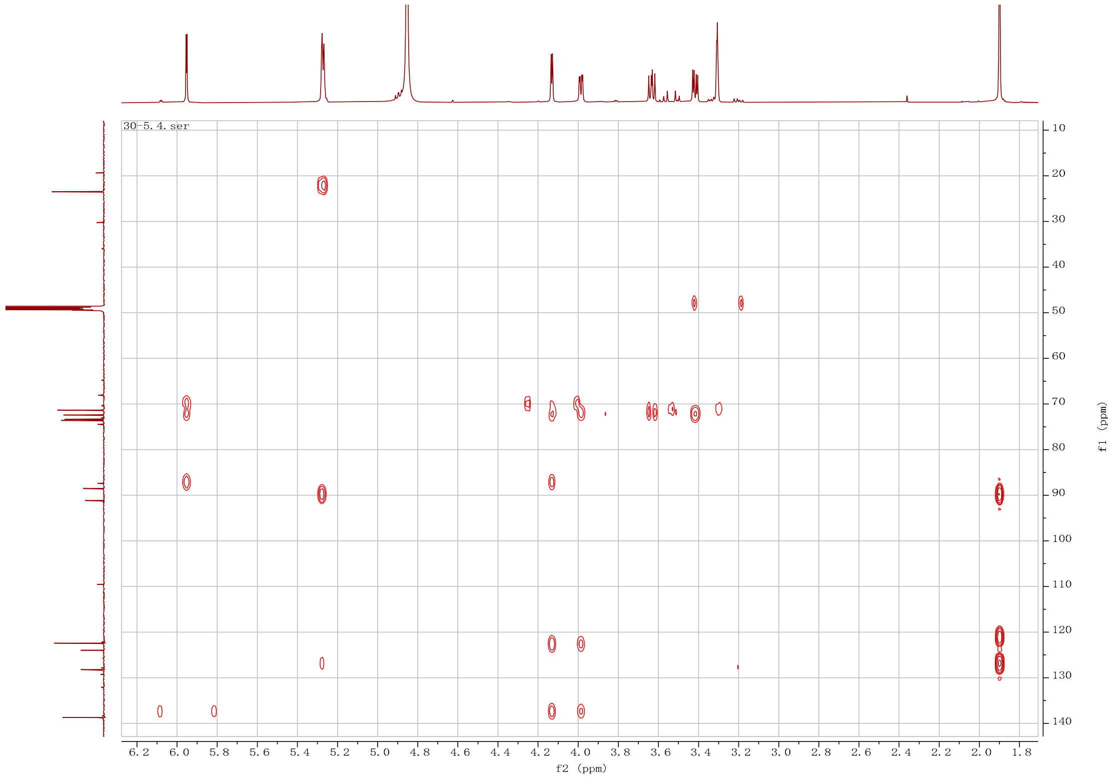

Figure S49. ^{1}H ^{1}H COSY and HMBC spectra (600 MHz, CD_3OD) of **10**

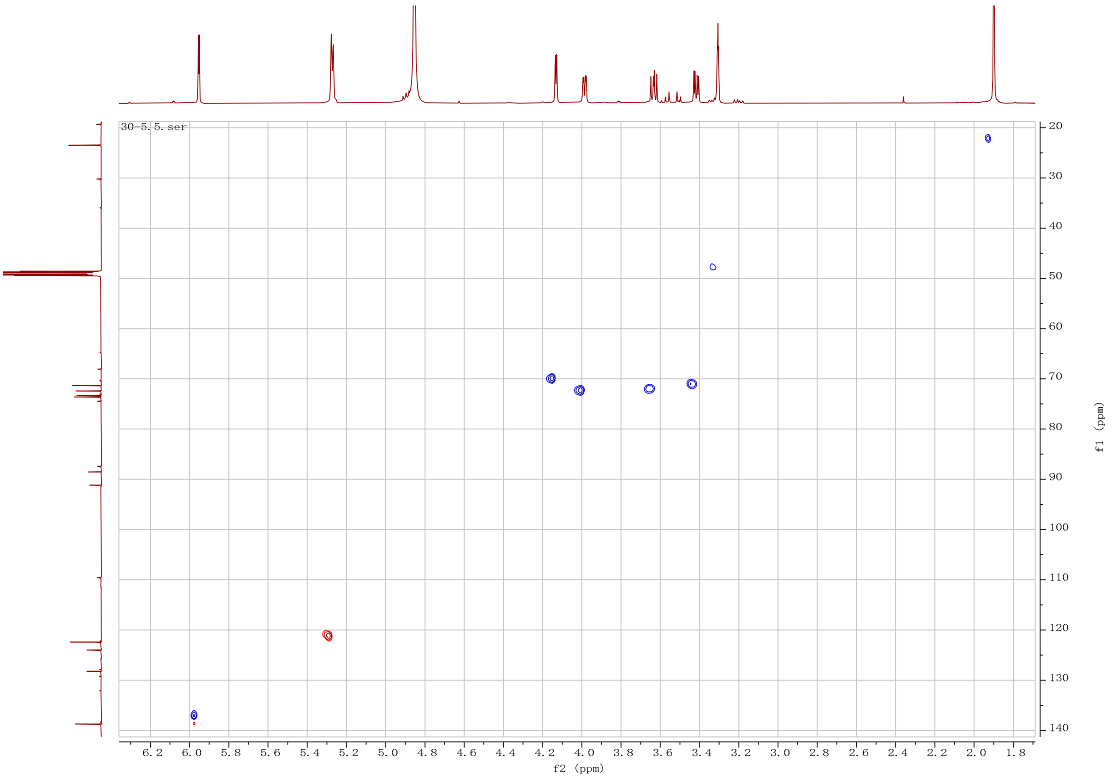

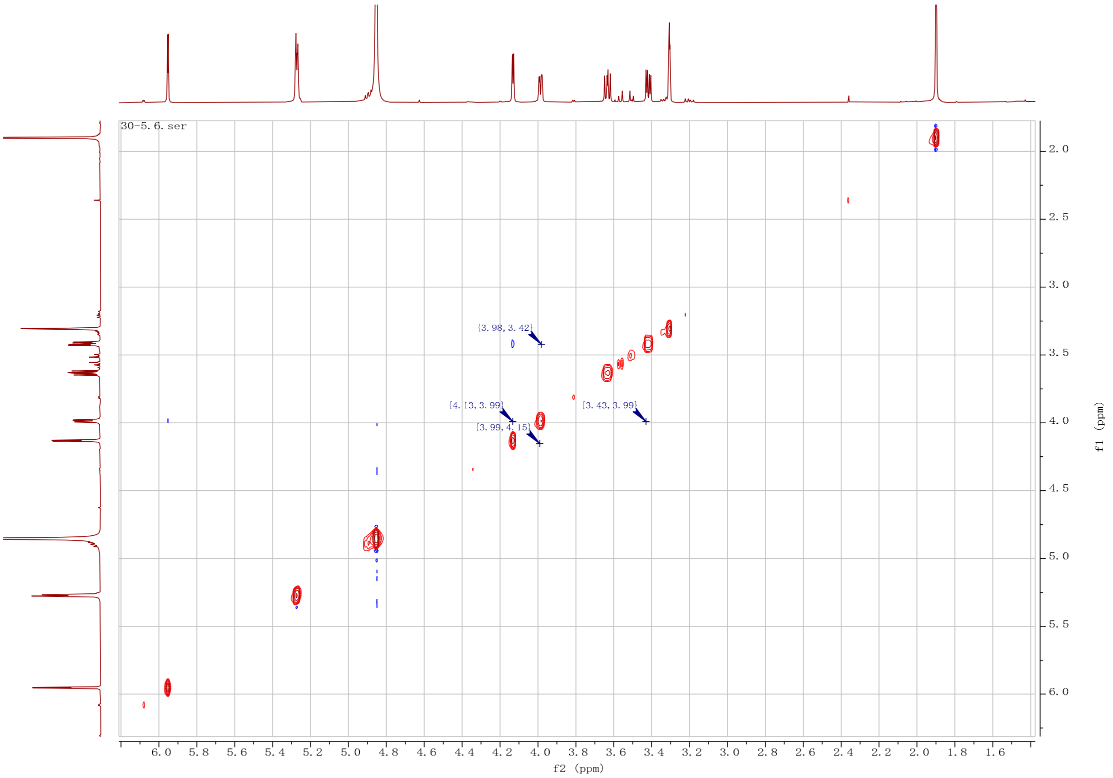

Figure S50. HMQC and NOESY spectra (600 MHz, CD_3OD) of **10**

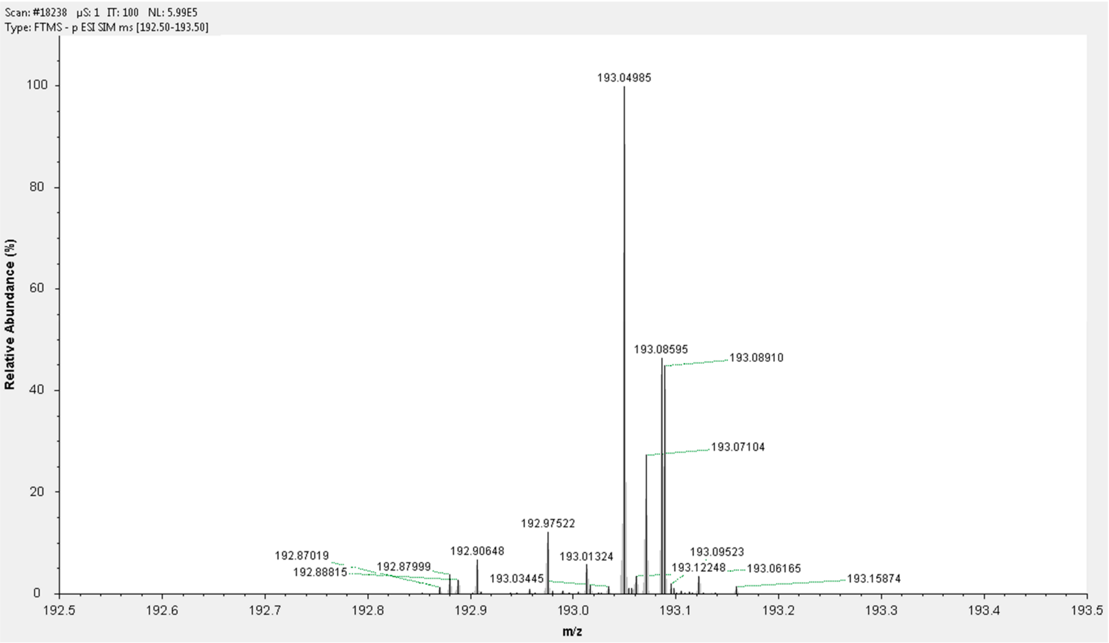

Figure S51. HRESIMS spectrum of **11**

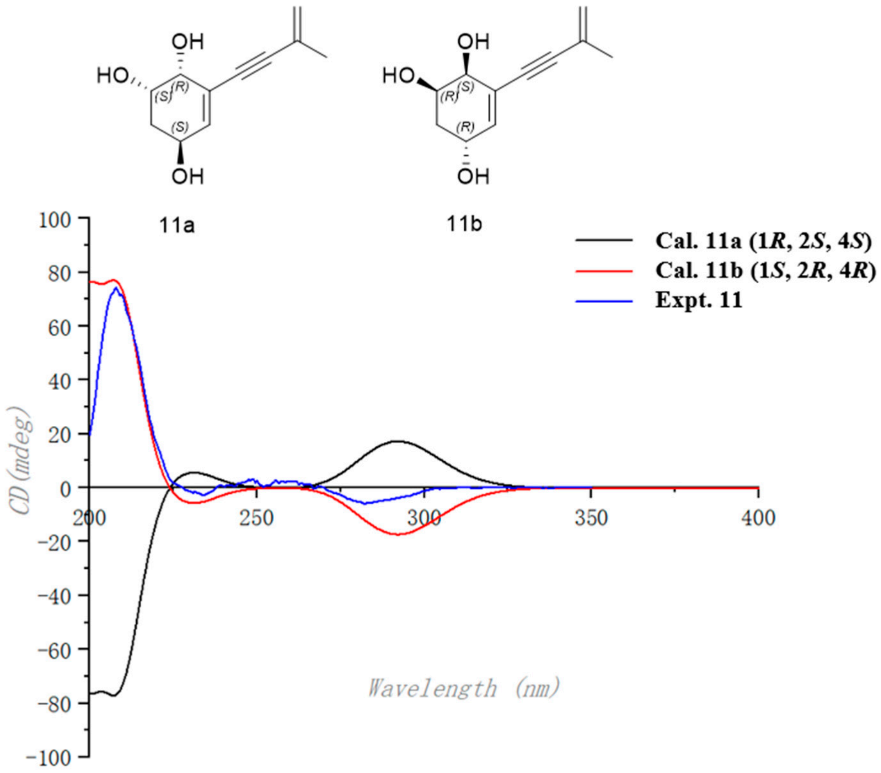

Figure S52. Structures and experimental ECD spectra of **11**

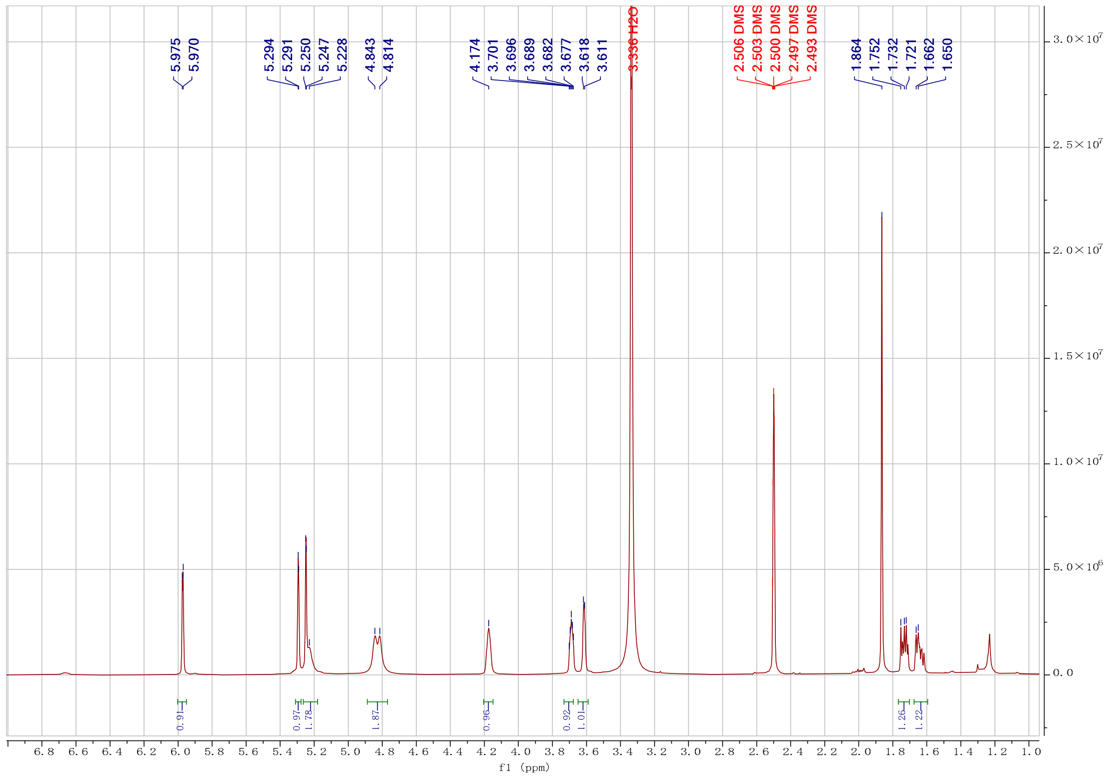

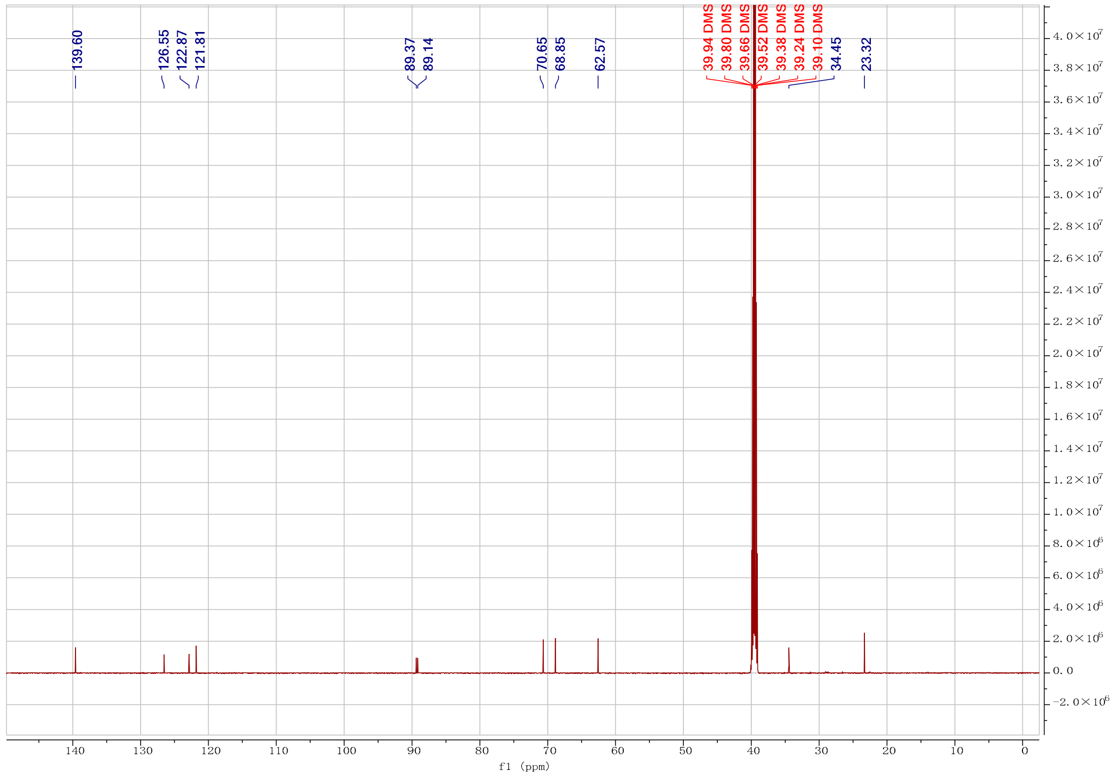

Figure S53. ^{1}H (600 MHz) and ^{13}C (150 MHz) NMR data of **11** in DMSO-d_6

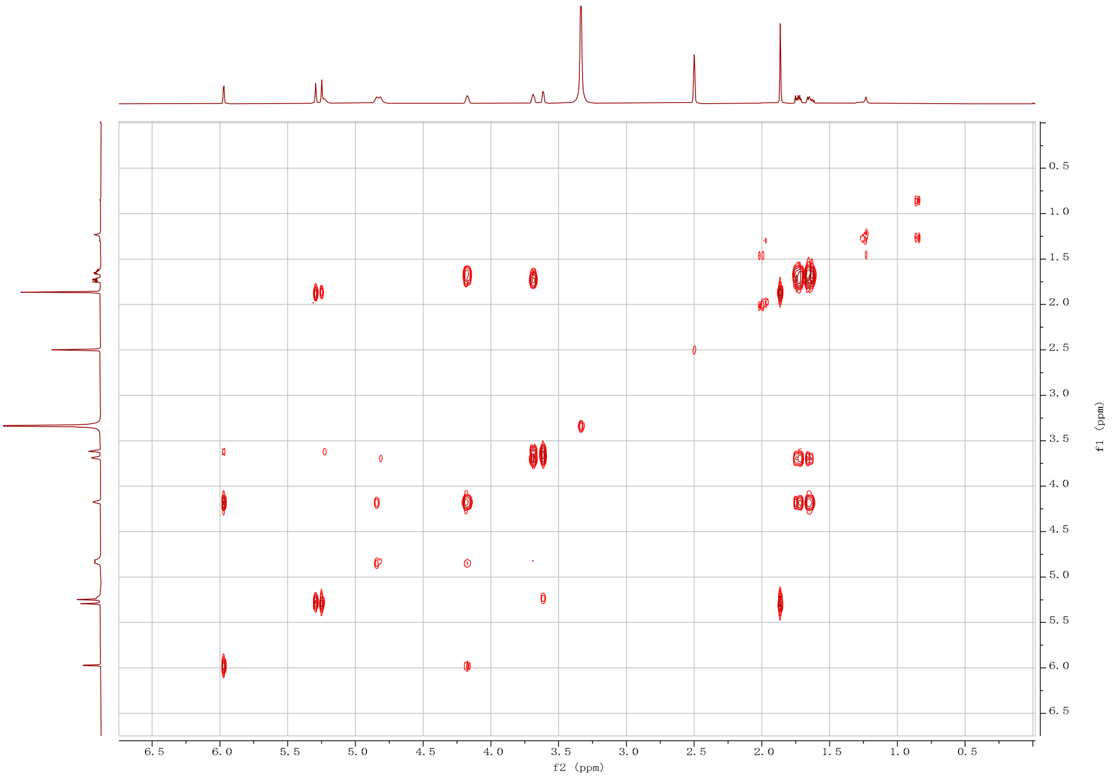

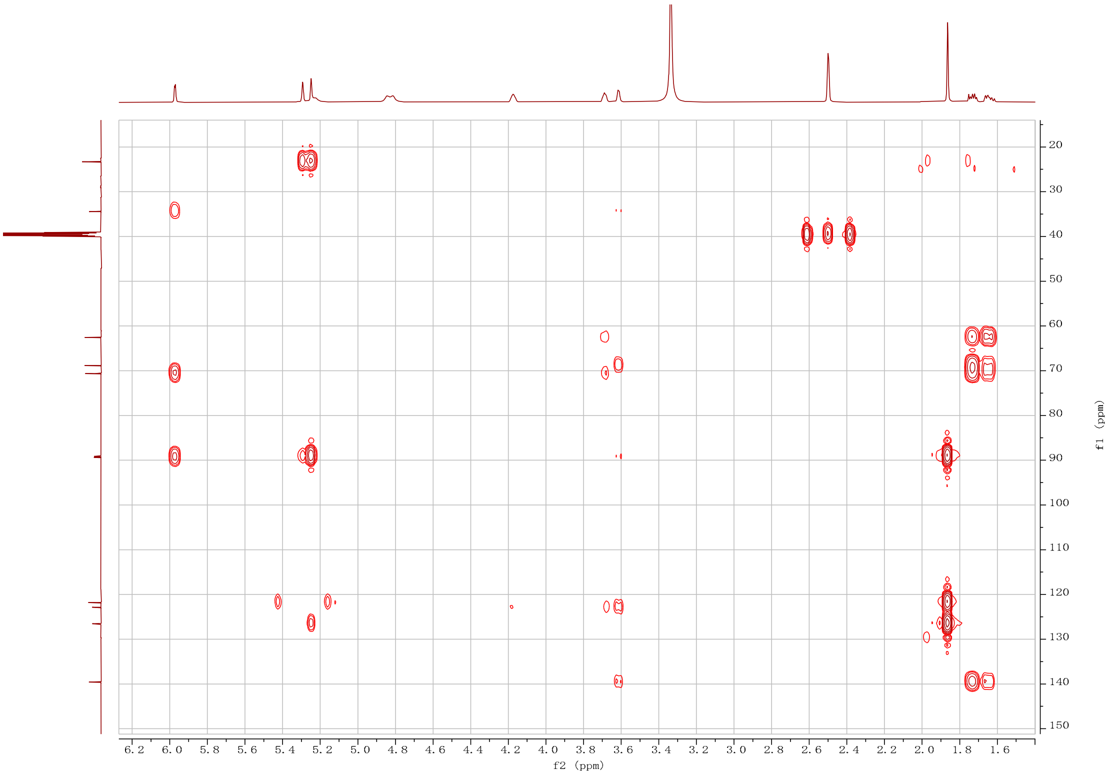

Figure S54. ^{1}H ^{1}H COSY and HMBC spectra (600 MHz, DMSO-d_6) of **11**

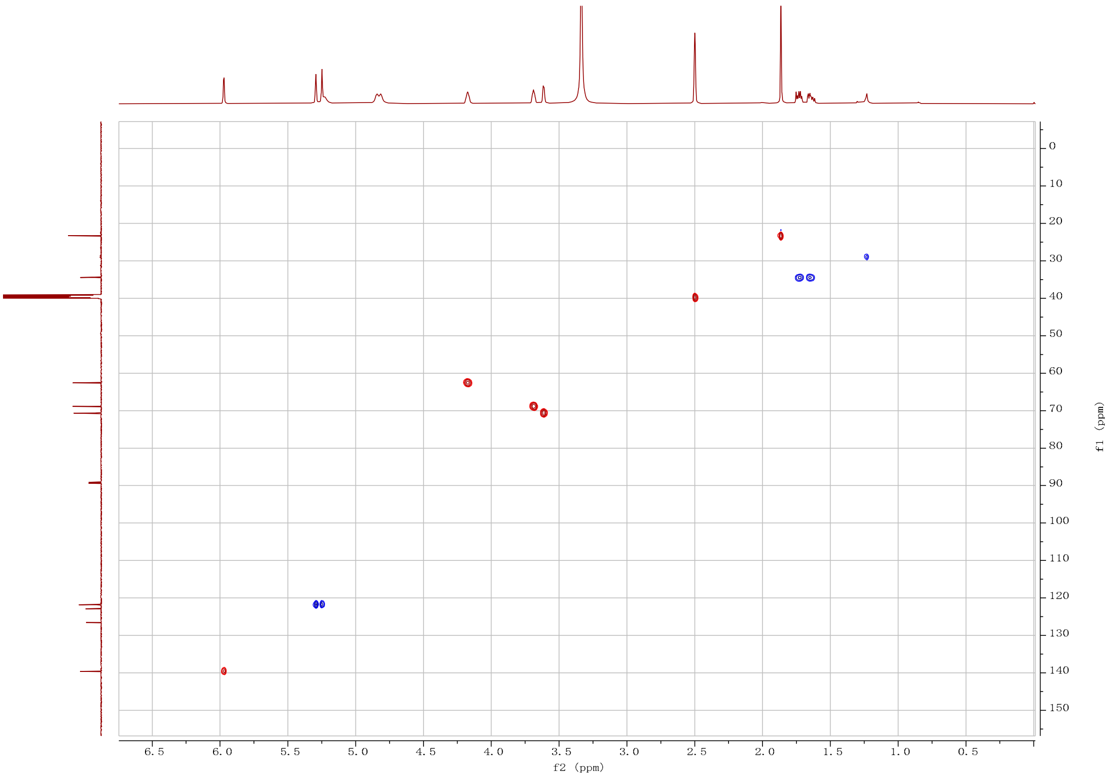

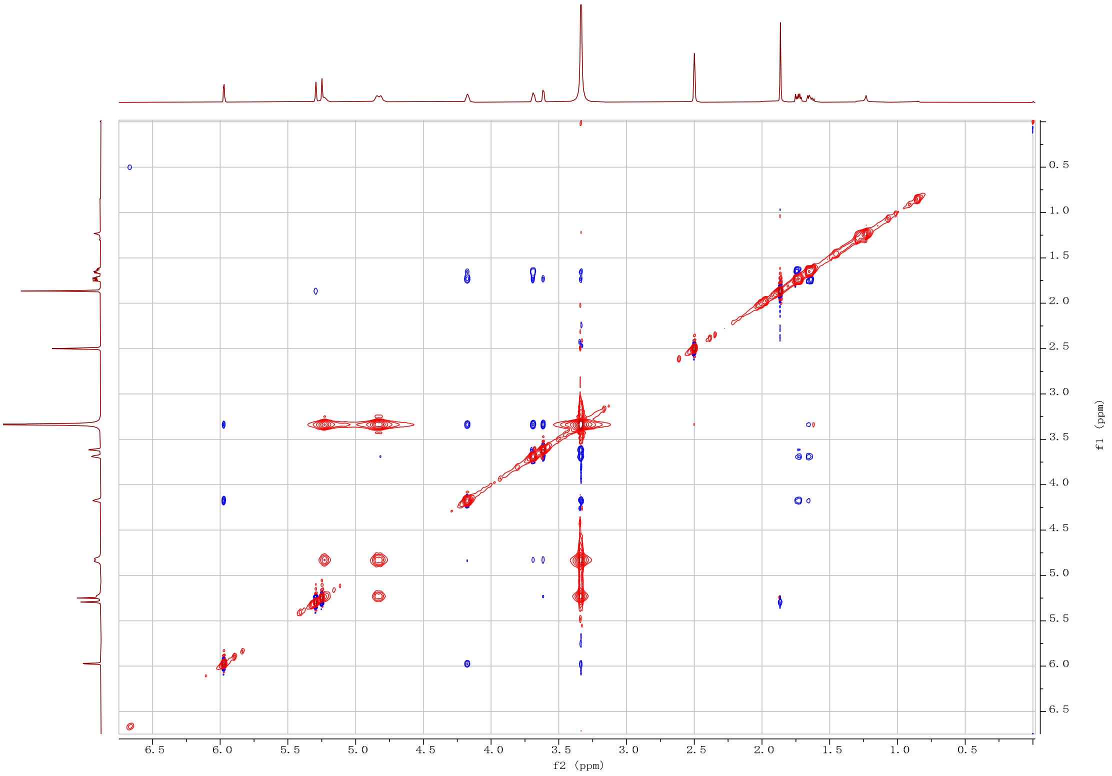

Figure S55. HMQC and NOESY spectra (600 MHz, DMSO-d_6) of **11**

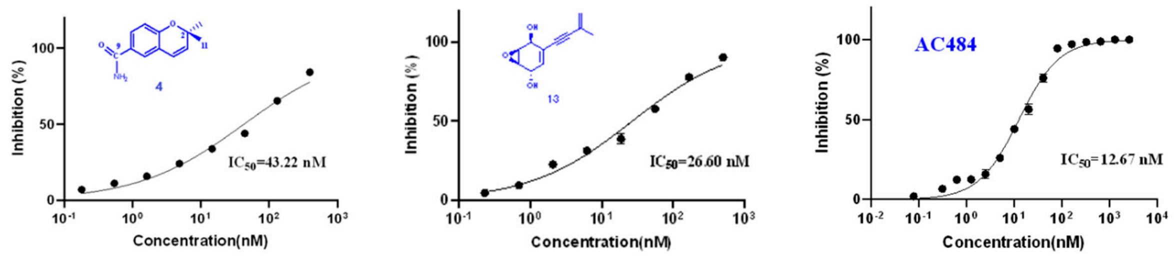

Figure S56. The IC_{50} curves of compounds **4, 13** and AC484 against TCPTP

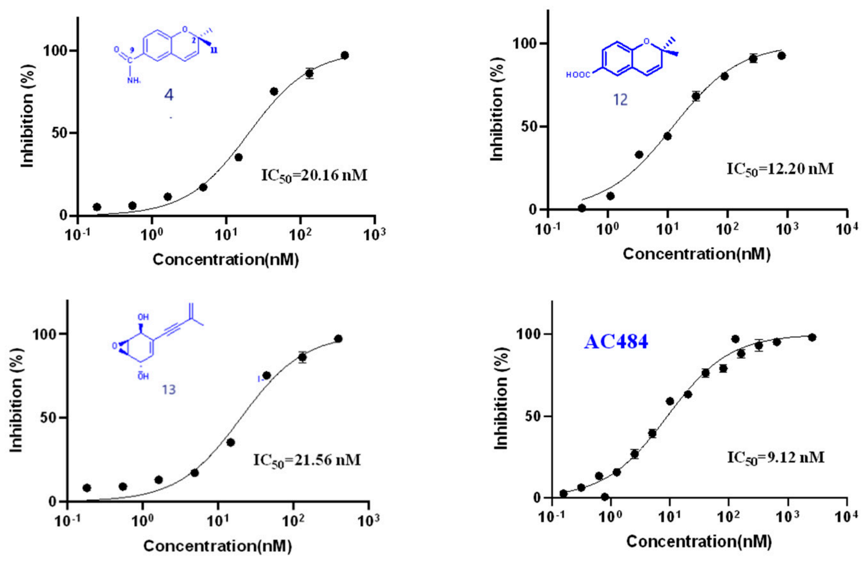

Figure S57. The IC_{50} curves of compounds **4, 12, 13** and AC484 against PTP1B

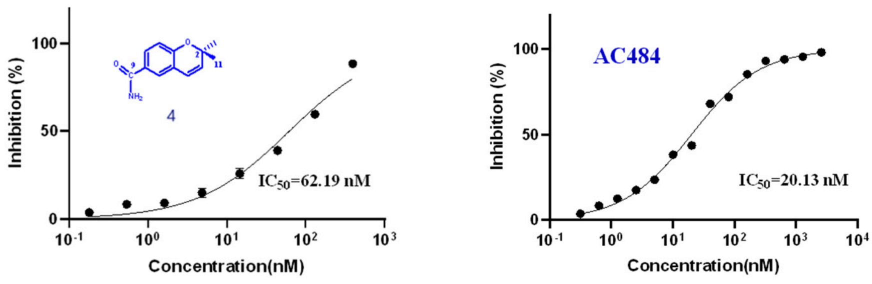

Figure S58. The IC_{50} curves of compounds **4** and AC484 against MEG2

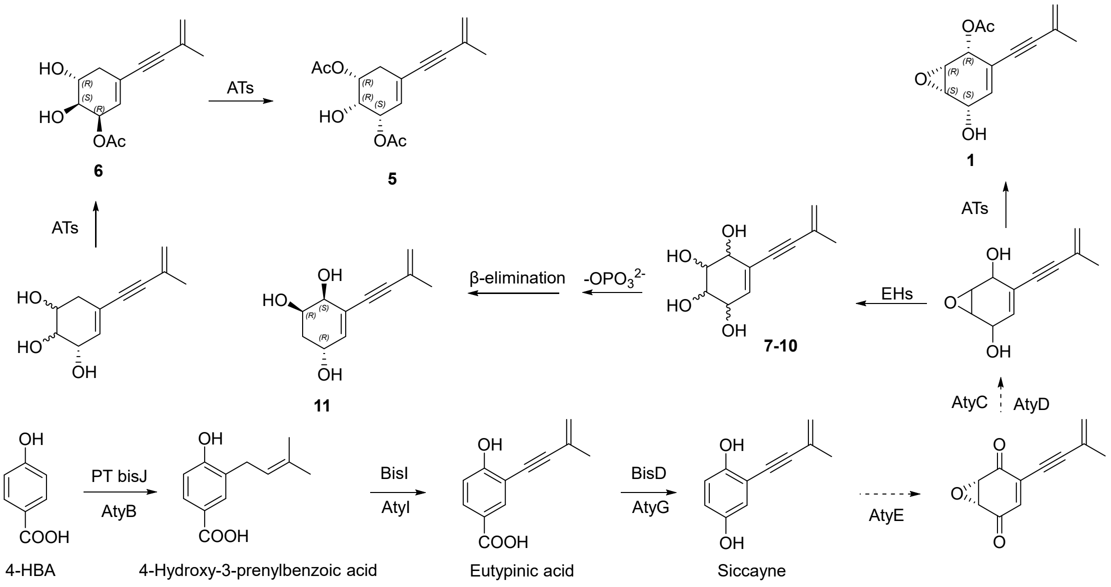

Figure S59. Possible biosynthetic pathway for compounds **1** and **5-11**.

Table S1. Binding energies of the top 10 conformations.

Rank	Binding Energy (kcal/mol)
1	-6.2
2	-5.7
3	-5.7
4	-5.5
5	-5.5
6	-5.3
7	-5.1
8	-5.1
9	-5.1
10	-5.0
